# Effects of IL-1β–Blocking Therapies in Type 2 Diabetes Mellitus: A Quantitative Systems Pharmacology Modeling Approach to Explore Underlying Mechanisms

**DOI:** 10.1038/psp.2014.16

**Published:** 2014-06-11

**Authors:** R Palmér, E Nyman, M Penney, A Marley, G Cedersund, B Agoram

**Affiliations:** 1 Wolfram MathCore AB, Linköping, Sweden; 2 Department of Clinical and Experimental Medicine, Linköping University, Linköping, Sweden; 3 Department of Clinical Pharmacology, Drug Metabolism, and Pharmacokinetics, MedImmune, Cambridge, UK; 4 Bioscience, Astra Zeneca, Alderley Park, UK; 5 Department of Biomedical Engineering, Linköping University, Linköping, Sweden

## Abstract

Recent clinical studies suggest sustained treatment effects of interleukin-1β (IL-1β)–blocking therapies in type 2 diabetes mellitus. The underlying mechanisms of these effects, however, remain underexplored. Using a quantitative systems pharmacology modeling approach, we combined *ex vivo* data of IL-1β effects on β-cell function and turnover with a disease progression model of the long-term interactions between insulin, glucose, and β-cell mass in type 2 diabetes mellitus. We then simulated treatment effects of the IL-1 receptor antagonist anakinra. The result was a substantial and partly sustained symptomatic improvement in β-cell function, and hence also in HbA1C, fasting plasma glucose, and proinsulin–insulin ratio, and a small increase in β-cell mass. We propose that improved β-cell function, rather than mass, is likely to explain the main IL-1β–blocking effects seen in current clinical data, but that improved β-cell mass might result in disease-modifying effects not clearly distinguishable until >1 year after treatment.

Type 2 diabetes mellitus (T2DM) is a chronic disease characterized by hyperglycemia due to multiple dysfunctions including inadequate insulin secretion, resistance to insulin action, and excessive and inappropriate glucagon secretion. There is no curative treatment for T2DM; all available therapies attempt to control hyperglycemia by attenuating one or more of the pathophysiological pathways—reducing insulin resistance and thereby increasing glucose processing and/or curtailing excessive glucose release through glucagon action. These symptomatic therapies, while retarding progression of T2DM-related complications, are still unable to prevent eventual retinal, neural, and other complications. Therefore, there is a concerted effort within research and industry circles to develop therapies that cure patients of T2DM.

One approach to the reversal and potential cure of T2DM is through revival of pancreatic β-cells, which are the primary producers of insulin in the body, and whose mass and function are highly curtailed during T2DM. A number of publications (see Donath *et al*.^1^ for a review) have recently put forward interleukin-1β (IL-1β)–induced IL-1 signaling as a promising target for β-cell regeneration. *Ex vivo* data have shown IL-1β expression to be highly upregulated in pancreatic islets of patients with T2DM^2^ and human β-cells to be prone to both IL-1β–induced destruction and functional impairment,^3,4^ indicating a possible role of IL-1β in T2DM progression. Moreover, following from some of these *ex vivo* studies, several clinical studies investigating the effect of blocking IL-1β in T2DM have been performed.^5,6,7,8,9,10,11^ Of these, a double-blind, randomized, clinical study aimed to evaluate the role of the recombinant human form of the endogenous IL-1 receptor antagonist—anakinra—in 70 patients with overt T2DM,^5^ is of particular interest. In this study, 13 weeks of daily subcutaneous administration of 200-mg anakinra resulted in a mean reduction of 0.46% points in glycated hemoglobin (HbA1c) compared with placebo, as well as improvements in stimulated C-peptide secretion and the proinsulin/insulin (PI/I) ratio. Interestingly, patients that responded with a reduction in HbA1c after the 13 weeks of treatment also showed sustained improvements in stimulated C-peptide secretion and PI/I ratio, as well as in insulin dependence, C-reactive protein, and IL-6, after a follow-up phase of 39 weeks.^6^

While the results of *ex vivo* and clinical studies on the role of IL-1β in T2DM are promising, some questions still remain to be answered including the precise mechanism of action of *in vivo* IL-1 inhibition, possible longer term outcomes with such a therapy, and the therapeutic potential of IL-1β inhibitors vs. other symptomatic therapies. In particular, the task of investigating these questions has been complicated by the complexity of linking the observed effects of IL-1β *ex vivo* to the actual *in vivo* responses. Dealing with such complexity could, however, be facilitated by the use of mathematical modeling and, in this case, by attaining a quantitative understanding of the disease processes underlying T2DM pathophysiology and the documented effect of IL-1β therapies on these pathways *ex vivo*. As reviewed by Ajmera *et al*.,^12^ mathematical models have previously been applied to investigate and describe a number of different quantitative aspects of T2DM. Of these models, many focus on short-term dynamics such as intracellular signaling dynamics^13^ and metabolism,^14^ or acute whole-body changes in response to an event,^15^ and are not useful in characterizing progression of the disease over many years. However, a few models exist that describe more long-term changes, as well as the effect of different therapeutic interventions. First to propose such a model was Topp *et al.*,^16^ who presented a simple inter-dependent system of three ordinary differential equations describing the long-term relationship between glucose, insulin, and β-cell mass. This model was later used by Ribbing *et al.*^17^ to identify parameters of tesaglitazar treatment. A similar approach has also been taken by de Winter *et al*.,^18^ who developed a disease progression model to investigate the long-term effects of pioglitazone, metformin, and gliclazide. Furthermore, a recent model by de Gaetano *et al*.,^19^ including more complex dynamics of the number of β-cells, has been used to describe observations from the Diabetes Prevention Program Study.^20^

In this work, we have extended the T2DM progression model presented by de Gaetano *et al*.^19^ by including specific IL-1β effects on β-cell turnover and function based on available *ex vivo* observations. We have then used this extended model to simulate a 13-week treatment with anakinra and compared the simulation results with observed clinical effects.^5,6^ In addition to providing a new perspective on the possible mechanisms of IL-1β–blocking action in T2DM and its potential use in β-cell regeneration, our work presents a new case in point of how a quantitative systems pharmacology approach can be used to investigate clinical questions and translate preclinical data into clinically relevant insights.

## Results

**Figure 1:**
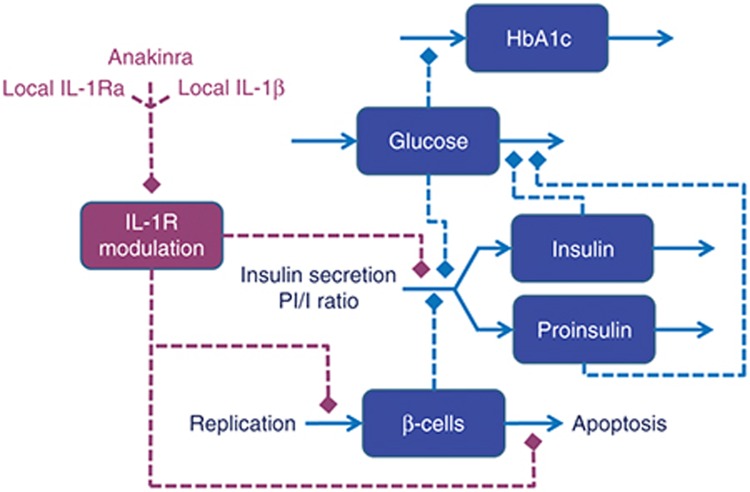
Model outline. Interleukin-1R (IL-1R) modulation, regulated by local IL-1β, IL-1Ra, and anakinra, affects β-cell apoptosis and replication, β-cell insulin secretion capacity, and the PI/I secretion ratio (red dashed lines). Glucose affects insulin and proinsulin secretion, the PI/I secretion ratio, and HbA1c production (blue dashed lines). Proinsulin and insulin affects glucose clearance (blue dashed lines). PI/I, proinsulin/insulin.

Our extended T2DM progression model is outlined in **Figure 1**. The model describes the long-term dynamics of glucose, insulin, proinsulin, HbA1c, and β-cell mass in response to modulation of the IL-1 receptor (IL-1R) by local IL-1β, IL-1Ra, and anakinra in the vicinity of the β-cells. In summary, the development of the model can be subdivided into four key parts: (i) the use of previously published models and parameters to describe fasting plasma glucose (FPG), insulin, and HbA1c dynamics^19,21,22^; (ii) the use of *ex vivo* data to develop and integrate new equations of the effect of IL-1β, endogenous IL-1 receptor antagonist (IL-1Ra), and anakinra on β-cell replication and apoptosis,^3,4^ β-cell insulin secretion capacity,^3,4^ and the conversion of proinsulin to insulin^23,24^; (iii) the definition of an initial diseased state using clinical parameters found in literature^5,25,26^; and (iv) the use of a pharmacokinetic model of anakinra and clinical data from Larsen *et al*.^5,6^ to describe local antagonist and IL-1β concentrations during and after anakinra treatment. A more detailed description of the model development process, model equations, and parameter values can be found in Methods section and in **Supplementary Information**.

### Summary of model characteristics

**Figure 2:**
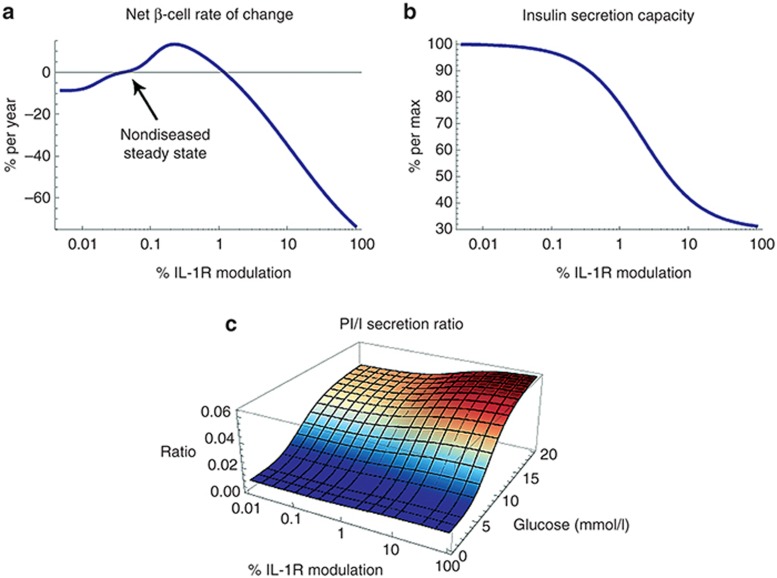
Relationships between level of interleukin-1R (IL-1R) modulation and (**a**) β-cell rate of change (replication–apoptosis), (**b**) insulin secretion capacity, and (**c**) PI/I secretion ratio. The PI/I secretion ratio is also affected by glucose. All relationships have been derived from *ex vivo* data (see **Supplementary Information**). PI/I, proinsulin/insulin.

The key characteristics of our model are summarized in **Figure 2**, which shows the explicit effects of IL-1R modulation on net β-cell rate of change (apoptosis–replication), β-cell insulin secretion capacity, and the PI/I secretion ratio. As can be seen in **Figure 2a**, the relationship between IL-1R modulation and net β-cell rate of change is bell-shaped, indicating that intermediate modulation will produce a net β-cell mass increase, while low and high modulation will have the opposite effect. The zero-crossings, implying constant β-cell mass, were identified based on the assumption of a nondiseased steady state and a basal β-cell turnover rate (**Supplementary Table S1** and **Supplementary Information**). Further, **Figure 2b** shows a monotonically decreasing β-cell insulin secretion capacity with increasing IL-1R modulation. Based on data from Maedler *et al*.,^3^ this relationship could possibly be bell-shaped, similar to that of β-cell rate of change. However, it has previously been shown by Spinas *et al*.^27^ that even though such a bell-shaped relationship can be seen during the first days of exposure to increasing concentrations of IL-1β, the stimulatory effects of intermediate concentrations on insulin secretion disappear after a prolonged exposure of 6 days. Admittedly, the experiment by Spinas *et al.*^27^ was performed using rat islets, as opposed to the use of human islets in Maedler *et al*.,^3^ but since the Maedler data only capture the relationship at 4 days, there is no way of knowing if the stimulatory effects seen in human islets are longer-lasting. Therefore, our model considers only an inhibitory effect of increasing IL-1R modulation on the β-cell insulin secretion capacity. Finally, the model accounts for both glucose and IL-1R effects on the PI/I secretion ratio (**Figure 2c**).

### Initial diseased state defined based on reported patient baseline values and an assumed decrease in β-cell mass

To configure the initial diseased state of our model, representing an average patient in Larsen *et al*.,^5,6^ we used baseline patient characteristics from Larsen *et al*.^5^ to set initial conditions for FPG, HbA1c, and plasma insulin (**Supplementary Table S1**). We then calculated what changes in disease variables would be required to move the system from a nondiseased state to the defined diseased state—assuming that these changes also include the effect of any previous standard of care treatment. While a change in insulin sensitivity (to 22% of normal) could be derived directly from baseline glucose and insulin levels, we used human autopsy data^25,26^ to define a plausible decrease in β-cell mass, which then allowed us to calculate the necessary changes in insulin secretion capacity, IL-1R modulation, and PI/I ratio. Notably, if assuming a 60% decrease in β-cell mass, the calculated increase in IL-1R modulation leads to a corresponding 2.3-fold increase in the rate of β-cell apoptosis and a PI/I ratio of ~0.43, which is in line with autopsy findings^26,28^ and the baseline PI/I value reported in Larsen *et al*.,^5^ respectively. Moreover, considering a limited change in local endogenous IL-Ra concentrations, the increase in IL-1R modulation would require a ~100-fold increase in local IL-1β concentrations, which is supported by *ex vivo* data showing that IL-1β mRNA expression can be increased >100-fold in β-cells of patients with T2DM.^2^

### Anakinra treatment assumed to result in rapid and near-complete suppression of local IL-1β

**Figure 3:**
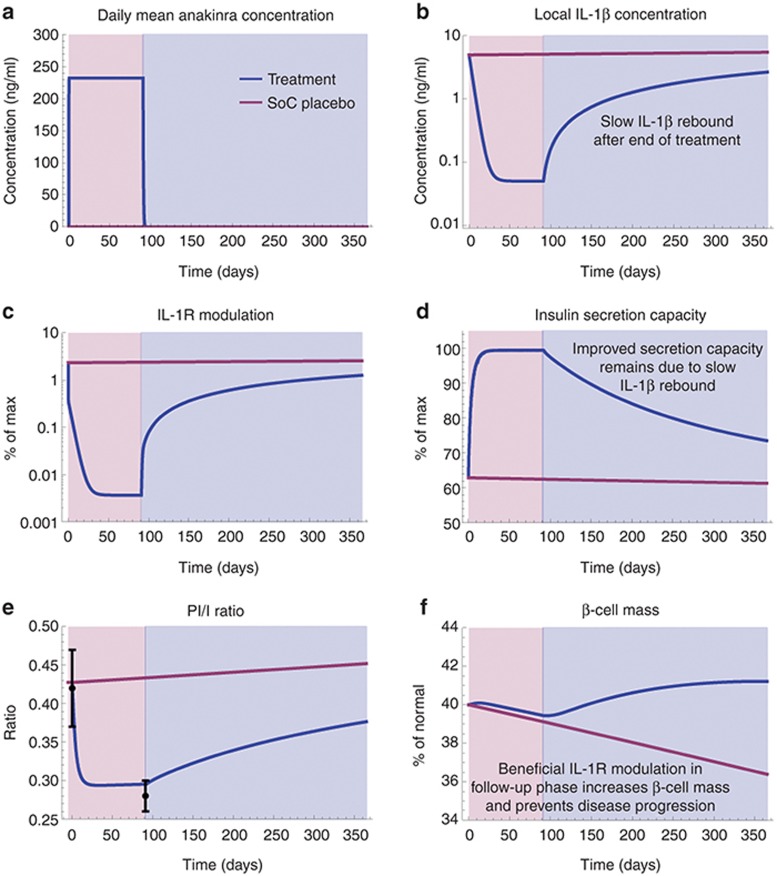
Predicted treatment effects (blue lines) vs. standard of care placebo disease progression (red lines) after 13 weeks of anakinra treatment (red area) and 39-week follow-up (blue area). (**a**) Mean daily anakinra concentration, (**b**) local interleukin-1β (IL-1β) concentration, (**c**) IL-1R modulation, (**d**) insulin secretion capacity, (**e**) PI/I ratio, and (**f**) β-cell mass. Black points with bars in **e** represent PI/I data (means ± SEM) for anakinra responders extracted from Larsen *et al*.^5,6^ PI/I, proinsulin/insulin; SoC, standard of care.

To describe the effect of anakinra treatment on β-cell function and turnover, we used a pharmacokinetic model of anakinra and assumed that anakinra levels at the β-cells are comparable to what has been measured in plasma^28^ (**Figure 3a**). We also considered possible effects of anakinra on local IL-1β concentrations during and after treatment. *Ex vivo* data have shown IL-1β mRNA expression in human β-cells to be positively affected by IL-1β itself,^2^ suggesting a self-stimulatory function of IL-1β. It is therefore probable that blocking IL-1 signaling will reduce the production, and hence the concentration, of local IL-1β. Predicting the exact level of this reduction *in vivo*—as well as what happens to the IL-1β concentration once blocking is stopped—is, however, difficult and becomes even more complicated considering the fact that the main source of local IL-1β might not only be the β cells themselves, but also infiltrating immune cells.^1^ To deal with this complexity, we (i) assumed that the level of anakinra dosing used in Larsen *et al*.^5^ is enough to bring local IL-1β levels back to a normal level, (ii) set the rate at which this level is reached based on a peripheral IL-1β clearance rate estimated from Lachmann *et al*.,^29^ and (iii) used reported values of IL-6, C-reactive protein, and the PI/I ratio^6^ to calibrate IL-1β dynamics after cessation of dosing, as well as in the placebo simulation (**Figure 3b** and **Supplementary Information**).

### Simulation shows that blocking IL-1 signaling improves β-cell function and mass

The simulated results of 13 weeks of anakinra 200 mg everyday treatment on IL-1R modulation, β-cell mass, β-cell insulin secretion capacity, and the PI/I ratio are shown in **Figure 3c**–**f** together with the predicted standard of care placebo disease progression, i.e., the disease progression to be expected with no change in previous standard of care treatment. The model predicts a rapid improvement in β-cell insulin secretion capacity and PI/I ratio (**Figure 3d**,**e**) as an effect of treatment. After cessation of dosing, this improvement then gradually declines toward the placebo state as a result of IL-1β increase (**Figure 3b**).

Furthermore, despite an initial β-cell decrease in the treatment phase, the model predicts a slight overall improvement in β-cell mass after 52 weeks (**Figure 3f**). This behavior is explained by the bell-shaped relationship between IL-1R modulation and net β-cell rate of change (**Figure 2a**). During treatment, IL-1R modulation is suppressed beyond the nondiseased steady state, leading to a negative β-cell rate of change. However, once dosing is ceased and the IL-1β concentration starts increasing back toward its diseased state, the system slowly passes through the region of beneficial IL-1R modulation, causing a net increase in β-cell mass.

### Model-predicted changes in FPG, HbA1c, and PI/I ratio agree with reported clinical data

**Figure 4:**
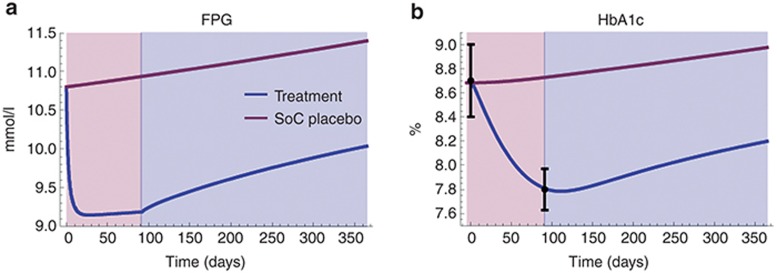
Predicted FPG (**a**) and HbA1c (**b**) treatment effects (blue lines) vs. placebo disease progression (red lines) after 13 weeks of anakinra treatment (red area) and 39-week follow-up (blue area). Black points with bars in **b** represent HbA1c data (means ± SEM) for anakinra responders extracted from Larsen *et al*.^5,6^ FPG, fasting plasma glucose; SoC, standard of care.

The simulated effects of changes in β-cell mass and function on FPG and HbA1c are presented in **Figure 4** and show a drop of ~1.7 mmol/l and ~0.89% in FPG and HbA1c, respectively, after 13 weeks of treatment. Even though this is an overestimation compared with the average values reported in Larsen *et al*.,^5^ the HbA1c drop of ~0.89% (**Figure 4b**) agrees well with what is observed in the group of patients responding to anakinra treatment (~0.90%). This is true also for the predicted drop in the PI/I ratio (~0.13, **Figure 3e**), which is in close agreement with the reported absolute change for anakinra-responding patients (~0.14).

Due to changes in standard of care treatment in the follow-up phase in Larsen *et al*.,^5,6^ no direct comparison could be made between simulation results and reported HbA1c values at 52 weeks. However, to further relate the model to clinical data, we also studied the simulated placebo disease progression. **Figure 4** shows that the 1-year increase in FPG and HbA1c are predicted to be ~0.59 mmol/l and ~0.29%, respectively. Comparing these to what is reported in long-term diabetes studies,^18,30,31,32^ we note that the predicted changes are in line with observed values (0.2–1 mmol/l/year and ~0.2%/year).

### Improved β-cell function is the main contributor to improved glycemic control seen in the first year after treatment with anakinra

**Figure 5:**
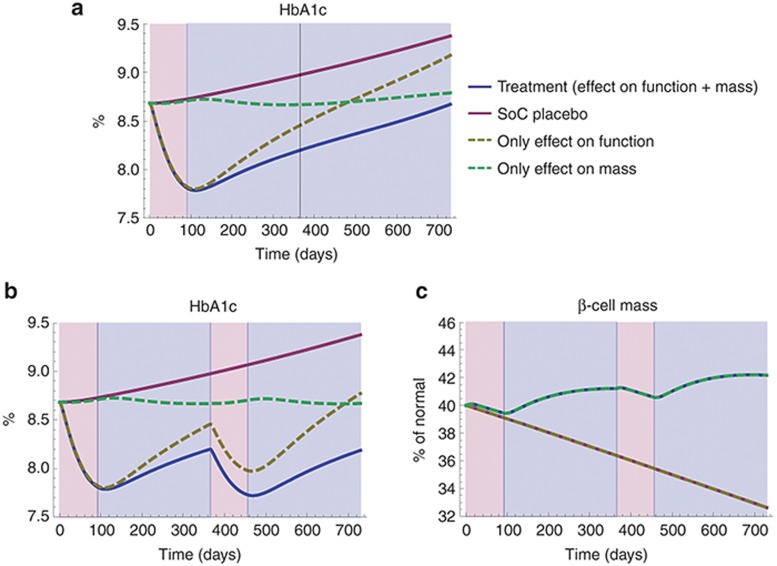
(**a**) Individual contributions of improved β-cell function (dashed yellow line) and β-cell mass (dashed green line) to the predicted improvement in HbA1c (blue line) during an extended period of 2 years, assuming interleukin-1β(IL-1β) continues to return back toward its untreated (placebo) state. (**b**,**c**) Predicted improvement in HbA1c and β-cell mass for a repeated anakinra treatment after 1 year (insulin sensitivity assumed constant). Please note that the trajectories of the effect of improved β-cell function and placebo, as well as the effect of improved β-cell mass and treatment, are overlapping in **c**. SoC, standard of care.

Taking in mind the disparate dynamics of improved β-cell function and mass seen in **Figure 3d**–**f**, it is possible to conclude that improved β-cell function is the main reason for the predicted improvement in FPG and HbA1c seen after 13 weeks of treatment (**Figure 4**). In fact, simulating the effect of improved β-cell function and mass on HbA1c separately shows that improved β-cell function is not only the reason for the short-term improvement (<13 weeks) but also has a significant impact on HbA1c 39 weeks after the end of treatment (**Figure 5a**). Despite the β-cell mass increase (**Figure 3f**), only a minor effect on HbA1c (<0.2%)—likely of little clinical significance—is predicted to be due to the improved β-cell mass at this point. On the other hand—considering a longer term perspective—of note is that the improvement in β-cell mass is predicted to lead to a sustained offset in HbA1c compared with placebo (**Figure 5a**), even though IL-1β, and thus β-cell function, would eventually return to their respective untreated states. Furthermore, seeing that there is a maximum limit to how much β-cell function can be increased by blocking IL-1β (**Figure 2b**), it is only an increasing β-cell mass that could lead to both a long-term and continued improvement in glycemia. This is illustrated in **Figure 5b**,**c**, which show the hypothetical scenario of repeating the anakinra treatment after 1 year. With an effect on both β-cell mass and function, we would expect to see further improvements in glycemia after the second treatment, while no such improvement would be seen if the effect on β-cell mass was absent (**Figure 5b**).

## Discussion

Mathematical modeling is emerging as a valuable tool to understand the pathophysiology of complex multietiological diseases such as T2DM and is proving helpful in designing better therapies. We report the development of a systems pharmacology model, based on previously published models of T2DM disease progression and literature-reported results on the impact of IL-1β and IL-1Ra on β-cell mass and function, which accounts for the effect of the inflammatory IL-1 pathway. We have used the model to understand the effect of anti-IL-1β therapies—especially that of anakinra—in T2DM patients.

Simulations with the model indicate that an improvement in β-cell function—caused by the combined effect of IL-1β blocking and a delayed return of local IL-1β—is the main reason for the observed efficacy and its sustenance after cessation of anakinra dosing reported in Larsen *et al*.^5,6^ Even though this has been hypothesized before,^1,2^ our model is the first instance of quantitative evidence to support this hypothesis. Importantly, the dynamics of local IL-1β is imperative to describing the treatment and follow-up response using our model. We have currently based these dynamics on relatively uncertain assumptions, such as assuming near-complete suppression of IL-1β production at the β-cells during treatment and using an *ex vivo* defined relationship between IL-1R modulation and the PI/I secretion ratio, as well as sparse IL-6, C-reactive protein, and PI/I data reported in Larsen *et al*.,^6^ to describe local IL-1β concentrations in the follow-up phase. However, literature evidence supports a self-stimulatory role for IL-1β^2^ indicating that return to baseline IL-1β levels after sustained suppression are likely to be delayed. A sensitivity analysis (not shown) also revealed that in the absence of sustained IL-1β reduction, the observed effects would not be predicted by the model.

In addition to a significant and sustained improvement in β-cell function, model simulations also predict that treatment with anakinra may lead to an increase in β-cell mass, but that this effect is likely to result in improvements in clinically relevant disease parameters, such as HbA1c, only after >1 years. If this is true, a number of key implications can be drawn regarding the therapeutic benefit of anakinra in T2DM. First, short-term treatment is unlikely to result in benefits on β-cell mass. Instead, longer treatment—in the order of many years—is required to regenerate β-cells in T2DM patients. Furthermore, the model suggests that treatment with IL-1β therapies may have a complex dose–response relationship—i.e., an optimal dose exists which maximizes benefit. A more quantitative model, which takes into account other features of the system such as IL-1β self-stimulation, IL-1β tissue levels, etc., are required to accurately predict that relationship. Finally, anakinra therapy alone is unlikely to provide the desired clinical management results in this population in the short-term (assuming an HbA1c treatment target of 6.5%). Combination with another symptomatic therapy could be considered for short-term and longer term beneficial therapeutic effects. More detailed calibration of the model using data on symptomatic therapy (e.g., Hardy *et al*.^20^) is required for the design of such combination therapies.

The largest uncertainty with our model is the fact that the IL-1β parameters are based mostly on experiments from *ex vivo* human pancreatic β-cells and that the translation of the results to the human *in vivo* condition is not known. For instance, to convert the *ex vivo* effect of IL-1R modulation on β-cell replication and apoptosis, we had to consider the relative changes in these processes reported in Maedler *et al*.^3,4^ on top of an assumed basal *in vivo* β-cell lifespan (**Supplementary Information**). Besides, the *ex vivo* data provide only short-term snapshots of IL-1β effects on β-cell function and mass and do not tell us if these effects actually are persistent. Therefore, the regenerative effects of intermediate IL-1R modulation on β-cell mass may not be present *in vivo* when considering a time frame of many years or may be present only when moving from low-to-high IL-1R modulation and not when returning from a sustained period of high modulation. In fact, the nonclinical–clinical translation of results has been a limiting factor in β-cell research in general—e.g., the GLP1 agonist exenatide was shown to cause β-cell regeneration *in vitro* and in rat models^33^ but was recently shown to not cause any β-cell regeneration in humans.^34^ The absence of data on therapies known to cause β-cell changes in T2DM patients limits our ability to apply anything but the simplest level of interpretation to model predictions. Nevertheless, our model provides a starting point to understand concurrence and dissonance in the nonclinical–clinical translation of results for IL-1β therapies. A detailed, model-based, biomarker analysis of preclinical data, similar to that in development for arthritis models,^35,36^ is required to further investigate these questions and attempt to bridge the preclinical–clinical translation.

As with all mathematical representations of complex systems, our model also contains substantial simplifications of the system of interest. For example, we implicitly assume that glucotoxicity is exerted on the insulin secretion capacity and turnover of β-cells only through contributing to increased IL-1β levels^4^ and thus increased IL-1R modulation. We have also ignored the effects of IL-1β in peripheral tissues, where it may impact insulin sensitivity,^37^ mainly because no such effects were seen in the studies of Larsen *et al*.^5,6^ Despite these simplifications, however, our model still manages to provide a surprisingly accurate description of clinical observations. Also, even though additional factors may improve the overall representation of the physiological system, the results presented above are still valid from a qualitative point of view. For example, because we have assumed that glucotoxicity is exerted only through IL-1β in our model, this presents the best efficacy scenario for an anti-IL-1β therapy on β-cell regeneration and function. Moreover, the lack of representation of the peripheral effects of anakinra or the dynamics of local IL-1β are likely to impact the quantitative prediction of HbA1c effects but unlikely to alter our results on the lack of β-cell regeneration effects in Larsen *et al*.^5,6^

In conclusion, our work suggests that the improved glycemia seen after 13 weeks treatment with anakinra^5,6^ is most likely due to improved β-cell function rather than an increase in β-cell mass, that a sustained low concentration of IL-1β is a prerequisite for a sustained treatment response, and that a treatment effect on β-cell mass most probably requires years before having a significant clinical impact. In addition, our developed model provides a framework to further link *ex vivo*/*in vitro* and *in vivo* data on the role of IL-1β in T2DM and to help in the design of new clinical trials required to demonstrate impact on β-cell mass.

## Methods

The extended T2DM disease progression model was developed and simulated as a system of differential-algebraic equations using Mathematica (version 9.0 by Wolfram Research, Champaign, IL) (**Supplementary Notepad**). The model development process was carried out as follows: First, the feedback relationship between insulin and glucose (see model outline in **Figure 1**) was defined using equations and parameters previously published by de Gaetano *et al.*^19^ (**Supplementary Information Equations 1,2**), while a model proposed in Hamrén *et al*.,^21^ and extended in Lledó-García *et al*.,^22^ was used to relate glucose levels to levels of HbA1C. Second, the IL-1R modulation effects described in **Figure 2** were modeled using phenomenological equations (**Supplementary Information Equations 3–9**) derived from *ex vivo* data of the relative effects of IL-1β and IL-1Ra on β-cell replication, apoptosis (**Supplementary Figure S1**),^3,4^ and β-cell insulin secretion capacity,^3,4^ as well as IL-1β and glucose effects on the conversion of proinsulin to insulin.^23,24^ These *ex vivo* relationships were then scaled and integrated into the glucose–insulin model by considering a nondiseased steady state (**Supplementary Table S1**). Finally, local anakinra (**Supplementary Information Equations 10,11**) and IL-1β (**Supplementary Information Equations 12,13**) concentrations were modeled as described in the Results (section “Anakinra treatment assumed to result in rapid and near-complete suppression of local IL-1β”), while local endogenous IL-1Ra levels (**Supplementary Information Equation 14**) were assumed constant. Please note that no whole-model parameter estimation was performed; the different parts were either directly obtained from literature or piecewise developed and then assembled to create the complete model.

For more details, the derivation of all model equations and parameters, as well as all assumptions made, can be found thoroughly presented in the **Supplementary Information** and **Supplementary Tables S1 and S2**.

## Conflict of Interest

R.P. and E.N. from Wolfram MathCore AB were paid consultants of MedImmune, B.A. and M.P. are employed at MedImmune, and A.M. is employed at AstraZeneca. G.C. declared no conflict of interest.

## Author Contributions

R.P., E.N., G.C., and B.A. designed the research. R.P. and E.N. performed the research. R.P., E.N., and B.A. wrote the manuscript.

## Study Highlights


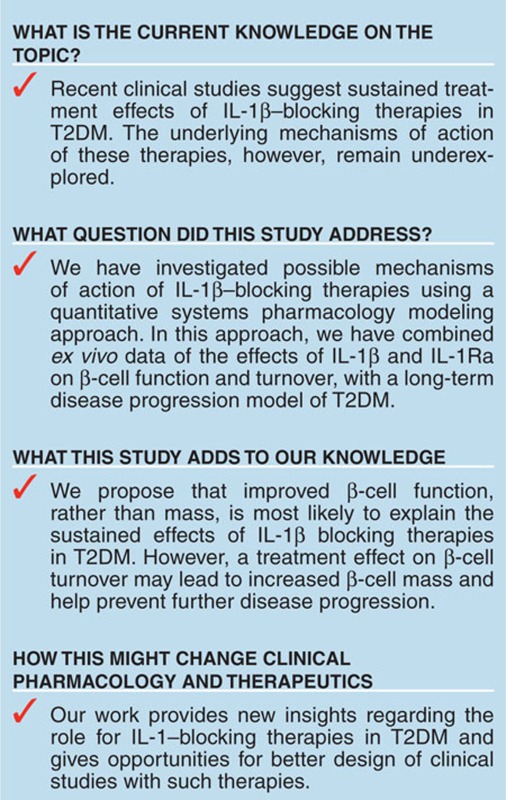


## Supplementary Material

Supplementary Information

Supplementary Notepad
