# Supplementary material for: Effects of IL-1β–Blocking Therapies in Type 2 Diabetes Mellitus: A Quantitative Systems Pharmacology Modeling Approach to Explore Underlying Mechanisms
Source: CPT Pharmacometrics Syst Pharmacol. 2014 Jun 11;3(6):e118–. doi: 10.1038/psp.2014.16 (PMC4076803; doi:10.1038/psp.2014.16)
Supplement: Supplementary Figure S1 [file psp201416x1.doc]

Supplementary Figure 1. Parameter fit to experimental data.


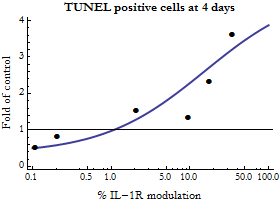


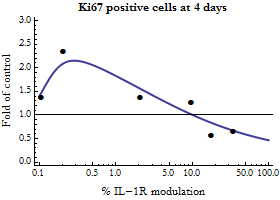


Data were obtained from human islets cultured for 4 days using different concentrations of IL-1β. IL-1β concentrations were translated to IL-1R modulation levels using Suppl. Eq. 4 and concentrations of endogenously produced IL-1Ra reported in[^1^](#_ENREF_1). TUNEL and Ki67 positive cells reflects β-cell apoptosis and replication rates respectively.

1. Maedler K, Schumann DM, Sauter N, Ellingsgaard H, Bosco D, Baertschiger R*, et al.* Low concentration of interleukin-1beta induces FLICE-inhibitory protein-mediated beta-cell proliferation in human pancreatic islets. *Diabetes* 2006, **55**(10)**:** 2713-2722.

2. Maedler K, Sergeev P, Ris F, Oberholzer J, Joller-Jemelka HI, Spinas GA*, et al.* Glucose-induced beta cell production of IL-1beta contributes to glucotoxicity in human pancreatic islets. *J Clin Invest* 2002, **110**(6)**:** 851-860.
