# Supplementary material for: Effects of IL-1β–Blocking Therapies in Type 2 Diabetes Mellitus: A Quantitative Systems Pharmacology Modeling Approach to Explore Underlying Mechanisms
Source: CPT Pharmacometrics Syst Pharmacol. 2014 Jun 11;3(6):e118–. doi: 10.1038/psp.2014.16 (PMC4076803; doi:10.1038/psp.2014.16)
Supplement: Supplementary Tables S1 [file psp201416x2.doc]

Supplementary Table 1. Non-diseased and diseased states

|  | **Non-diseased**  **(steady state)** | **Diseased** |
| --- | --- | --- |
| Glucose (mM) | 5.0 | 10.8 |
| Insulin (pM) | 50 | 100 |
| Proinsulin (pM) | 6.5 | 43 |
| HbA1c (%) | 5.0 | 8.7 |
| Insulin sensitivity (% of normal) | 100 | 22 |
| β-cell mass (% of normal) | 100 | 40 |
| Insulin secretion capacity  (% of normal) | 100 | 64 |
| Local IL-1β (ng/ml) | 0.05 | 5 |
| Local IL-1Ra (ng/ml) | 25 | 40 |
