# Supplementary material for: Effects of IL-1β–Blocking Therapies in Type 2 Diabetes Mellitus: A Quantitative Systems Pharmacology Modeling Approach to Explore Underlying Mechanisms
Source: CPT Pharmacometrics Syst Pharmacol. 2014 Jun 11;3(6):e118–. doi: 10.1038/psp.2014.16 (PMC4076803; doi:10.1038/psp.2014.16)
Supplement: Supplementary Tables S2 [file psp201416x3.doc]

Supplementary Table 2. Model parameters

|  | **Name** | **Value (unit)** | **References** |
| --- | --- | --- | --- |
| Insulin and glucose | Tgl | 0.0254 (mM min^-1^) | From steady-state |
|  | Kxg | 0.000016 (min^-1^) | From literature[^1^](#_ENREF_1) |
|  | Gh | 9 (mM) | From literature[^2^](#_ENREF_2) |
|  | vh | 4 |  |
|  | Kxgi(, non-diseased) | 0.0001 (pM^-1^ min^-1^) |  |
|  | Kxgi (diseased) | 0.0000224 (pM^-1^ min^-1^) |  |
|  | Kxi | 0.05 (min^-1^) |  |
| Insulin secretion capacity | ks | 0.291 (pM min^-1^ (% β-cells)^-1^) | From steady-state |
|  | vs | 0.7 | Estimated |
|  | kms | 0.021 | Estimated |
|  | τs | 0.5 (day^-1^) | Estimated[^6^](#_ENREF_6) |
| β-cell rate of change | kr | 0.000376 (day^-1^) | From steady-state and |
|  | vlr | 1.8 | Fitted to data |
|  | kmlr | 0.0011 |  |
|  | xlr | 3 |  |
|  | vhr | 2.7 |  |
|  | kmhr | 0.018 |  |
|  | xhr | 0.5 |  |
|  | ka | 0.000552 (day^-1^) | From steady-state and |
|  | vla | 0.65 | Fitted to data |
|  | kmla | 0.00018 |  |
|  | xla | 3 |  |
|  | vha | 4.6 |  |
|  | kmha | 0.155 |  |
|  | xha | 0.66 |  |
| Proinsulin secretion fraction | kf | 0.00958 | From steady-state |
|  | vf | 0.4 | Estimated[^10^](#_ENREF_10) |
|  | kmf | 0.021 | Assumed equal to kms |
|  | vfg | 4 | Estimated[^11^](#_ENREF_11) |
|  | kmfg | 9 (mM) | Assumed equal to Gh |
|  | xfg | 4 | Assumed equal to vh |
|  | τf | 0.5 (day^-1^) | Assumed equal to τs |
| IL-1R modulation | kmi | 8.5 (ng ml^-1^) | From literature[^12^](#_ENREF_12) |
|  | kii | 1.7 (ng ml^-1^) | From literature[^12^](#_ENREF_12) |
| Anakinra PK | kab | 3.94 (day^-1^) | Estimated |
|  | CL | 432 (l day^-1^) |  |
|  | Vp | 48 (l) |  |
| IL-1β dynamics | kplacebo | 0.00137 (ng ml^-1^ day^-1^) | Estimated |
|  | k1 | 0.2 (day^-1^) | Estimated[^15^](#_ENREF_15) |
|  | k2 | 0.0025 (day^-1^) | Estimated |
|  | IL1bH | - 1. (ng ml^-1^) | Estimated |

1. Topp B, Promislow K, deVries G, Miura RM, Finegood DT. A model of beta-cell mass, insulin, and glucose kinetics: pathways to diabetes. *J Theor Biol* 2000, **206**(4)**:** 605-619.

2. De Gaetano A, Hardy T, Beck B, Abu-Raddad E, Palumbo P, Bue-Valleskey J*, et al.* Mathematical models of diabetes progression. *Am J Physiol Endocrinol Metab* 2008, **295**(6)**:** E1462-1479.

3. Maedler K, Schumann DM, Sauter N, Ellingsgaard H, Bosco D, Baertschiger R*, et al.* Low concentration of interleukin-1beta induces FLICE-inhibitory protein-mediated beta-cell proliferation in human pancreatic islets. *Diabetes* 2006, **55**(10)**:** 2713-2722.

4. Maedler K, Sergeev P, Ris F, Oberholzer J, Joller-Jemelka HI, Spinas GA*, et al.* Glucose-induced beta cell production of IL-1beta contributes to glucotoxicity in human pancreatic islets. *J Clin Invest* 2002, **110**(6)**:** 851-860.

5. Yang BB, Baughman S, Sullivan JT. Pharmacokinetics of anakinra in subjects with different levels of renal function. *Clin Pharmacol Ther* 2003, **74**(1)**:** 85-94.

6. Chang DM, Chang SY, Yeh MK, Lai JH. The pharmacokinetics of interleukin-1 receptor antagonist in Chinese subjects with rheumatoid arthritis. *Pharmacol Res* 2004, **50**(3)**:** 371-376.

7. Spinas GA, Palmer JP, Mandrup-Poulsen T, Andersen H, Nielsen JH, Nerup J. The bimodal effect of interleukin 1 on rat pancreatic beta-cells--stimulation followed by inhibition--depends upon dose, duration of exposure, and ambient glucose concentration. *Acta Endocrinol (Copenh)* 1988, **119**(2)**:** 307-311.

8. Perl S, Kushner JA, Buchholz BA, Meeker AK, Stein GM, Hsieh M*, et al.* Significant human beta-cell turnover is limited to the first three decades of life as determined by in vivo thymidine analog incorporation and radiocarbon dating. *J Clin Endocrinol Metab* 2010, **95**(10)**:** E234-239.

9. Butler AE, Janson J, Bonner-Weir S, Ritzel R, Rizza RA, Butler PC. Beta-cell deficit and increased beta-cell apoptosis in humans with type 2 diabetes. *Diabetes* 2003, **52**(1)**:** 102-110.

10. Hostens K, Pavlovic D, Zambre Y, Ling Z, Van Schravendijk C, Eizirik DL*, et al.* Exposure of human islets to cytokines can result in disproportionately elevated proinsulin release. *J Clin Invest* 1999, **104**(1)**:** 67-72.

11. Borjesson A, Carlsson C. Altered proinsulin conversion in rat pancreatic islets exposed long-term to various glucose concentrations or interleukin-1beta. *J Endocrinol* 2007, **192**(2)**:** 381-387.

12. Dinarello CA. Biologic basis for interleukin-1 in disease. *Blood* 1996, **87**(6)**:** 2095-2147.

13. Larsen CM, Faulenbach M, Vaag A, Volund A, Ehses JA, Seifert B*, et al.* Interleukin-1-receptor antagonist in type 2 diabetes mellitus. *N Engl J Med* 2007, **356**(15)**:** 1517-1526.

14. Larsen CM, Faulenbach M, Vaag A, Ehses JA, Donath MY, Mandrup-Poulsen T. Sustained effects of interleukin-1 receptor antagonist treatment in type 2 diabetes. *Diabetes Care* 2009, **32**(9)**:** 1663-1668.

15. Lachmann HJ, Lowe P, Felix SD, Rordorf C, Leslie K, Madhoo S*, et al.* In vivo regulation of interleukin 1beta in patients with cryopyrin-associated periodic syndromes. *J Exp Med* 2009, **206**(5)**:** 1029-1036.

16. Spranger J, Kroke A, Mohlig M, Hoffmann K, Bergmann MM, Ristow M*, et al.* Inflammatory cytokines and the risk to develop type 2 diabetes: results of the prospective population-based European Prospective Investigation into Cancer and Nutrition (EPIC)-Potsdam Study. *Diabetes* 2003, **52**(3)**:** 812-817.
