# Supplementary material for: Effects of IL-1β–Blocking Therapies in Type 2 Diabetes Mellitus: A Quantitative Systems Pharmacology Modeling Approach to Explore Underlying Mechanisms
Source: CPT Pharmacometrics Syst Pharmacol. 2014 Jun 11;3(6):e118–. doi: 10.1038/psp.2014.16 (PMC4076803; doi:10.1038/psp.2014.16)
Supplement: Supplementary Information [file psp201416x4.pdf]

Supplementary Information

Effects of IL-1β Blocking Therapies in Type 2 Diabetes Mellitus: A Quantitative Systems Pharmacology Modeling Approach to Explore Underlying Mechanisms

Robert Palmér, Elin Nyman, Mark Penney, Anna Marley, Gunnar Cedersund, and Balaji Agoram

This Supplementary information contains a step-by-step description of the development of our type 2 diabetes and IL-1β disease progression model, as well as all model equations, assumptions, and the non-diseased and diseased states used in our study. All parameter values can be found summarized in Suppl. Table 2. For fitted equations, the goodness of fit is shown in Suppl. Figure 1.

Derivation of model equations and parameters

Step 1 – Use of previously published models

As a first step in the development of our model, we used previously published equations to describe the interrelationships between glucose, insulin, and HbA1c. We also extended these equations to include proinsulin dynamics and effects.

Insulin and glucose. Both plasma insulin and plasma glucose dynamics were modeled based on equations and parameters used by de Gaetano *et al.*[1], with the addition of an extra effect of proinsulin on glucose uptake:

$$Insulin(t) = \frac{B(t) \cdot TigB(t)}{Kxi} \cdot \frac{\left(\frac{Glucose(t)}{Gh}\right)^{vh}}{1 + \left(\frac{Glucose(t)}{Gh}\right)^{vh}} \tag{1}$$

$$Glucose(t) = \frac{Tgl}{Kxg + Kxgi \cdot (Insulin(t) + 0.1 \cdot Proinsulin(t))} \tag{2}$$

Here, *B* is β-cell mass, *TigB* is the maximum insulin secretion capacity per unit β-cell, *Kxi* is the insulin clearance rate, and *Gh* and *vh* define a Hill-shaped relationship between insulin secretion and glucose. Furthermore, *Tgl* describes basal insulin-independent glucose output, *Kxg* insulin-independent glucose uptake, and *Kxgi* insulin sensitivity.

While values for *Kxi*, *Ghi*, *vh*¸ and *Kxgi* were taken from[1], *Tgl* was calculated from steady-state conditions (Suppl. Table 1) and *Kxg* was set according to the value reported in[2]. Moreover, proinsulin was assumed to have 10 % the biologic activity of insulin[3].

HbA1C. The relationship between glucose and HbA1C was described using a semi-mechanistic model proposed by Hamrén *et al.*[4], and later extended in[5]. The model accounts for red blood cell (RBC) life-span using a 12 transit compartment model and includes a glucose-dependent glycosylation rate. Unlike Hamrén *et al.*[4], who estimated a RBC life-span of 135 days, we considered a shorter life-span of 100 days to be more plausible[5, 6]. For a full description of the model equations, see the original publications[4, 5].

Proinsulin. Proinsulin levels were modeled assuming proinsulin secretion to be proportional to insulin secretion and proinsulin clearance to be 10% of that of insulin[7]:

$$Proinsulin(t) = f(t) \cdot \frac{B(t) \cdot TigB}{0.1 \cdot Kxi} \cdot \frac{\left(\frac{Glucose(t)}{Gh}\right)^{vh}}{1 + \left(\frac{Glucose(t)}{Gh}\right)^{vh}} \tag{3}$$

Step 2 – Extending the model with *ex vivo* IL-1β and IL-1Ra effects

In the second stage of model development, we extended the above equations by incorporating IL-1β and IL-1Ra effects, through modulation of the IL-1 receptor, on the maximum insulin secretion capacity (*TigB*), β-cell mass (*B*), and the proinsulin-insulin secretion factor (*f*). These effects are illustrated in Figure 2 in the main text. The subfigures were produced by plotting Equation 8 minus Equation 7 (Figure 2A), Equation 5 (Figure 2B), and Equation 9 (Figure 2C). Steady state was assumed for Equation 5 and 9 when plotting. The equations were derived as follows:

IL-1R modulation. IL-1R modulation was modeled assuming competitive inhibition between IL-1β and IL-1Ra /anakinra:

$$IL1R(t) = \frac{IL1b(t)}{kmi \cdot \left(1 + \frac{IL1Ra(t) + Anakinra(t)}{kii}\right) + IL1b(t)} \tag{4}$$

The affinities for IL-1β and IL-1Ra/anakinra were set according to[8].

Insulin secretion capacity. The insulin secretion capacity per unit β-cells was modeled as a function of IL-1R modulation based on data from human[9, 10] and rodent[11] islets:

$$\frac{dTigB(t)}{dt} = \tau s \cdot \left(ks \cdot \left(1 - \frac{vs \cdot IL1R(t)}{kms + IL1R(t)}\right) - TigB(t)\right) \tag{5}$$

Studying the data from human islets, one could speculate that the relationship between IL-1R modulation and insulin secretion should be bell-shaped, and that intermediate receptor activity actually has a

stimulatory effect on insulin secretion. However, it has previously been proposed that such a stimulatory effect may in fact be time-dependent and disappear after prolonged stimulation of IL-1 signaling[11], an aspect not investigated in[9]. Therefore, we only considered an inhibitory effect of IL-1R modulation on the insulin secretion capacity.

Due to uncertainties and lack of information in the data, making anything but the simplest assumptions regarding the parameters *vs* and *kms* is difficult. In two different publications[9, 10], glucose stimulated insulin secretion was shown to be reduced by 60-70 % after exposure to high IL-1β and we thus considered a corresponding value for *vs*. Furthermore, to estimate *kms*, we ignored the stimulatory effects reported for intermediate IL-1β concentrations[9] and assumed the half-maximum negative response to be reached at an IL-1β concentration of ~100-200 pg/ml. We then translated this concentration to a corresponding value for IL-1R modulation by considering a concentration of ~140 pg/ml of endogenously produced IL-1Ra reported for the same experimental conditions and using Equation 4:

$$kms = \frac{0.2}{kmi \cdot \left(1 + \frac{0.14)}{kii}\right) + 0.2}$$

Finally, the scaling factor *ks* was calculated from steady-state conditions (Suppl. Table 1) and *τs* was set to 0.5 day^{-1} to capture the fact that IL-1 effects are not reached instantly, but after a few days.

β-cell rate of change. β-cell rate of change was modeled as a difference between β-cell apoptosis and replication. Any possible contribution of neogenesis to β-cell regeneration was assumed to be small[12, 13] and neglected. Both the rate of apoptosis and replication were considered to be functions of IL-1R modulation, also based on data from[9, 10]. This time, however, both beneficial and negative effects were considered, mainly because no study investigating more long-term effects could be found.

$$\frac{dB(t)}{dt} = (replication - apoptosis) \cdot B \tag{6}$$

$$apoptosis = ka \cdot \left(1 - \frac{vla \cdot IL1R(t)^{xla}}{kmla^{xla} + IL1R(t)^{xla}} + \frac{vha \cdot IL1R(t)^{xha}}{kmha^{xha} + IL1R^{xha}}\right) \tag{7}$$

$$replication = kr \cdot \left(1 + \frac{vlr \cdot IL1R(t)^{xlr}}{kmlr^{xlr} + IL1R(t)^{xlr}} - \frac{vhr \cdot IL1R(t)^{xhr}}{kmhr^{xhr} + IL1R(t)^{xhr}}\right) \tag{8}$$

All parameters except *ka* and *kr* were fitted to reported mean relative changes in apoptosis and replication rates after 4 days exposure to various IL-1β concentrations[9]. The goodness of fit is shown in Suppl. Figure 1. After fitting the parameters, *ka* and *kr* were calculated from non-diseased steady state conditions (Suppl. Table 1) and considering a basal β-cell turnover of 10 % per year. Even though the turnover and life-span of human β-cell is a question for debate and a wide range of values can be derived from current data[14, 15, 16, 17, 18], recent evidence points towards a very low or almost non-existent β-cell turnover in human adults. For instance, Perl *et al.*[15] reported an annual turnover of 25-60 % in two young adults,

while no turnover at all was observed in older subjects. Moreover, using the same reasoning as in[17] and assuming that (i) a 60 % loss in β-cell mass takes about 5 years to evolve and (ii) that this is a result of a 2-3 fold increase in β-cell apoptosis and a limited change in replication, this would imply a normal annual turnover of 6-9 %. Our assumption of a 10 % basal turnover should thus be in the range of plausible rates.

Proinsulin secretion fraction. The ratio of proinsulin to insulin secretion has been shown to be dependent on both glycemia and IL-1 signaling. While hyperglycemia seems to alter the ratio mainly through an increased β-cell secretory demand, however, increased IL-1 signaling acts by inhibiting the conversion of proinsulin into insulin, suggesting two different mechanisms[19, 20, 21]. We therefore considered a combined effect of glucose and IL-1R modulation on the proinsulin secretion factor:

$$\frac{df(t)}{dt} = \tau f \cdot \left(kf \cdot \left(1 + \frac{vf \cdot IL1R(t)}{kmf + IL1R(t)}\right) \cdot \left(1 + \frac{vfg \cdot glucose(t)^{xfg}}{kmfg^{xfg} + glucose(t)^{xfg}}\right) - f(t)\right) \tag{9}$$

The value for *vfg* was derived using data from[19], while *kmfg* and *xfg* were set equal to *Gh* and *vh* – assuming the effect of glycemia on the secretion factor to be directly related to insulin demand. Furthermore, *vf* was set based on data from human islets showing that high concentrations of IL-1β, in combination with IFN-γ, led to a 1.4-fold increase in the conversion time of proinsulin into insulin[20] and assuming that this leads to a similar effect on the secretion ratio. Finally, *kmf* and *τf* was assumed to be equal to *kms* and *τs* respectively, and *kf* was calculated from steady-state conditions (Suppl. Table 1).

Step 3 – Defining local IL-1β, IL-1Ra, and anakinra concentrations to simulate anakinra treatment

In the third and final step of model development, we first of all defined an initial state – to represent an average patient in[24, 25] – for our simulations (see section “Non-diseased and diseased states” below). To simulate treatment and placebo responses, we then defined concentration profiles for local IL-1β, IL-1Ra, and anakinra.

Anakinra pharmacokinetics. The anakinra plasma concentration was described by a one-compartment PK model with a subcutaneous administration site and with parameters estimated previously using data from[22, 23]. The local anakinra concentration at the β-cell was then assumed to be equal to the plasma concentration.

$$\frac{dAnakinrasc(t)}{dt} = -kab \cdot Anakinrasc(t) \tag{10}$$

$$\frac{dAnakinra(t)}{dt} = kab \cdot \frac{Anakinrasc(t)}{Vp} - \frac{CL}{Vp} \cdot Anakinra(t) \tag{11}$$

IL-1β. Local IL-1β was described using different equations for the treatment and placebo simulation. In the treatment simulation, local IL-1β was set to decrease to its assumed non-diseased level (*IL1bH*) during treatment and thereafter increase back towards its placebo state (Equation 13):

$$\frac{dIL1b(t)}{dt} = \begin{cases} k1 \cdot \left(IL1bH - IL1b(t)\right), & t < 91 \\ k2 \cdot \left(IL1b(0) + kplacebo \cdot t - IL1b(t)\right), & t > 91 \end{cases} \tag{12}$$

The rate of IL-1β decrease in the treatment phase, and thus the value of *k1*, was set based on a peripheral IL-1β clearance rate estimated from[24]. However, the exact value of the parameter has no significant impact on our conclusions as long as the non-diseased level is reached within a couple of weeks. Thereafter, the rate of IL-1β increase (*k2*) was estimated based on values of IL-6, CRP, PI/I ratio reported in[25, 26]. Admittedly, Larsen *et al*.[25] speculate that, while being surrogate markers for systemic IL-1 activity, IL-6 and CRP might not reflect IL-1 activity in the vicinity of the β-cells. However, the reported levels of CRP and IL-6 do demonstrate that 13 weeks of IL-1 blockade is capable of inducing a significant and partly sustained inflammatory remission, which at least suggests that there might be a sustained reduction also in local IL-1β. Such a sustained reduction in IL-1β is also supported by the partly sustained reduction seen in the PI/I ratio[25]. Therefore, we used a moderate value for *k2*, giving us an IL-1β level at 52 weeks of approximately half of its original value.

Using the same reasoning as above, IL-1β in the placebo simulation was assumed to increase slowly at a rate based on a slight increase in IL-6, CRP, and PI/I ratio[25]:

$$\frac{dIL1b(t)}{dt} = kplacebo \tag{13}$$

IL-1Ra. The local concentration of endogenous IL-1Ra was assumed constant in all simulations:

$$\frac{dIL1Ra(t)}{dt} = 0 \tag{14}$$

Non-diseased and diseased states

Non-diseased steady-state

Similar to the work of de Gaetano *et al.*[1], we considered a non-diseased steady-state corresponding to a glucose concentration of 5 mM and an insulin concentration of 50 pM. Furthermore, the steady-state proinsulin concentration was set to 6.5 pM, corresponding to a PI/I ratio of 0.13[27], and normal β-cell mass was set to 100 %. Finally, steady-state values for local IL-1β and IL-1Ra levels were determined according to the following reasoning: Average serum IL-1β and IL-1Ra concentrations in non-diabetic patients have been reported to be in the region of 0.5 pg/ml and 250 pg/ml respectively[28, 29]. Considering that both IL-1β and IL-1Ra are produced locally at the islets, however, the concentrations close to the β-cells might be much higher. For instance, Lachmann *et al*.[24] present a model of in-vivo regulation of IL-1β in patients with cryopyrin-associated periodic syndromes showing a 100-fold difference between IL-1β plasma concentrations and the concentration at the site of IL-1β production. By assuming the same difference in our case, and that the ratio of local IL-1β and IL-1Ra is similar to that in plasma – which is supported by the observed ratio of IL-1β and IL-1Ra secretion from human islets *in vitro*[30] – we arrived at local IL-1β and IL-1Ra concentrations of 50 pg/ml and 25 ng/ml respectively.

Diseased state

The diseased state was defined considering a reported average FPG concentration of 10.8 mM[25], an insulin concentration of 100 pM, and a 60 % decrease in β-cell mass[16]. This allowed us to calculate required changes in insulin sensitivity (to 22 % of normal), insulin secretion capacity (to 64 % of normal), and IL-1R modulation. Furthermore, by assuming a slightly increased level of local IL-1Ra (to 40 ng/ml) based on a reported 1.6 fold increase seen in serum at the time of T2DM diagnosis[28], we arrived at a required 100 fold increase in local IL-1β. Such a drastic increase is in line with an observed 2-200 fold increase in IL-1β mRNA expression seen in β-cells from patients with T2DM[31]. Both the non-diseased and diseased states are summarized in Supplementary Table 1.

References

1. De Gaetano A, Hardy T, Beck B, Abu-Raddad E, Palumbo P, Bue-Valleskey J, *et al.* Mathematical models of diabetes progression. *Am J Physiol Endocrinol Metab* 2008, **295**(6)**:** E1462-1479.

2. Topp B, Promislow K, deVries G, Miura RM, Finegood DT. A model of beta-cell mass, insulin, and glucose kinetics: pathways to diabetes. *J Theor Biol* 2000, **206**(4)**:** 605-619.

3. Podlecki DA, Frank BH, Olefsky JM. In vitro characterization of biosynthetic human proinsulin. *Diabetes* 1984, **33**(2)**:** 111-118.

4. Hamren B, Bjork E, Sunzel M, Karlsson M. Models for plasma glucose, HbA1c, and hemoglobin interrelationships in patients with type 2 diabetes following tesaglitazar treatment. *Clin Pharmacol Ther* 2008, **84**(2)**:** 228-235.

5. Lledo-Garcia R, Kalicki RM, Uehlinger DE, Karlsson MO. Modeling of red blood cell life-spans in hematologically normal populations. *Journal of pharmacokinetics and pharmacodynamics* 2012, **39**(5)**:** 453-462.

6. Lledo-Garcia R, Mazer NA, Karlsson MO. A semi-mechanistic model of the relationship between average glucose and HbA1c in healthy and diabetic subjects. *Journal of pharmacokinetics and pharmacodynamics* 2013, **40**(2)**:** 129-142.

7. Tura A, Pacini G, Kautzky-Willer A, Ludvik B, Prager R, Thomaseth K. Basal and dynamic proinsulin-insulin relationship to assess beta-cell function during OGTT in metabolic disorders. *Am J Physiol Endocrinol Metab* 2003, **285**(1)**:** E155-162.

8. Dinarello CA. Biologic basis for interleukin-1 in disease. *Blood* 1996, **87**(6)**:** 2095-2147.

9. Maedler K, Schumann DM, Sauter N, Ellingsgaard H, Bosco D, Baertschiger R, *et al.* Low concentration of interleukin-1beta induces FLICE-inhibitory protein-mediated beta-cell proliferation in human pancreatic islets. *Diabetes* 2006, **55**(10)**:** 2713-2722.

10. Maedler K, Sergeev P, Ris F, Oberholzer J, Joller-Jemelka HI, Spinas GA, *et al.* Glucose-induced beta cell production of IL-1beta contributes to glucotoxicity in human pancreatic islets. *J Clin Invest* 2002, **110**(6)**:** 851-860.

11. Spinas GA, Palmer JP, Mandrup-Poulsen T, Andersen H, Nielsen JH, Nerup J. The bimodal effect of interleukin 1 on rat pancreatic beta-cells--stimulation followed by inhibition--depends upon dose, duration of exposure, and ambient glucose concentration. *Acta Endocrinol (Copenh)* 1988, **119**(2)**:** 307-311.

12. Xiao X, Chen Z, Shiota C, Prasadan K, Guo P, El-Gohary Y, *et al.* No evidence for beta cell neogenesis in murine adult pancreas. *J Clin Invest* 2013, **123**(5)**:** 2207-2217.

13. Meier JJ, Butler AE, Saisho Y, Monchamp T, Galasso R, Bhushan A, *et al.* Beta-cell replication is the primary mechanism subserving the postnatal expansion of beta-cell mass in humans. *Diabetes* 2008, **57**(6)**:** 1584-1594.

14. Tyrberg B, Eizirik DL, Hellerstrom C, Pipeleers DG, Andersson A. Human pancreatic beta-cell deoxyribonucleic acid-synthesis in islet grafts decreases with increasing organ donor age but increases in response to glucose stimulation in vitro. *Endocrinology* 1996, **137**(12)**:** 5694-5699.

15. Perl S, Kushner JA, Buchholz BA, Meeker AK, Stein GM, Hsieh M, *et al.* Significant human beta-cell turnover is limited to the first three decades of life as determined by in vivo thymidine analog incorporation and radiocarbon dating. *J Clin Endocrinol Metab* 2010, **95**(10)**:** E234-239.

16. Cnop M, Hughes SJ, Igoillo-Esteve M, Hoppa MB, Sayyed F, van de Laar L, *et al.* The long lifespan and low turnover of human islet beta cells estimated by mathematical modelling of lipofuscin accumulation. *Diabetologia* 2010, **53**(2)**:** 321-330.

17. Butler AE, Janson J, Bonner-Weir S, Ritzel R, Rizza RA, Butler PC. Beta-cell deficit and increased beta-cell apoptosis in humans with type 2 diabetes. *Diabetes* 2003, **52**(1)**:** 102-110.

18. Marchetti P, Bugliani M, Lupi R, Marselli L, Masini M, Boggi U, *et al.* The endoplasmic reticulum in pancreatic beta cells of type 2 diabetes patients. *Diabetologia* 2007, **50**(12)**:** 2486-2494.

19. Borjesson A, Carlsson C. Altered proinsulin conversion in rat pancreatic islets exposed long-term to various glucose concentrations or interleukin-1beta. *J Endocrinol* 2007, **192**(2)**:** 381-387.

20. Hostens K, Pavlovic D, Zambre Y, Ling Z, Van Schravendijk C, Eizirik DL, *et al.* Exposure of human islets to cytokines can result in disproportionately elevated proinsulin release. *J Clin Invest* 1999, **104**(1)**:** 67-72.

21. Alarcon C, Leahy JL, Schuppin GT, Rhodes CJ. Increased secretory demand rather than a defect in the proinsulin conversion mechanism causes hyperproinsulinemia in a glucose-infusion rat model of non-insulin-dependent diabetes mellitus. *J Clin Invest* 1995, **95**(3)**:** 1032-1039.

22. Yang BB, Baughman S, Sullivan JT. Pharmacokinetics of anakinra in subjects with different levels of renal function. *Clin Pharmacol Ther* 2003, **74**(1)**:** 85-94.

23. Chang DM, Chang SY, Yeh MK, Lai JH. The pharmacokinetics of interleukin-1 receptor antagonist in Chinese subjects with rheumatoid arthritis. *Pharmacol Res* 2004, **50**(3)**:** 371-376.

24. Lachmann HJ, Lowe P, Felix SD, Rordorf C, Leslie K, Madhoo S, *et al.* In vivo regulation of interleukin 1beta in patients with cryopyrin-associated periodic syndromes. *J Exp Med* 2009, **206**(5)**:** 1029-1036.

25. Larsen CM, Faulenbach M, Vaag A, Volund A, Ehses JA, Seifert B, *et al.* Interleukin-1-receptor antagonist in type 2 diabetes mellitus. *N Engl J Med* 2007, **356**(15)**:** 1517-1526.

26. Larsen CM, Faulenbach M, Vaag A, Ehses JA, Donath MY, Mandrup-Poulsen T. Sustained effects of interleukin-1 receptor antagonist treatment in type 2 diabetes. *Diabetes Care* 2009, **32**(9)**:** 1663-1668.

27. Pradhan AD, Manson JE, Meigs JB, Rifai N, Buring JE, Liu S, *et al.* Insulin, proinsulin, proinsulin:insulin ratio, and the risk of developing type 2 diabetes mellitus in women. *The American journal of medicine* 2003, **114**(6)**:** 438-444.

28. Carstensen M, Herder C, Kivimaki M, Jokela M, Roden M, Shipley MJ, *et al.* Accelerated increase in serum interleukin-1 receptor antagonist starts 6 years before diagnosis of type 2 diabetes: Whitehall II prospective cohort study. *Diabetes* 2010, **59**(5)**:** 1222-1227.

29. Spranger J, Kroke A, Mohlig M, Hoffmann K, Bergmann MM, Ristow M, *et al.* Inflammatory cytokines and the risk to develop type 2 diabetes: results of the prospective population-based European Prospective Investigation into Cancer and Nutrition (EPIC)-Potsdam Study. *Diabetes* 2003, **52**(3)**:** 812-817.

30. Maedler K, Sergeev P, Ehses JA, Mathe Z, Bosco D, Berney T, *et al.* Leptin modulates beta cell expression of IL-1 receptor antagonist and release of IL-1beta in human islets. *Proc Natl Acad Sci U S A* 2004, **101**(21)**:** 8138-8143.

31. Boni-Schnetzler M, Thorne J, Parnaud G, Marselli L, Ehses JA, Kerr-Conte J, *et al.* Increased interleukin (IL)-1beta messenger ribonucleic acid expression in beta -cells of individuals with type 2 diabetes and regulation of IL-1beta in human islets by glucose and autostimulation. *J Clin Endocrinol Metab* 2008, **93**(10)**:** 4065-4074.
