# Supplementary material for: Effects of IL-1β–Blocking Therapies in Type 2 Diabetes Mellitus: A Quantitative Systems Pharmacology Modeling Approach to Explore Underlying Mechanisms
Source: CPT Pharmacometrics Syst Pharmacol. 2014 Jun 11;3(6):e118–. doi: 10.1038/psp.2014.16 (PMC4076803; doi:10.1038/psp.2014.16)
Supplement: Supplementary Notepad [file psp201416x5.doc]

(* Content-type: application/vnd.wolfram.mathematica *)

(*** Wolfram Notebook File ***)

(* http://www.wolfram.com/nb *)

(* CreatedBy='Mathematica 10.0' *)

(*CacheID: 234*)

(* Internal cache information:

NotebookFileLineBreakTest

NotebookFileLineBreakTest

NotebookDataPosition[ 158, 7]

NotebookDataLength[ 109949, 2915]

NotebookOptionsPosition[ 106677, 2805]

NotebookOutlinePosition[ 107024, 2820]

CellTagsIndexPosition[ 106981, 2817]

WindowFrame->Normal*)

(* Beginning of Notebook Content *)

Notebook[{

Cell[CellGroupData[{

Cell["\<\

Effects of IL - 1 \[Beta] Blocking Therapies in Type 2 Diabetes Mellitus\

\>", "Title",

CellChangeTimes->{{3.602937750328635*^9, 3.6029377662935867`*^9}}],

Cell["\<\

A Quantitative Systems Pharmacology Modeling Approach to Explore Underlying \

Mechanisms\

\>", "Subtitle",

CellChangeTimes->{{3.602937750328635*^9, 3.6029377682620645`*^9}}],

Cell["\<\

Robert Palm\[EAcute]r, Elin Nyman, Mark Penney, Anna Marley, Gunnar \

Cedersund, and Balaji Agoram\

\>", "Subsubtitle",

CellChangeTimes->{{3.602937783797902*^9, 3.6029377950616837`*^9}, {

3.602937840997031*^9, 3.602937864084545*^9}}],

Cell[CellGroupData[{

Cell["Model equations", "Section",

CellChangeTimes->{{3.6023851502450314`*^9, 3.602385152709641*^9}, {

3.6024232977013702`*^9, 3.602423301781378*^9}}],

Cell[CellGroupData[{

Cell["Glucose-insulin system", "Subsection",

CellChangeTimes->{{3.5788027200433903`*^9, 3.5788027246666546`*^9}}],

Cell[BoxData[

RowBox[{

RowBox[{"eqsGlucoseInsulin", "=",

RowBox[{"{", "\[IndentingNewLine]",

RowBox[{

RowBox[{

RowBox[{"glucose", "[", "t", "]"}], "\[Equal]",

FractionBox["Tgl",

RowBox[{"Kxg", "+",

RowBox[{"Kxgi", "*",

RowBox[{"insulin", "[", "t", "]"}]}], "+",

RowBox[{"0.1", "*", "Kxgi", "*",

RowBox[{"proinsulin", "[", "t", "]"}]}]}]]}], ",",

"\[IndentingNewLine]",

RowBox[{

RowBox[{"insulin", "[", "t", "]"}], "\[Equal]",

RowBox[{

FractionBox[

RowBox[{

RowBox[{"b", "[", "t", "]"}], "*",

RowBox[{"isc", "[", "t", "]"}]}], "Kxi"], "*",

FractionBox[

SuperscriptBox[

RowBox[{"(",

FractionBox[

RowBox[{"glucose", "[", "t", "]"}], "Gh"], ")"}], "vh"],

RowBox[{"1", "+",

SuperscriptBox[

RowBox[{"(",

FractionBox[

RowBox[{"glucose", "[", "t", "]"}], "Gh"], ")"}], "vh"]}]]}]}],

",", "\[IndentingNewLine]",

RowBox[{

RowBox[{"proinsulin", "[", "t", "]"}], "\[Equal]",

RowBox[{

RowBox[{"f", "[", "t", "]"}], "*",

FractionBox[

RowBox[{

RowBox[{"b", "[", "t", "]"}], "*",

RowBox[{"isc", "[", "t", "]"}]}],

RowBox[{"0.1", "*", "Kxi"}]], "*",

FractionBox[

SuperscriptBox[

RowBox[{"(",

FractionBox[

RowBox[{"glucose", "[", "t", "]"}], "Gh"], ")"}], "vh"],

RowBox[{"1", "+",

SuperscriptBox[

RowBox[{"(",

FractionBox[

RowBox[{"glucose", "[", "t", "]"}], "Gh"], ")"}], "vh"]}]]}]}]}],

"\[IndentingNewLine]", "}"}]}], ";"}]], "Input",

CellChangeTimes->{

3.5788028638346148`*^9, {3.578803419367389*^9, 3.5788034395945463`*^9}, {

3.5788041502141914`*^9, 3.578804162917918*^9}, {3.5794140773727293`*^9,

3.579414077772752*^9}, {3.5794143818021417`*^9, 3.5794143876334753`*^9},

3.5828787703236184`*^9, {3.5828791681303716`*^9, 3.5828791695974555`*^9},

3.5830306674738426`*^9, 3.5830309190392313`*^9}],

Cell[BoxData[

RowBox[{

RowBox[{"paramsGlucoseInsulin", "=",

RowBox[{"{", "\[IndentingNewLine]",

RowBox[{

RowBox[{"Kxg", "\[Rule]", "0.000016"}], ",", "\[IndentingNewLine]",

RowBox[{"Kxgi", "\[Rule]", "0.0001"}], ",", "\[IndentingNewLine]",

RowBox[{"Kxi", "\[Rule]", " ", "0.05"}], ",", "\[IndentingNewLine]",

RowBox[{"Gh", "\[Rule]", "9"}], ",", "\[IndentingNewLine]",

RowBox[{"vh", "\[Rule]", "4"}]}], " ", "\[IndentingNewLine]", "}"}]}],

";"}]], "Input",

CellChangeTimes->{{3.578803117680134*^9, 3.578803127384689*^9}, {

3.578803159064501*^9, 3.5788031859470387`*^9}, {3.578803229705541*^9,

3.578803230712599*^9}, {3.578803454534401*^9, 3.5788035189030824`*^9}, {

3.582902562377446*^9, 3.582902568312785*^9}, {3.5829026769039965`*^9,

3.5829026791601257`*^9}, {3.589607524348584*^9, 3.5896075398323793`*^9}, {

3.5896083899063435`*^9, 3.5896083918268127`*^9}, 3.5925540741114063`*^9, {

3.6023854884044237`*^9, 3.602385499412143*^9}}]

}, Closed]],

Cell[CellGroupData[{

Cell["HbA1c", "Subsection",

CellChangeTimes->{{3.5788027200433903`*^9, 3.5788027246666546`*^9}, {

3.5788036758600597`*^9, 3.5788036781481905`*^9}, {3.6023852440842094`*^9,

3.602385244772379*^9}}],

Cell[BoxData[

RowBox[{

RowBox[{"eqsHbA1C", "=",

RowBox[{"{", "\[IndentingNewLine]",

RowBox[{

RowBox[{

RowBox[{"hba1c", "[", "t", "]"}], "\[Equal]",

FractionBox[

RowBox[{"100", "*",

RowBox[{"(",

RowBox[{

RowBox[{"a1c1", "[", "t", "]"}], "+",

RowBox[{"a1c2", "[", "t", "]"}], "+",

RowBox[{"a1c3", "[", "t", "]"}], "+",

RowBox[{"a1c4", "[", "t", "]"}], "+",

RowBox[{"a1c5", "[", "t", "]"}], "+",

RowBox[{"a1c6", "[", "t", "]"}], "+",

RowBox[{"a1c7", "[", "t", "]"}], "+",

RowBox[{"a1c8", "[", "t", "]"}], "+",

RowBox[{"a1c9", "[", "t", "]"}], "+",

RowBox[{"a1c10", "[", "t", "]"}], "+",

RowBox[{"a1c11", "[", "t", "]"}], "+",

RowBox[{"a1c12", "[", "t", "]"}]}], ")"}]}],

RowBox[{"(",

RowBox[{

RowBox[{"a1c1", "[", "t", "]"}], "+",

RowBox[{"a1c2", "[", "t", "]"}], "+",

RowBox[{"a1c3", "[", "t", "]"}], "+",

RowBox[{"a1c4", "[", "t", "]"}], "+",

RowBox[{"a1c5", "[", "t", "]"}], "+",

RowBox[{"a1c6", "[", "t", "]"}], "+",

RowBox[{"a1c7", "[", "t", "]"}], "+",

RowBox[{"a1c8", "[", "t", "]"}], "+",

RowBox[{"a1c9", "[", "t", "]"}], "+",

RowBox[{"a1c10", "[", "t", "]"}], "+",

RowBox[{"a1c11", "[", "t", "]"}], "+",

RowBox[{"a1c12", "[", "t", "]"}], "+",

RowBox[{"rbc1", "[", "t", "]"}], "+",

RowBox[{"rbc2", "[", "t", "]"}], "+",

RowBox[{"rbc3", "[", "t", "]"}], "+",

RowBox[{"rbc4", "[", "t", "]"}], "+",

RowBox[{"rbc5", "[", "t", "]"}], "+",

RowBox[{"rbc6", "[", "t", "]"}], "+",

RowBox[{"rbc7", "[", "t", "]"}], "+",

RowBox[{"rbc8", "[", "t", "]"}], "+",

RowBox[{"rbc9", "[", "t", "]"}], "+",

RowBox[{"rbc10", "[", "t", "]"}], "+",

RowBox[{"rbc11", "[", "t", "]"}], "+",

RowBox[{"rbc12", "[", "t", "]"}]}], ")"}]]}], ",",

"\[IndentingNewLine]",

RowBox[{

RowBox[{

RowBox[{"rbc1", "'"}], "[", "t", "]"}], "\[Equal]",

RowBox[{"Kin", "-",

RowBox[{"Ktr", "*",

RowBox[{"rbc1", "[", "t", "]"}]}], "-",

RowBox[{"Kglucose", "*",

SuperscriptBox[

RowBox[{"glucose", "[", "t", "]"}], "lambda"], "*",

RowBox[{"rbc1", "[", "t", "]"}]}]}]}], ",", "\[IndentingNewLine]",

RowBox[{

RowBox[{

RowBox[{"rbc2", "'"}], "[", "t", "]"}], "\[Equal]",

RowBox[{

RowBox[{"Ktr", "*",

RowBox[{"rbc1", "[", "t", "]"}]}], "-",

RowBox[{"Ktr", "*",

RowBox[{"rbc2", "[", "t", "]"}]}], "-",

RowBox[{"Kglucose", "*",

SuperscriptBox[

RowBox[{"glucose", "[", "t", "]"}], "lambda"], "*",

RowBox[{"rbc2", "[", "t", "]"}]}]}]}], ",", "\[IndentingNewLine]",

RowBox[{

RowBox[{

RowBox[{"rbc3", "'"}], "[", "t", "]"}], "\[Equal]",

RowBox[{

RowBox[{"Ktr", "*",

RowBox[{"rbc2", "[", "t", "]"}]}], "-",

RowBox[{"Ktr", "*",

RowBox[{"rbc3", "[", "t", "]"}]}], "-",

RowBox[{"Kglucose", "*",

SuperscriptBox[

RowBox[{"glucose", "[", "t", "]"}], "lambda"], "*",

RowBox[{"rbc3", "[", "t", "]"}]}]}]}], ",", "\[IndentingNewLine]",

RowBox[{

RowBox[{

RowBox[{"rbc4", "'"}], "[", "t", "]"}], "\[Equal]",

RowBox[{

RowBox[{"Ktr", "*",

RowBox[{"rbc3", "[", "t", "]"}]}], "-",

RowBox[{"Ktr", "*",

RowBox[{"rbc4", "[", "t", "]"}]}], "-",

RowBox[{"Kglucose", "*",

SuperscriptBox[

RowBox[{"glucose", "[", "t", "]"}], "lambda"], "*",

RowBox[{"rbc4", "[", "t", "]"}]}]}]}], ",", "\[IndentingNewLine]",

RowBox[{

RowBox[{

RowBox[{"rbc5", "'"}], "[", "t", "]"}], "\[Equal]",

RowBox[{

RowBox[{"Ktr", "*",

RowBox[{"rbc4", "[", "t", "]"}]}], "-",

RowBox[{"Ktr", "*",

RowBox[{"rbc5", "[", "t", "]"}]}], "-",

RowBox[{"Kglucose", "*",

SuperscriptBox[

RowBox[{"glucose", "[", "t", "]"}], "lambda"], "*",

RowBox[{"rbc5", "[", "t", "]"}]}]}]}], ",", "\[IndentingNewLine]",

RowBox[{

RowBox[{

RowBox[{"rbc6", "'"}], "[", "t", "]"}], "\[Equal]",

RowBox[{

RowBox[{"Ktr", "*",

RowBox[{"rbc5", "[", "t", "]"}]}], "-",

RowBox[{"Ktr", "*",

RowBox[{"rbc6", "[", "t", "]"}]}], "-",

RowBox[{"Kglucose", "*",

SuperscriptBox[

RowBox[{"glucose", "[", "t", "]"}], "lambda"], "*",

RowBox[{"rbc6", "[", "t", "]"}]}]}]}], ",", "\[IndentingNewLine]",

RowBox[{

RowBox[{

RowBox[{"rbc7", "'"}], "[", "t", "]"}], "\[Equal]",

RowBox[{

RowBox[{"Ktr", "*",

RowBox[{"rbc6", "[", "t", "]"}]}], "-",

RowBox[{"Ktr", "*",

RowBox[{"rbc7", "[", "t", "]"}]}], "-",

RowBox[{"Kglucose", "*",

SuperscriptBox[

RowBox[{"glucose", "[", "t", "]"}], "lambda"], "*",

RowBox[{"rbc7", "[", "t", "]"}]}]}]}], ",", "\[IndentingNewLine]",

RowBox[{

RowBox[{

RowBox[{"rbc8", "'"}], "[", "t", "]"}], "\[Equal]",

RowBox[{

RowBox[{"Ktr", "*",

RowBox[{"rbc7", "[", "t", "]"}]}], "-",

RowBox[{"Ktr", "*",

RowBox[{"rbc8", "[", "t", "]"}]}], "-",

RowBox[{"Kglucose", "*",

SuperscriptBox[

RowBox[{"glucose", "[", "t", "]"}], "lambda"], "*",

RowBox[{"rbc8", "[", "t", "]"}]}]}]}], ",", "\[IndentingNewLine]",

RowBox[{

RowBox[{

RowBox[{"rbc9", "'"}], "[", "t", "]"}], "\[Equal]",

RowBox[{

RowBox[{"Ktr", "*",

RowBox[{"rbc8", "[", "t", "]"}]}], "-",

RowBox[{"Ktr", "*",

RowBox[{"rbc9", "[", "t", "]"}]}], "-",

RowBox[{"Kglucose", "*",

SuperscriptBox[

RowBox[{"glucose", "[", "t", "]"}], "lambda"], "*",

RowBox[{"rbc9", "[", "t", "]"}]}]}]}], ",", "\[IndentingNewLine]",

RowBox[{

RowBox[{

RowBox[{"rbc10", "'"}], "[", "t", "]"}], "\[Equal]",

RowBox[{

RowBox[{"Ktr", "*",

RowBox[{"rbc9", "[", "t", "]"}]}], "-",

RowBox[{"Ktr", "*",

RowBox[{"rbc10", "[", "t", "]"}]}], "-",

RowBox[{"Kglucose", "*",

SuperscriptBox[

RowBox[{"glucose", "[", "t", "]"}], "lambda"], "*",

RowBox[{"rbc10", "[", "t", "]"}]}]}]}], ",", "\[IndentingNewLine]",

RowBox[{

RowBox[{

RowBox[{"rbc11", "'"}], "[", "t", "]"}], "\[Equal]",

RowBox[{

RowBox[{"Ktr", "*",

RowBox[{"rbc10", "[", "t", "]"}]}], "-",

RowBox[{"Ktr", "*",

RowBox[{"rbc11", "[", "t", "]"}]}], "-",

RowBox[{"Kglucose", "*",

SuperscriptBox[

RowBox[{"glucose", "[", "t", "]"}], "lambda"], "*",

RowBox[{"rbc11", "[", "t", "]"}]}]}]}], ",", "\[IndentingNewLine]",

RowBox[{

RowBox[{

RowBox[{"rbc12", "'"}], "[", "t", "]"}], "\[Equal]",

RowBox[{

RowBox[{"Ktr", "*",

RowBox[{"rbc11", "[", "t", "]"}]}], "-",

RowBox[{"Ktr", "*",

RowBox[{"rbc12", "[", "t", "]"}]}], "-",

RowBox[{"Kglucose", "*",

SuperscriptBox[

RowBox[{"glucose", "[", "t", "]"}], "lambda"], "*",

RowBox[{"rbc12", "[", "t", "]"}]}]}]}], ",", "\[IndentingNewLine]",

RowBox[{

RowBox[{

RowBox[{"a1c1", "'"}], "[", "t", "]"}], "\[Equal]",

RowBox[{

RowBox[{"Kglucose", "*",

SuperscriptBox[

RowBox[{"glucose", "[", "t", "]"}], "lambda"], "*",

RowBox[{"rbc1", "[", "t", "]"}]}], "-",

RowBox[{"Ktr", "*",

RowBox[{"a1c1", "[", "t", "]"}]}]}]}], ",", "\[IndentingNewLine]",

RowBox[{

RowBox[{

RowBox[{"a1c2", "'"}], "[", "t", "]"}], "\[Equal]",

RowBox[{

RowBox[{"Kglucose", "*",

SuperscriptBox[

RowBox[{"glucose", "[", "t", "]"}], "lambda"], "*",

RowBox[{"rbc2", "[", "t", "]"}]}], "+",

RowBox[{"Ktr", "*",

RowBox[{"a1c1", "[", "t", "]"}]}], "-",

RowBox[{"Ktr", "*",

RowBox[{"a1c2", "[", "t", "]"}]}]}]}], ",", "\[IndentingNewLine]",

RowBox[{

RowBox[{

RowBox[{"a1c3", "'"}], "[", "t", "]"}], "\[Equal]",

RowBox[{

RowBox[{"Kglucose", "*",

SuperscriptBox[

RowBox[{"glucose", "[", "t", "]"}], "lambda"], "*",

RowBox[{"rbc3", "[", "t", "]"}]}], "+",

RowBox[{"Ktr", "*",

RowBox[{"a1c2", "[", "t", "]"}]}], "-",

RowBox[{"Ktr", "*",

RowBox[{"a1c3", "[", "t", "]"}]}]}]}], ",", "\[IndentingNewLine]",

RowBox[{

RowBox[{

RowBox[{"a1c4", "'"}], "[", "t", "]"}], "\[Equal]",

RowBox[{

RowBox[{"Kglucose", "*",

SuperscriptBox[

RowBox[{"glucose", "[", "t", "]"}], "lambda"], "*",

RowBox[{"rbc4", "[", "t", "]"}]}], "+",

RowBox[{"Ktr", "*",

RowBox[{"a1c3", "[", "t", "]"}]}], "-",

RowBox[{"Ktr", "*",

RowBox[{"a1c4", "[", "t", "]"}]}]}]}], ",", "\[IndentingNewLine]",

RowBox[{

RowBox[{

RowBox[{"a1c5", "'"}], "[", "t", "]"}], "\[Equal]",

RowBox[{

RowBox[{"Kglucose", "*",

SuperscriptBox[

RowBox[{"glucose", "[", "t", "]"}], "lambda"], "*",

RowBox[{"rbc5", "[", "t", "]"}]}], "+",

RowBox[{"Ktr", "*",

RowBox[{"a1c4", "[", "t", "]"}]}], "-",

RowBox[{"Ktr", "*",

RowBox[{"a1c5", "[", "t", "]"}]}]}]}], ",", "\[IndentingNewLine]",

RowBox[{

RowBox[{

RowBox[{"a1c6", "'"}], "[", "t", "]"}], "\[Equal]",

RowBox[{

RowBox[{"Kglucose", "*",

SuperscriptBox[

RowBox[{"glucose", "[", "t", "]"}], "lambda"], "*",

RowBox[{"rbc6", "[", "t", "]"}]}], "+",

RowBox[{"Ktr", "*",

RowBox[{"a1c5", "[", "t", "]"}]}], "-",

RowBox[{"Ktr", "*",

RowBox[{"a1c6", "[", "t", "]"}]}]}]}], ",", "\[IndentingNewLine]",

RowBox[{

RowBox[{

RowBox[{"a1c7", "'"}], "[", "t", "]"}], "\[Equal]",

RowBox[{

RowBox[{"Kglucose", "*",

SuperscriptBox[

RowBox[{"glucose", "[", "t", "]"}], "lambda"], "*",

RowBox[{"rbc7", "[", "t", "]"}]}], "+",

RowBox[{"Ktr", "*",

RowBox[{"a1c6", "[", "t", "]"}]}], "-",

RowBox[{"Ktr", "*",

RowBox[{"a1c7", "[", "t", "]"}]}]}]}], ",", "\[IndentingNewLine]",

RowBox[{

RowBox[{

RowBox[{"a1c8", "'"}], "[", "t", "]"}], "\[Equal]",

RowBox[{

RowBox[{"Kglucose", "*",

SuperscriptBox[

RowBox[{"glucose", "[", "t", "]"}], "lambda"], "*",

RowBox[{"rbc8", "[", "t", "]"}]}], "+",

RowBox[{"Ktr", "*",

RowBox[{"a1c7", "[", "t", "]"}]}], "-",

RowBox[{"Ktr", "*",

RowBox[{"a1c8", "[", "t", "]"}]}]}]}], ",", "\[IndentingNewLine]",

RowBox[{

RowBox[{

RowBox[{"a1c9", "'"}], "[", "t", "]"}], "\[Equal]",

RowBox[{

RowBox[{"Kglucose", "*",

SuperscriptBox[

RowBox[{"glucose", "[", "t", "]"}], "lambda"], "*",

RowBox[{"rbc9", "[", "t", "]"}]}], "+",

RowBox[{"Ktr", "*",

RowBox[{"a1c8", "[", "t", "]"}]}], "-",

RowBox[{"Ktr", "*",

RowBox[{"a1c9", "[", "t", "]"}]}]}]}], ",", "\[IndentingNewLine]",

RowBox[{

RowBox[{

RowBox[{"a1c10", "'"}], "[", "t", "]"}], "\[Equal]",

RowBox[{

RowBox[{"Kglucose", "*",

SuperscriptBox[

RowBox[{"glucose", "[", "t", "]"}], "lambda"], "*",

RowBox[{"rbc10", "[", "t", "]"}]}], "+",

RowBox[{"Ktr", "*",

RowBox[{"a1c9", "[", "t", "]"}]}], "-",

RowBox[{"Ktr", "*",

RowBox[{"a1c10", "[", "t", "]"}]}]}]}], ",", "\[IndentingNewLine]",

RowBox[{

RowBox[{

RowBox[{"a1c11", "'"}], "[", "t", "]"}], "\[Equal]",

RowBox[{

RowBox[{"Kglucose", "*",

SuperscriptBox[

RowBox[{"glucose", "[", "t", "]"}], "lambda"], "*",

RowBox[{"rbc11", "[", "t", "]"}]}], "+",

RowBox[{"Ktr", "*",

RowBox[{"a1c10", "[", "t", "]"}]}], "-",

RowBox[{"Ktr", "*",

RowBox[{"a1c11", "[", "t", "]"}]}]}]}], ",", "\[IndentingNewLine]",

RowBox[{

RowBox[{

RowBox[{"a1c12", "'"}], "[", "t", "]"}], "\[Equal]",

RowBox[{

RowBox[{"Kglucose", "*",

SuperscriptBox[

RowBox[{"glucose", "[", "t", "]"}], "lambda"], "*",

RowBox[{"rbc12", "[", "t", "]"}]}], "+",

RowBox[{"Ktr", "*",

RowBox[{"a1c11", "[", "t", "]"}]}], "-",

RowBox[{"Ktr", "*",

RowBox[{"a1c12", "[", "t", "]"}]}]}]}]}], "\[IndentingNewLine]",

"}"}]}], ";"}]], "Input",

CellChangeTimes->{

3.5788028638346148`*^9, 3.5788037031456203`*^9, {3.578804241676423*^9,

3.5788042819107237`*^9}, {3.578902475750436*^9, 3.578902495888436*^9}, {

3.591684531672365*^9, 3.5916845822778635`*^9}, {3.5916858328630657`*^9,

3.591685856477877*^9}, {3.5916905997888966`*^9, 3.591690820395253*^9}, {

3.591691105809519*^9, 3.5916911294312367`*^9}, {3.591693177640375*^9,

3.59169324238931*^9}, {3.5916941118189692`*^9, 3.591694125001214*^9}, {

3.591694235834489*^9, 3.5916943030650344`*^9}, {3.5916955060180817`*^9,

3.5916955383360343`*^9}, {3.591697452627227*^9, 3.5916974823705907`*^9}, {

3.5916983670849857`*^9, 3.5916984436241045`*^9}, 3.59169876711779*^9,

3.5917014358984013`*^9, 3.591701604232871*^9, {3.6029379567374296`*^9,

3.6029379855895557`*^9}}],

Cell[BoxData[

RowBox[{

RowBox[{"paramsHbA1C", "=",

RowBox[{"{", "\[IndentingNewLine]",

RowBox[{

RowBox[{"Ktr", "\[Rule]",

RowBox[{"12", "/", "100"}]}], ",", "\[IndentingNewLine]",

RowBox[{"Kin", "\[Rule]", "1.05"}], ",", "\[IndentingNewLine]",

RowBox[{"lambda", "\[Rule]", "0.743"}], ",", "\[IndentingNewLine]",

RowBox[{"Kglucose", "\[Rule]", "0.000292"}]}], "\[IndentingNewLine]",

"}"}]}], ";"}]], "Input",

CellChangeTimes->{{3.578803117680134*^9, 3.578803127384689*^9}, {

3.578803159064501*^9, 3.5788031859470387`*^9}, {3.578803229705541*^9,

3.578803230712599*^9}, {3.578803786500388*^9, 3.578803811813836*^9},

3.578901916814059*^9, {3.586223089102456*^9, 3.5862230891805887`*^9}, {

3.586223156467456*^9, 3.586223156936247*^9}, 3.5862242549240723`*^9,

3.586224449610101*^9, {3.591684587025032*^9, 3.5916845933445864`*^9},

3.5916858205380325`*^9, {3.591691141234141*^9, 3.5916911462743816`*^9}, {

3.591693323554284*^9, 3.5916933305940256`*^9}, {3.591694034524947*^9,

3.591694035933294*^9}, {3.591694307675169*^9, 3.591694312746417*^9}, {

3.5916954705783587`*^9, 3.591695488482765*^9}, 3.5916955235704007`*^9, {

3.5916975513156013`*^9, 3.591697551603672*^9}, {3.591698160223034*^9,

3.591698166207506*^9}, {3.5916988400267324`*^9, 3.591698840154763*^9},

3.591699288055398*^9, 3.591699536709592*^9, {3.5916995827249165`*^9,

3.591699583253047*^9}, {3.59169976194005*^9, 3.5916997641956053`*^9},

3.5917003499849*^9, {3.5917013723766985`*^9, 3.591701392552664*^9},

3.591701685014524*^9, {3.5917018576847744`*^9, 3.591701869560479*^9}, {

3.592214477108962*^9, 3.592214478453293*^9}, 3.592285247334389*^9, {

3.602385478564993*^9, 3.602385485139617*^9}, {3.6024230078967876`*^9,

3.602423036062745*^9}}]

}, Closed]],

Cell[CellGroupData[{

Cell["IL-1\[Beta] subsystem and effects on \[Beta]-cell turnover and \

function", "Subsection",

CellChangeTimes->{{3.578804974171319*^9, 3.5788049976906643`*^9}}],

Cell[CellGroupData[{

Cell["IL-1\[Beta] receptor modulation", "Subsubsection",

CellChangeTimes->{{3.578805007499225*^9, 3.578805019402906*^9}, {

3.602385461330738*^9, 3.602385462322981*^9}}],

Cell[BoxData[

RowBox[{

RowBox[{"eqModulation", "=",

RowBox[{"{", "\[IndentingNewLine]",

RowBox[{

RowBox[{"il1r", "[", "t", "]"}], "\[Equal]",

FractionBox[

RowBox[{"il1b", "[", "t", "]"}],

RowBox[{

RowBox[{"km", " ",

RowBox[{"(",

RowBox[{"1", "+",

FractionBox[

RowBox[{"il1ra", "[", "t", "]"}], "ki"]}], ")"}]}], "+",

RowBox[{"il1b", "[", "t", "]"}]}]]}], "\[IndentingNewLine]", "}"}]}],

";"}]], "Input",

CellChangeTimes->{{3.578805035963853*^9, 3.57880507646017*^9},

3.578825897289053*^9, {3.592284583595044*^9, 3.5922845847783394`*^9}, {

3.5922849652559705`*^9, 3.5922849656090574`*^9}, {3.602385458052927*^9,

3.6023854593162394`*^9}}],

Cell[BoxData[

RowBox[{

RowBox[{"paramsModulation", " ", "=",

RowBox[{"{", "\[IndentingNewLine]",

RowBox[{

RowBox[{"km", "\[Rule]", "8.5"}], ",", "\[IndentingNewLine]",

RowBox[{"ki", "\[Rule]", "1.7"}]}], "\[IndentingNewLine]", "}"}]}],

";"}]], "Input",

CellChangeTimes->{{3.5788050950962353`*^9, 3.5788051056908417`*^9}, {

3.57880803747853*^9, 3.5788080789499025`*^9}, {3.5788081689670506`*^9,

3.5788083019566574`*^9}, {3.5921226786780066`*^9, 3.592122698053775*^9}, {

3.5921954987807093`*^9, 3.592195499020767*^9}, {3.5921959903137136`*^9,

3.592195998729785*^9}, {3.592196166921177*^9, 3.592196167608345*^9}, {

3.592198181355363*^9, 3.5921981906976614`*^9}, {3.5922004072108145`*^9,

3.5922004078019605`*^9}, {3.592201979342854*^9, 3.5922019799660063`*^9}, {

3.592205044344479*^9, 3.5922050449366236`*^9}, {3.5922067231640453`*^9,

3.592206724284322*^9}, {3.5922834736995935`*^9, 3.5922834760511723`*^9}, {

3.6023855040993004`*^9, 3.602385506787964*^9}, {3.6023859819683332`*^9,

3.6023859829925866`*^9}}]

}, Closed]],

Cell[CellGroupData[{

Cell["\[Beta]-cell turnover", "Subsubsection",

CellChangeTimes->{{3.5788038748664427`*^9, 3.578803884706005*^9}, {

3.578808873151328*^9, 3.5788088902083035`*^9}, {3.602385379683571*^9,

3.6023853803237276`*^9}, {3.6023854190292892`*^9, 3.6023854212398343`*^9}}],

Cell[BoxData[

RowBox[{

RowBox[{"eqsTurnover", "=",

RowBox[{"{",

RowBox[{

RowBox[{

RowBox[{

SuperscriptBox["b", "\[Prime]",

MultilineFunction->None], "[", "t", "]"}], "\[Equal]",

RowBox[{

RowBox[{"b", "[", "t", "]"}], "*",

RowBox[{"(",

RowBox[{

RowBox[{"-",

RowBox[{"apoptosis", "[", "t", "]"}]}], "+",

RowBox[{"replication", "[", "t", "]"}]}], ")"}]}]}], ",",

"\[IndentingNewLine]",

RowBox[{

RowBox[{"apoptosis", "[", "t", "]"}], "\[Equal]",

RowBox[{"ka", " ",

RowBox[{"(",

RowBox[{"1", "-",

FractionBox[

RowBox[{"vla", " ",

SuperscriptBox[

RowBox[{"il1r", "[", "t", "]"}], "xla"]}],

RowBox[{

SuperscriptBox["kmla", "xla"], "+",

SuperscriptBox[

RowBox[{"il1r", "[", "t", "]"}], "xla"]}]], "+",

FractionBox[

RowBox[{"vha", " ",

SuperscriptBox[

RowBox[{"il1r", "[", "t", "]"}], "xha"]}],

RowBox[{

SuperscriptBox["kmha", "xha"], "+",

SuperscriptBox[

RowBox[{"il1r", "[", "t", "]"}], "xha"]}]]}], ")"}]}]}], ",",

"\[IndentingNewLine]",

RowBox[{

RowBox[{"replication", "[", "t", "]"}], "\[Equal]",

RowBox[{"kr", " ",

RowBox[{"(",

RowBox[{"1", "+",

FractionBox[

RowBox[{"vlr", " ",

SuperscriptBox[

RowBox[{"il1r", "[", "t", "]"}], "xlr"]}],

RowBox[{

SuperscriptBox["kmlr", "xlr"], "+",

SuperscriptBox[

RowBox[{"il1r", "[", "t", "]"}], "xlr"]}]], "-",

FractionBox[

RowBox[{"vhr", " ",

SuperscriptBox[

RowBox[{"il1r", "[", "t", "]"}], "xhr"]}],

RowBox[{

SuperscriptBox["kmhr", "xhr"], "+",

SuperscriptBox[

RowBox[{"il1r", "[", "t", "]"}], "xhr"]}]]}], ")"}]}]}]}],

"}"}]}], ";"}]], "Input",

CellChangeTimes->{{3.5781995506785307`*^9, 3.5781995709916925`*^9}, {

3.5781996236147027`*^9, 3.5781997381602545`*^9}, 3.57820321607218*^9, {

3.5782146356393414`*^9, 3.5782146552244616`*^9}, {3.5788040085800905`*^9,

3.578804017556604*^9}, {3.5788040667314167`*^9, 3.5788040725647497`*^9}, {

3.5788043266472826`*^9, 3.5788043820844536`*^9}, {3.578804495594946*^9,

3.5788045037964153`*^9}, {3.578804623522263*^9, 3.5788046283865414`*^9}, {

3.5794097731315403`*^9, 3.579409780970989*^9}, {3.6023853930798798`*^9,

3.6023853945512414`*^9}}],

Cell[BoxData[

RowBox[{

RowBox[{"paramsTurnover", "=",

RowBox[{"{", "\[IndentingNewLine]",

RowBox[{

RowBox[{"vlr", "\[Rule]", "1.8"}], ",", "\[IndentingNewLine]",

RowBox[{"kmlr", "\[Rule]", "0.0011"}], ",", "\[IndentingNewLine]",

RowBox[{"xlr", "\[Rule]", "3"}], ",", "\[IndentingNewLine]",

RowBox[{"vhr", "\[Rule]",

RowBox[{"0.9", "+", "1.8"}]}], ",", "\[IndentingNewLine]",

RowBox[{"kmhr", "\[Rule]", "0.018"}], ",", "\[IndentingNewLine]",

RowBox[{"xhr", "\[Rule]", "0.5"}], ",", "\[IndentingNewLine]",

RowBox[{"vla", "\[Rule]", "0.65"}], " ", ",", "\[IndentingNewLine]",

RowBox[{"kmla", "\[Rule]", "0.00018"}], ",", "\[IndentingNewLine]",

RowBox[{"xla", "\[Rule]", "3"}], ",", "\[IndentingNewLine]",

RowBox[{"vha", "\[Rule]",

RowBox[{"4", "+", "0.6"}]}], ",", "\[IndentingNewLine]",

RowBox[{"kmha", "\[Rule]", "0.155"}], ",", "\[IndentingNewLine]",

RowBox[{"xha", "\[Rule]",

RowBox[{"2", "/", "3"}]}]}], "\[IndentingNewLine]", "}"}]}],

";"}]], "Input",

CellChangeTimes->{{3.5788046304576597`*^9, 3.578804694020295*^9}, {

3.5788048351663685`*^9, 3.5788049000290785`*^9}, {3.578808383557325*^9,

3.5788084129660068`*^9}, {3.5788087609059076`*^9,

3.5788088017552443`*^9}, {3.5788272198937016`*^9,

3.5788272216648026`*^9}, {3.5788295202562747`*^9,

3.5788295208653097`*^9}, {3.5788297015356436`*^9, 3.57882970288072*^9},

3.5838174009976835`*^9, {3.592124450521835*^9, 3.592124483673994*^9}, {

3.5921246467931376`*^9, 3.5921246749210787`*^9}, {3.592124840663449*^9,

3.592124864951426*^9}, {3.592124938742587*^9, 3.5921249639587917`*^9}, {

3.5921365452363377`*^9, 3.5921365493473496`*^9}, {3.5921548025924854`*^9,

3.5921548325283403`*^9}, 3.5921555695005245`*^9, {3.592156415509634*^9,

3.5921564353015046`*^9}, {3.5921964702642174`*^9, 3.5921964775760164`*^9},

3.592196625302813*^9, 3.592196730374816*^9, {3.5921967758460054`*^9,

3.5921967873498373`*^9}, {3.5921977731350937`*^9,

3.5921977756777186`*^9}, {3.592197924797469*^9, 3.5921979320942564`*^9}, {

3.5921982164750056`*^9, 3.592198228043853*^9}, {3.592198408298047*^9,

3.5921984134183145`*^9}, {3.5922006390975637`*^9, 3.592200650410348*^9}, {

3.5922008890810843`*^9, 3.592200905320082*^9}, {3.592201051655093*^9,

3.5922010539106493`*^9}, {3.5922012660378957`*^9,

3.5922012661019115`*^9}, {3.592201643763117*^9, 3.5922016443542624`*^9}, {

3.5922017720502276`*^9, 3.592201773025468*^9}, {3.5922019211854205`*^9,

3.592201921344457*^9}, {3.5922019580486126`*^9, 3.5922019693443923`*^9}, {

3.59220210270422*^9, 3.592202106783224*^9}, 3.5922024551656084`*^9, {

3.592202562844108*^9, 3.5922025669081087`*^9}, {3.5922026478510284`*^9,

3.5922026500115614`*^9}, {3.592204040705658*^9, 3.5922040575368004`*^9}, {

3.592204118816881*^9, 3.592204119152964*^9}, {3.5922042522393517`*^9,

3.5922042581117973`*^9}, {3.592204347951714*^9, 3.5922043651509476`*^9}, {

3.592204889356335*^9, 3.5922049120749273`*^9}, {3.5922050186831627`*^9,

3.592205034282011*^9}, {3.5922061775706625`*^9, 3.592206196433302*^9}, {

3.5922063098902225`*^9, 3.5922063290249324`*^9}, {3.5922064637121954`*^9,

3.5922065175354414`*^9}, {3.592206549087206*^9, 3.5922065507836447`*^9}, {

3.592206616511799*^9, 3.5922066465902014`*^9}, {3.592206778318668*^9,

3.5922067851973543`*^9}, {3.5922068629414873`*^9,

3.5922068911334257`*^9}, {3.5922069324766006`*^9,

3.5922069325406156`*^9}, {3.5922070619484634`*^9,

3.5922070782515173`*^9}, {3.5922172424825935`*^9,

3.5922172722419167`*^9}, {3.5922173292029347`*^9,

3.5922173297620735`*^9}, {3.5922173738099136`*^9, 3.592217373985957*^9}, {

3.5922176445284224`*^9, 3.592217648799472*^9}, {3.5922786631592884`*^9,

3.592278691318218*^9}, 3.592278863556605*^9, {3.5922791419713907`*^9,

3.592279145746315*^9}, {3.5922792190904436`*^9, 3.592279219234498*^9}, {

3.5922796892479177`*^9, 3.5922797077754774`*^9}, {3.5922814303873024`*^9,

3.59228143899442*^9}, {3.592281498194988*^9, 3.59228154492949*^9}, {

3.59228164369773*^9, 3.5922816458402576`*^9}, {3.59228181379246*^9,

3.5922818171032753`*^9}, {3.5922834887232914`*^9, 3.592283607362487*^9}, {

3.5922836984669957`*^9, 3.5922836985470157`*^9}, {3.592283746993938*^9,

3.5922837470419497`*^9}, {3.592284589116404*^9, 3.592284615547908*^9}, {

3.5922846837877016`*^9, 3.592284683883725*^9}, {3.592284863290877*^9,

3.592284865753483*^9}, {3.5922849699941363`*^9, 3.5922849907292395`*^9},

3.5923043469277987`*^9, {3.5923044574219913`*^9, 3.592304458542267*^9},

3.592304497581875*^9, 3.592304653260187*^9, 3.5923047261891346`*^9, {

3.592304814171787*^9, 3.592304823739142*^9}, {3.6023518562169447`*^9,

3.6023518575612764`*^9}, 3.6023519611448617`*^9, {3.602352414181761*^9,

3.6023524485972614`*^9}, {3.6023530312821846`*^9,

3.6023530885123205`*^9}, {3.6023531271048527`*^9, 3.602353132704235*^9}, {

3.6023534519836407`*^9, 3.6023534571349134`*^9}, {3.60238551082096*^9,

3.6023855640831165`*^9}, {3.602405758437728*^9, 3.602405789317355*^9},

3.6024101274498687`*^9, {3.602410161882375*^9, 3.602410178585499*^9}, {

3.6024103039604664`*^9, 3.602410321815877*^9}, {3.6024103829689817`*^9,

3.602410386535862*^9}, {3.602410979844409*^9, 3.6024109873802767`*^9}, {

3.6024117611844*^9, 3.6024117723031454`*^9}, {3.6024118228956413`*^9,

3.6024118437267866`*^9}, {3.602411978718131*^9, 3.6024119787821455`*^9}, {

3.6024136028682933`*^9, 3.602413603283396*^9}, 3.602437072528208*^9,

3.6024371031687765`*^9, {3.6024379290527687`*^9, 3.602437939019232*^9}, {

3.6024398571672373`*^9, 3.6024398573912945`*^9}, {3.6024399131840796`*^9,

3.602439913263093*^9}, {3.6024400359183893`*^9, 3.6024400363344917`*^9}}]

}, Closed]],

Cell[CellGroupData[{

Cell["\[Beta]-cell function", "Subsubsection",

CellChangeTimes->{{3.5788038748664427`*^9, 3.578803884706005*^9}, {

3.578808896527665*^9, 3.578808901598955*^9}, {3.60238541296379*^9,

3.602385424275584*^9}}],

Cell[BoxData[

RowBox[{

RowBox[{"eqsFunction", "=",

RowBox[{"{", "\[IndentingNewLine]",

RowBox[{

RowBox[{

RowBox[{

SuperscriptBox["isc", "\[Prime]",

MultilineFunction->None], "[", "t", "]"}], "\[Equal]",

RowBox[{"taus", " ",

RowBox[{"(",

RowBox[{

RowBox[{"ks", " ",

RowBox[{"(",

RowBox[{"1", "-",

FractionBox[

RowBox[{"vs", " ",

RowBox[{"il1r", "[", "t", "]"}]}],

RowBox[{"kms", "+",

RowBox[{"il1r", "[", "t", "]"}]}]]}], ")"}]}], "-",

RowBox[{"isc", "[", "t", "]"}]}], ")"}]}]}], ",",

"\[IndentingNewLine]",

RowBox[{

RowBox[{

SuperscriptBox["f", "\[Prime]",

MultilineFunction->None], "[", "t", "]"}], "\[Equal]",

RowBox[{"tauf", " ",

RowBox[{"(",

RowBox[{

RowBox[{"kf", "*",

RowBox[{"(",

RowBox[{

RowBox[{"(",

RowBox[{"1", "+",

FractionBox[

RowBox[{"vf", " ",

RowBox[{"il1r", "[", "t", "]"}]}],

RowBox[{"kmf", "+",

RowBox[{"il1r", "[", "t", "]"}]}]]}], ")"}], "*",

RowBox[{"(",

RowBox[{"1", "+",

FractionBox[

RowBox[{"vfg", " ",

RowBox[{

RowBox[{"glucose", "[", "t", "]"}], "^", "xfg"}]}],

RowBox[{

RowBox[{"kmfg", "^", "xfg"}], "+",

RowBox[{

RowBox[{"glucose", "[", "t", "]"}], "^", "xfg"}]}]]}],

")"}]}], ")"}]}], "-",

RowBox[{"f", "[", "t", "]"}]}], ")"}]}]}]}], "\[IndentingNewLine]",

"}"}]}], ";"}]], "Input",

CellChangeTimes->{{3.578214065078707*^9, 3.5782140655727353`*^9}, {

3.578214411430517*^9, 3.5782145509674983`*^9}, {3.5782146131110525`*^9,

3.578214628869954*^9}, {3.5782149214446883`*^9, 3.5782149217637067`*^9}, {

3.5782149572847385`*^9, 3.578214965284196*^9}, 3.578709724283337*^9,

3.5787099902815514`*^9, {3.5787105211259136`*^9, 3.578710560886188*^9}, {

3.578809147903043*^9, 3.5788091633139243`*^9}, 3.578816584077368*^9, {

3.5788929220481634`*^9, 3.5788929244114*^9}, 3.583552110396391*^9, {

3.5835616523451586`*^9, 3.583561675912507*^9}, 3.590132595136281*^9,

3.602385354793422*^9}],

Cell[BoxData[

RowBox[{

RowBox[{"paramsFunction", "=",

RowBox[{"{", "\[IndentingNewLine]",

RowBox[{

RowBox[{"vs", "\[Rule]", "0.7"}], ",", "\[IndentingNewLine]",

RowBox[{"kms", "\[Rule]", "0.021"}], ",", "\[IndentingNewLine]",

RowBox[{"taus", "\[Rule]", "0.5"}], ",", "\[IndentingNewLine]",

RowBox[{"kmf", "\[Rule]", "0.021"}], ",", "\[IndentingNewLine]",

RowBox[{"tauf", "\[Rule]", "0.5"}], ",", "\[IndentingNewLine]",

RowBox[{"vfg", "\[Rule]", "4"}], ",", "\[IndentingNewLine]",

RowBox[{"xfg", "\[Rule]", "4"}], ",", "\[IndentingNewLine]",

RowBox[{"kmfg", "\[Rule]", "9"}], ",", "\[IndentingNewLine]",

RowBox[{"vf", "\[Rule]", "0.4"}]}], "\[IndentingNewLine]", "}"}]}],

";"}]], "Input",

CellChangeTimes->{{3.5788205410696945`*^9, 3.578820558719704*^9}, {

3.578820707356206*^9, 3.578821085373827*^9}, {3.5788211512925973`*^9,

3.5788212099339514`*^9}, {3.578827698110054*^9, 3.5788276984620743`*^9}, {

3.578829685904749*^9, 3.578829698065445*^9}, {3.5829048362025013`*^9,

3.5829048365545216`*^9}, {3.582909392538109*^9, 3.582909392890129*^9}, {

3.5830309382543306`*^9, 3.5830309404934587`*^9}, {3.5830320119917445`*^9,

3.5830320123587656`*^9}, {3.583552114267612*^9, 3.5835521194669094`*^9},

3.583552157083061*^9, {3.583552225097951*^9, 3.5835522285061464`*^9},

3.5835522811461573`*^9, {3.5835523233055687`*^9, 3.5835523465829*^9}, {

3.5835533189615164`*^9, 3.583553319249533*^9}, {3.5835544066977315`*^9,

3.583554427017894*^9}, {3.5835616996558647`*^9, 3.583561709478427*^9},

3.583568153737017*^9, 3.5835750572888775`*^9, {3.5835752288716917`*^9,

3.5835752310788183`*^9}, {3.5892792509401426`*^9, 3.589279255342023*^9},

3.5895134087773085`*^9, 3.5895151847142286`*^9, 3.5895152549858627`*^9, {

3.5895166906702805`*^9, 3.589516691453474*^9}, {3.5895182729296227`*^9,

3.589518275185175*^9}, 3.5895211165385714`*^9, {3.589701649210679*^9,

3.589701649674793*^9}, {3.59013179424225*^9, 3.590131798433282*^9}, {

3.5916036034833555`*^9, 3.5916036063795214`*^9}, {3.591609034270979*^9,

3.591609034610998*^9}, {3.591694547481753*^9, 3.5916945478498526`*^9}, {

3.592032101511262*^9, 3.5920321016152678`*^9}, 3.592114781327015*^9, {

3.5921148384941187`*^9, 3.592114839230301*^9}, {3.5921186025951357`*^9,

3.5921186046746483`*^9}, {3.5921227438460965`*^9,

3.5921227454294863`*^9}, {3.5921250062291937`*^9,

3.5921250062932105`*^9}, {3.5921986309038296`*^9, 3.592198636967331*^9}, {

3.592200513994093*^9, 3.592200517369924*^9}, {3.5922005831291084`*^9,

3.5922005974976444`*^9}, {3.592207105114128*^9, 3.5922071080578527`*^9}, {

3.592285081607604*^9, 3.5922850868719006`*^9}, {3.5922852591102877`*^9,

3.5922852611897993`*^9}, 3.592493564959953*^9, {3.5924975148039474`*^9,

3.592497517443598*^9}, {3.592497644274811*^9, 3.5924976451390233`*^9}, {

3.5924999619865746`*^9, 3.5924999644181733`*^9}, {3.5925544360443745`*^9,

3.5925544379158344`*^9}, {3.5925546067150035`*^9, 3.5925546075302033`*^9},

3.6023853093343267`*^9, {3.602385869602579*^9, 3.6023858903537045`*^9}}]

}, Closed]]

}, Closed]]

}, Closed]],

Cell[CellGroupData[{

Cell["Non-diseased steady state", "Section",

CellChangeTimes->{{3.578802666315317*^9, 3.578802667051359*^9}, {

3.578823818230138*^9, 3.578823833797028*^9}, {3.6023856403059435`*^9,

3.6023856472976713`*^9}}],

Cell[BoxData[

RowBox[{

RowBox[{"ndSteadyState", "=",

RowBox[{"{", "\[IndentingNewLine]",

RowBox[{

RowBox[{

RowBox[{"glucose", "[", "t", "]"}], "\[Rule]", "5"}],

RowBox[{"(*", "mM", "*)"}], ",", "\[IndentingNewLine]",

RowBox[{

RowBox[{"insulin", "[", "t", "]"}], "\[Rule]", "50"}],

RowBox[{"(*", "pM", "*)"}], ",", "\[IndentingNewLine]",

RowBox[{

RowBox[{"proinsulin", "[", "t", "]"}], "\[Rule]", "6.5"}],

RowBox[{"(*", "pM", "*)"}], ",", "\[IndentingNewLine]",

RowBox[{

RowBox[{"b", "[", "t", "]"}], "\[Rule]", "100"}],

RowBox[{"(*", "%", "*)"}], ",", "\[IndentingNewLine]",

RowBox[{

RowBox[{"apoptosis", "[", "t", "]"}], "\[Rule]", "0.000274"}], ",",

"\[IndentingNewLine]",

RowBox[{

RowBox[{"il1b", "[", "t", "]"}], "\[Rule]",

RowBox[{"0.0005", " ", "*", "100"}]}],

RowBox[{"(*",

RowBox[{"ng", "/", "ml"}], "*)"}], ",", "\[IndentingNewLine]",

RowBox[{

RowBox[{"il1ra", "[", "t", "]"}], "\[Rule]",

RowBox[{"0.25", "*", "100"}]}]}],

RowBox[{"(*",

RowBox[{"ng", "/", "ml"}], "*)"}], "\[IndentingNewLine]", "}"}]}],

";"}]], "Input",

CellChangeTimes->{{3.5788241318370748`*^9, 3.578824135636292*^9}, {

3.5788242272515326`*^9, 3.5788242420683794`*^9}, {3.5788272408308992`*^9,

3.5788272548957033`*^9}, 3.579251236967177*^9, 3.582883789709711*^9,

3.5829027323931704`*^9, {3.58290277540063*^9, 3.582902776423689*^9}, {

3.5829028718471465`*^9, 3.582902871975154*^9}, {3.5829029335586767`*^9,

3.582902933670683*^9}, {3.582903038646687*^9, 3.5829030387746944`*^9}, {

3.58290307821395*^9, 3.58290308222918*^9}, {3.5829032706449566`*^9,

3.5829032731561003`*^9}, 3.582939171399362*^9, {3.5829694916871047`*^9,

3.5829694965635924`*^9}, {3.5830320576373553`*^9,

3.5830320715891533`*^9}, {3.583033380172*^9, 3.58303338244413*^9},

3.5830339917359796`*^9, {3.5835617460545187`*^9, 3.583561746262531*^9}, {

3.583578825084585*^9, 3.58357882516459*^9}, {3.5835788598525734`*^9,

3.5835788641878214`*^9}, 3.5835806057914357`*^9, {3.583592864457591*^9,

3.5835928671137433`*^9}, 3.589714140390457*^9, 3.590130426364111*^9, {

3.590132712475159*^9, 3.5901327126031895`*^9}, {3.5901327813391547`*^9,

3.590132781435178*^9}, 3.5902286613937373`*^9, 3.590228987359957*^9,

3.590229843801726*^9, 3.5902304121008177`*^9, 3.5914958153303657`*^9,

3.591496333673017*^9, 3.5914966389179077`*^9, 3.5915043382716303`*^9, {

3.591953270792592*^9, 3.5919532714147453`*^9}, {3.592037093503787*^9,

3.5920370949458694`*^9}, 3.5920371320289907`*^9, {3.59203865867731*^9,

3.592038661251457*^9}, {3.592113373656784*^9, 3.5921133760893836`*^9},

3.592214436919079*^9, {3.5923016194259486`*^9, 3.592301621729515*^9}, {

3.6023856956836214`*^9, 3.6023856978271513`*^9}, {3.602385747778488*^9,

3.602385752386627*^9}, {3.602385834498909*^9, 3.6023858601462436`*^9}, {

3.602400772517212*^9, 3.602400776484191*^9}}],

Cell[BoxData[

RowBox[{

RowBox[{"il1rSteadyState", "=",

RowBox[{"First", "[",

RowBox[{"Solve", "[",

RowBox[{"eqModulation", "/.",

RowBox[{"Join", "[",

RowBox[{"paramsModulation", ",", "ndSteadyState"}], "]"}]}], "]"}],

"]"}]}], ";"}]], "Input",

CellChangeTimes->{{3.602400732979447*^9, 3.6024008092362814`*^9},

3.602939664507145*^9}],

Cell[BoxData[

RowBox[{

RowBox[{"solSteadyState", "=",

RowBox[{"First", "[",

RowBox[{"Solve", "[",

RowBox[{

RowBox[{

RowBox[{"Join", "[",

RowBox[{

"eqsTurnover", ",", "eqsGlucoseInsulin", ",", "eqsFunction", ",",

"eqsHbA1C"}], "]"}], "/.",

RowBox[{"Join", "[",

RowBox[{

RowBox[{"{",

RowBox[{

RowBox[{

RowBox[{"_", "'"}], "[", "t", "]"}], "\[Rule]", "0"}], "}"}], ",",

"paramsTurnover", ",", "paramsFunction", ",",

"paramsGlucoseInsulin", ",", "paramsHbA1C", ",", "ndSteadyState",

",", "il1rSteadyState"}], "]"}]}], ",",

RowBox[{"{",

RowBox[{"ka", ",", "kr", ",", "kf", ",", "ks", ",", "Tgl", ",",

RowBox[{"replication", "[", "t", "]"}], ",",

RowBox[{"f", "[", "t", "]"}], ",",

RowBox[{"isc", "[", "t", "]"}], ",",

RowBox[{"a1c1", "[", "t", "]"}], ",",

RowBox[{"a1c2", "[", "t", "]"}], ",",

RowBox[{"a1c3", "[", "t", "]"}], ",",

RowBox[{"a1c4", "[", "t", "]"}], ",",

RowBox[{"a1c5", "[", "t", "]"}], ",",

RowBox[{"a1c6", "[", "t", "]"}], ",",

RowBox[{"a1c7", "[", "t", "]"}], ",",

RowBox[{"a1c8", "[", "t", "]"}], ",",

RowBox[{"a1c9", "[", "t", "]"}], ",",

RowBox[{"a1c10", "[", "t", "]"}], ",",

RowBox[{"a1c11", "[", "t", "]"}], ",",

RowBox[{"a1c12", "[", "t", "]"}], ",",

RowBox[{"rbc1", "[", "t", "]"}], ",",

RowBox[{"rbc2", "[", "t", "]"}], ",",

RowBox[{"rbc3", "[", "t", "]"}], ",",

RowBox[{"rbc4", "[", "t", "]"}], ",",

RowBox[{"rbc5", "[", "t", "]"}], ",",

RowBox[{"rbc6", "[", "t", "]"}], ",",

RowBox[{"rbc7", "[", "t", "]"}], ",",

RowBox[{"rbc8", "[", "t", "]"}], ",",

RowBox[{"rbc9", "[", "t", "]"}], ",",

RowBox[{"rbc10", "[", "t", "]"}], ",",

RowBox[{"rbc11", "[", "t", "]"}], ",",

RowBox[{"rbc12", "[", "t", "]"}], ",",

RowBox[{"hba1c", "[", "t", "]"}]}], "}"}]}], "]"}], "]"}]}],

";"}]], "Input",

CellChangeTimes->{{3.602400835986889*^9, 3.602400903491562*^9}, {

3.6024160814129543`*^9, 3.6024160885167103`*^9}, {3.602419413311929*^9,

3.602419424751763*^9}, {3.602419591422923*^9, 3.6024195926252203`*^9}, {

3.6024220269114857`*^9, 3.6024222045283566`*^9}, 3.602422237391474*^9, {

3.6024229469717407`*^9, 3.602422955803925*^9}}],

Cell[BoxData[

RowBox[{

RowBox[{"paramsCalc", "=",

RowBox[{"solSteadyState", "[",

RowBox[{"[",

RowBox[{"1", ";;", "5"}], "]"}], "]"}]}], ";"}]], "Input",

CellChangeTimes->{{3.6024232745466504`*^9, 3.602423286822682*^9}}]

}, Closed]],

Cell[CellGroupData[{

Cell["Diseased state", "Section",

CellChangeTimes->{{3.578802666315317*^9, 3.578802667051359*^9}, {

3.578823818230138*^9, 3.578823833797028*^9}, {3.6023856403059435`*^9,

3.6023856472976713`*^9}, {3.6024233266775274`*^9, 3.602423330245408*^9}}],

Cell[BoxData[

RowBox[{

RowBox[{"dState", "=",

RowBox[{"{", "\[IndentingNewLine]",

RowBox[{

RowBox[{

RowBox[{"glucose", "[", "t", "]"}], "\[Rule]", "10.8"}],

RowBox[{"(*", "mM", "*)"}], ",", "\[IndentingNewLine]",

RowBox[{

RowBox[{"insulin", "[", "t", "]"}], "\[Rule]", "100"}],

RowBox[{"(*", "pM", "*)"}], ",", "\[IndentingNewLine]",

RowBox[{

RowBox[{"b", "[", "t", "]"}], "\[Rule]", "40"}], ",",

"\[IndentingNewLine]",

RowBox[{

RowBox[{"il1ra", "[", "t", "]"}], "\[Rule]",

RowBox[{"0.25", "*", "100", "*", "1.6"}]}]}],

RowBox[{"(*",

RowBox[{"ng", "/", "ml"}], "*)"}], "\[IndentingNewLine]", "}"}]}],

";"}]], "Input",

CellChangeTimes->{{3.6024233333451743`*^9, 3.6024233573831105`*^9}, {

3.602423626949693*^9, 3.6024236303885427`*^9}, {3.602423688548908*^9,

3.6024237027414136`*^9}, {3.602438041867634*^9, 3.602438069354423*^9}}],

Cell[BoxData[

RowBox[{

RowBox[{"solDiseasedState", "=",

RowBox[{"First", "[",

RowBox[{"Solve", "[",

RowBox[{

RowBox[{

RowBox[{"Join", "[",

RowBox[{

"eqsGlucoseInsulin", ",", "eqsHbA1C", ",", "eqsFunction", ",",

"eqModulation"}], "]"}], "/.",

RowBox[{"DeleteCases", "[",

RowBox[{

RowBox[{"Join", "[",

RowBox[{

RowBox[{"{",

RowBox[{

RowBox[{

RowBox[{"_", "'"}], "[", "t", "]"}], "\[Rule]", "0"}], "}"}],

",", "dState", ",", "paramsCalc", ",", "paramsFunction", ",",

"paramsGlucoseInsulin", ",", "paramsHbA1C", ",",

"paramsModulation"}], "]"}], ",",

RowBox[{

RowBox[{"(", "Kxgi", ")"}], "\[Rule]", "_"}]}], "]"}]}], ",",

RowBox[{"{",

RowBox[{"Kxgi", ",",

RowBox[{"il1b", "[", "t", "]"}], ",",

RowBox[{"proinsulin", "[", "t", "]"}], ",",

RowBox[{"f", "[", "t", "]"}], ",",

RowBox[{"isc", "[", "t", "]"}], ",",

RowBox[{"il1r", "[", "t", "]"}], ",",

RowBox[{"a1c1", "[", "t", "]"}], ",",

RowBox[{"a1c2", "[", "t", "]"}], ",",

RowBox[{"a1c3", "[", "t", "]"}], ",",

RowBox[{"a1c4", "[", "t", "]"}], ",",

RowBox[{"a1c5", "[", "t", "]"}], ",",

RowBox[{"a1c6", "[", "t", "]"}], ",",

RowBox[{"a1c7", "[", "t", "]"}], ",",

RowBox[{"a1c8", "[", "t", "]"}], ",",

RowBox[{"a1c9", "[", "t", "]"}], ",",

RowBox[{"a1c10", "[", "t", "]"}], ",",

RowBox[{"a1c11", "[", "t", "]"}], ",",

RowBox[{"a1c12", "[", "t", "]"}], ",",

RowBox[{"rbc1", "[", "t", "]"}], ",",

RowBox[{"rbc2", "[", "t", "]"}], ",",

RowBox[{"rbc3", "[", "t", "]"}], ",",

RowBox[{"rbc4", "[", "t", "]"}], ",",

RowBox[{"rbc5", "[", "t", "]"}], ",",

RowBox[{"rbc6", "[", "t", "]"}], ",",

RowBox[{"rbc7", "[", "t", "]"}], ",",

RowBox[{"rbc8", "[", "t", "]"}], ",",

RowBox[{"rbc9", "[", "t", "]"}], ",",

RowBox[{"rbc10", "[", "t", "]"}], ",",

RowBox[{"rbc11", "[", "t", "]"}], ",",

RowBox[{"rbc12", "[", "t", "]"}], ",",

RowBox[{"hba1c", "[", "t", "]"}]}], "}"}]}], "]"}], "]"}]}],

";"}]], "Input",

InitializationCell->True,

CellChangeTimes->{{3.578903885630949*^9, 3.578903909474333*^9}, {

3.578905587828147*^9, 3.578905628903254*^9}, 3.5789061705554137`*^9, {

3.57890903558022*^9, 3.57890903654722*^9}, {3.5829711077717066`*^9,

3.5829711247244015`*^9}, {3.582971180275956*^9, 3.5829712464945774`*^9}, {

3.5830322386317077`*^9, 3.5830322417198844`*^9}, {3.5835683287800293`*^9,

3.5835683421237926`*^9}, {3.583568375003673*^9, 3.5835684084275846`*^9}, {

3.591693661719509*^9, 3.591693692920187*^9}, {3.591693771239461*^9,

3.591693939166604*^9}, {3.591695554703062*^9, 3.5916955949409647`*^9}, {

3.591697364689535*^9, 3.591697373088602*^9}, {3.591698455865117*^9,

3.5916984738975544`*^9}, 3.601958410512038*^9, {3.6024237239106426`*^9,

3.6024237533399115`*^9}, {3.6024238339578238`*^9, 3.60242385325359*^9}, {

3.6024239800359054`*^9, 3.602424015298615*^9}, {3.6024296489281144`*^9,

3.6024296858552356`*^9}, {3.602429716256745*^9, 3.602429727647558*^9}, {

3.6024297951672354`*^9, 3.6024297952792635`*^9}, {3.6024299275659375`*^9,

3.602429959806901*^9}, {3.6024380573414555`*^9, 3.6024380577285514`*^9},

3.6029394147194476`*^9}],

Cell[BoxData[

RowBox[{

RowBox[{"dParamsCalc", "=",

RowBox[{"{",

RowBox[{"solDiseasedState", "[",

RowBox[{"[", "1", "]"}], "]"}], "}"}]}], ";"}]], "Input",

CellChangeTimes->{{3.6024316292802596`*^9, 3.602431674226361*^9}}],

Cell[BoxData[

RowBox[{

RowBox[{"dStateCalc", "=",

RowBox[{"solDiseasedState", "[",

RowBox[{"[",

RowBox[{"2", ";;"}], "]"}], "]"}]}], ";"}]], "Input",

CellChangeTimes->{{3.6024316813911304`*^9, 3.602431696658901*^9}}]

}, Closed]],

Cell[CellGroupData[{

Cell["IL-1\[Beta], IL-1Ra, and anakinra dynamics", "Section",

CellChangeTimes->{{3.6024300172760963`*^9, 3.60243003036333*^9}}],

Cell[BoxData[

RowBox[{

RowBox[{"eqsAnakinra", "=",

RowBox[{"{", "\[IndentingNewLine]",

RowBox[{

RowBox[{

RowBox[{

RowBox[{"anakinraDose", "'"}], "[", "t", "]"}], "\[Equal]",

RowBox[{

RowBox[{"-", "kab"}], "*",

RowBox[{"anakinraDose", "[", "t", "]"}]}]}], ",",

"\[IndentingNewLine]",

RowBox[{

RowBox[{

RowBox[{"anakinraPlasma", "'"}], "[", "t", "]"}], "\[Equal]",

RowBox[{

FractionBox[

RowBox[{"kab", "*",

RowBox[{"anakinraDose", "[", "t", "]"}]}], "Vp"], "-",

FractionBox[

RowBox[{"Cl", "*",

RowBox[{"anakinraPlasma", "[", "t", "]"}]}], "Vp"]}]}], ",",

RowBox[{"WhenEvent", "[",

RowBox[{

RowBox[{"And", "[",

RowBox[{

RowBox[{

RowBox[{"Mod", "[",

RowBox[{"t", ",", "1"}], "]"}], "\[Equal]", "0"}], ",",

RowBox[{"t", "<", "92"}]}], "]"}], ",", "\[IndentingNewLine]",

RowBox[{

RowBox[{"anakinraDose", "[", "t", "]"}], "\[Rule]",

RowBox[{

RowBox[{"anakinraDose", "[", "t", "]"}], "+", "100"}]}]}], "]"}],

",", "\[IndentingNewLine]",

RowBox[{

RowBox[{"anakinraDose", "[", "0", "]"}], "\[Equal]", "100"}], ",",

"\[IndentingNewLine]",

RowBox[{

RowBox[{"anakinraPlasma", "[", "0", "]"}], "\[Equal]", "0"}]}], "}"}]}],

";"}]], "Input",

CellChangeTimes->{{3.6024300601656895`*^9, 3.6024301015649157`*^9},

3.602430175244114*^9, {3.6024303219643536`*^9, 3.6024303507794714`*^9}, {

3.6024333092892194`*^9, 3.6024333096253023`*^9}, {3.602939474215143*^9,

3.602939503438361*^9}}],

Cell[BoxData[

RowBox[{

RowBox[{"paramsAnakinra", "=",

RowBox[{"{", "\[IndentingNewLine]",

RowBox[{

RowBox[{"kab", "\[Rule]", "3.94"}], ",", "\[IndentingNewLine]",

RowBox[{"Cl", "\[Rule]", "432"}], ",", "\[IndentingNewLine]",

RowBox[{"Vp", "\[Rule]", "48"}]}], "\[IndentingNewLine]", "}"}]}],

";"}]], "Input",

CellChangeTimes->{{3.6024301676242323`*^9, 3.6024302129714327`*^9}, {

3.6024302843310585`*^9, 3.6024302908106585`*^9}, 3.602430439050275*^9}],

Cell[BoxData[

RowBox[{

RowBox[{"anakinra", "=",

RowBox[{"anakinraPlasma", "/.",

RowBox[{"First", "[",

RowBox[{"NDSolve", "[",

RowBox[{

RowBox[{"eqsAnakinra", "/.", "paramsAnakinra"}], ",",

RowBox[{"{", "anakinraPlasma", "}"}], ",",

RowBox[{"{",

RowBox[{"t", ",", "0", ",", "10000"}], "}"}]}], "]"}], "]"}]}]}],

";"}]], "Input",

InitializationCell->True,

CellChangeTimes->{{3.578658432191597*^9, 3.5786584804853597`*^9}, {

3.5786710127961664`*^9, 3.578671275339183*^9}, {3.578671870391218*^9,

3.578671874535455*^9}, {3.578671967302761*^9, 3.5786719960224037`*^9},

3.5787118506069555`*^9, {3.5787177958440037`*^9, 3.5787178436187363`*^9}, {

3.57872745961574*^9, 3.5787274721754584`*^9}, {3.5787276186378355`*^9,

3.5787276187018394`*^9}, {3.5787348261400814`*^9,

3.5787348264440985`*^9}, {3.5787349073557262`*^9,

3.5787349076747446`*^9}, {3.5787357152059326`*^9, 3.578735715877971*^9},

3.5787364297288013`*^9, {3.5787409813121367`*^9, 3.578740981904171*^9}, {

3.578741074879489*^9, 3.5787410752635107`*^9}, {3.5787427873154345`*^9,

3.578742788883524*^9}, {3.578894556936655*^9, 3.5788945589228535`*^9}, {

3.578894665609521*^9, 3.5788947178517447`*^9}, {3.595650327859852*^9,

3.5956503980988646`*^9}, {3.595650444211031*^9, 3.595650458461484*^9},

3.6024300589984007`*^9, {3.6024301090037527`*^9, 3.6024301377748594`*^9}, {

3.602430373627115*^9, 3.602430409050865*^9}, {3.6024305004894495`*^9,

3.60243054757708*^9}, 3.6029395180599732`*^9}],

Cell[BoxData[

RowBox[{

RowBox[{"eqModulationAnakinra", "=", "\[IndentingNewLine]",

RowBox[{"{",

RowBox[{

RowBox[{"il1r", "[", "t", "]"}], "\[Equal]",

FractionBox[

RowBox[{"il1b", "[", "t", "]"}],

RowBox[{

RowBox[{"il1b", "[", "t", "]"}], "+",

RowBox[{"km", " ",

RowBox[{"(",

RowBox[{"1", "+",

FractionBox[

RowBox[{"(",

RowBox[{

RowBox[{"il1ra", "[", "t", "]"}], "+",

RowBox[{"1000", "*",

RowBox[{"anakinra", "[", "t", "]"}]}]}], ")"}], "ki"]}],

")"}]}]}]]}], "}"}]}], ";"}]], "Input",

CellChangeTimes->{

3.602430629544326*^9, {3.6024307254320107`*^9, 3.6024307672893486`*^9},

3.602430805784857*^9}],

Cell[BoxData[

RowBox[{

RowBox[{"eqIl1bTreatment", "=",

RowBox[{"{", "\[IndentingNewLine]",

RowBox[{

RowBox[{

RowBox[{"il1b", "'"}], "[", "t", "]"}], "==",

RowBox[{"If", "[",

RowBox[{

RowBox[{"0", "<", "t", "\[LessEqual]", "91"}], ",",

RowBox[{

RowBox[{"(",

RowBox[{"il1bH", "-",

RowBox[{"il1b", "[", "t", "]"}]}], ")"}], "*", "k1"}], ",",

RowBox[{"k2", "*",

RowBox[{"(",

RowBox[{

RowBox[{"(",

RowBox[{"il1b0", "+",

RowBox[{"kplacebo", "*", "t"}]}], ")"}], "-",

RowBox[{"il1b", "[", "t", "]"}]}], ")"}]}]}], "]"}]}],

"\[IndentingNewLine]", "}"}]}], ";"}]], "Input",

CellChangeTimes->{{3.6024308587749453`*^9, 3.602430892817354*^9}, {

3.602431004358905*^9, 3.6024310170310354`*^9}, {3.6024311280854654`*^9,

3.602431164725515*^9}, {3.602431321749299*^9, 3.6024313243409386`*^9},

3.6024314843554626`*^9}],

Cell[BoxData[

RowBox[{

RowBox[{"eqIl1bPlacebo", "=",

RowBox[{"{", "\[IndentingNewLine]",

RowBox[{

RowBox[{

RowBox[{"il1b", "'"}], "[", "t", "]"}], "\[Equal]", "kplacebo"}],

"\[IndentingNewLine]", "}"}]}], ";"}]], "Input",

CellChangeTimes->{{3.6024312457785344`*^9, 3.6024312814763536`*^9},

3.602431435683441*^9}],

Cell[BoxData[

RowBox[{

RowBox[{"paramsIl1b", "=",

RowBox[{"{", "\[IndentingNewLine]",

RowBox[{

RowBox[{"il1bH", "\[Rule]",

RowBox[{"0.0005", " ", "*", "100"}]}], ",", "\[IndentingNewLine]",

RowBox[{"il1b0", "\[Rule]", "4.834255667607401`"}], ",",

"\[IndentingNewLine]",

RowBox[{"kplacebo", "\[Rule]", "0.00137"}], ",", "\[IndentingNewLine]",

RowBox[{"k1", "\[Rule]", "0.2"}], ",", "\[IndentingNewLine]",

RowBox[{"k2", "\[Rule]", "0.0025"}]}], "\[IndentingNewLine]", "}"}]}],

";"}]], "Input",

CellChangeTimes->{{3.6024312842740445`*^9, 3.6024313892839804`*^9}, {

3.602431420691738*^9, 3.6024314285956907`*^9}, 3.6024384053695135`*^9}],

Cell[BoxData[

RowBox[{

RowBox[{"eqIl1ra", "=",

RowBox[{"{", "\[IndentingNewLine]",

RowBox[{

RowBox[{

RowBox[{"il1ra", "'"}], "[", "t", "]"}], "\[Equal]", "0"}],

"\[IndentingNewLine]", "}"}]}], ";"}]], "Input",

CellChangeTimes->{{3.602431431711461*^9, 3.602431431779476*^9}, {

3.6024314875392494`*^9, 3.602431506099834*^9}}]

}, Closed]],

Cell[CellGroupData[{

Cell["Summary", "Section",

CellChangeTimes->{{3.6024317394564724`*^9, 3.602431741232911*^9}}],

Cell[BoxData[

RowBox[{

RowBox[{"eqsSysTreatment", "=",

RowBox[{"Join", "[",

RowBox[{

"eqsGlucoseInsulin", ",", "eqsHbA1C", ",", "eqsFunction", ",",

"eqsTurnover", ",", "eqModulationAnakinra", ",", "eqIl1bTreatment", ",",

"eqIl1ra"}], "]"}]}], ";"}]], "Input",

CellChangeTimes->{{3.602431747258401*^9, 3.6024318193932166`*^9}}],

Cell[BoxData[

RowBox[{

RowBox[{"eqsSysPlacebo", "=",

RowBox[{"Join", "[",

RowBox[{

"eqsGlucoseInsulin", ",", "eqsHbA1C", ",", "eqsFunction", ",",

"eqsTurnover", ",", "eqModulation", ",", "eqIl1bPlacebo", ",",

"eqIl1ra"}], "]"}]}], ";"}]], "Input",

CellChangeTimes->{{3.602431820539501*^9, 3.6024318485614214`*^9}}],

Cell[BoxData[

RowBox[{

RowBox[{"params", "=",

RowBox[{"Join", "[",

RowBox[{

RowBox[{"DeleteCases", "[",

RowBox[{"paramsGlucoseInsulin", ",",

RowBox[{"Kxgi", "\[Rule]", "_"}]}], "]"}], ",", "paramsHbA1C", ",",

"paramsFunction", ",", "paramsTurnover", ",", "paramsModulation", ",",

"paramsCalc", ",", "dParamsCalc", ",", "paramsIl1b"}], "]"}]}],

";"}]], "Input",

CellChangeTimes->{{3.6024318519942703`*^9, 3.6024319409292355`*^9}, {

3.602432177887765*^9, 3.6024321902558193`*^9}, {3.6024322340326324`*^9,

3.602432236975359*^9}}],

Cell[BoxData[

RowBox[{

RowBox[{"initConds", "=",

RowBox[{

RowBox[{"Equal", "@@@",

RowBox[{"Join", "[",

RowBox[{"dState", ",", "dStateCalc"}], "]"}]}], "/.",

RowBox[{"{",

RowBox[{"t", "\[Rule]", "0"}], "}"}]}]}], ";"}]], "Input",

CellChangeTimes->{{3.602431946507614*^9, 3.602431997712261*^9}, {

3.6024320981600723`*^9, 3.6024321059519963`*^9}}],

Cell[CellGroupData[{

Cell["Treatment model", "Subsection",

CellChangeTimes->{{3.6029395611626205`*^9, 3.6029395674181657`*^9}}],

Cell[CellGroupData[{

Cell[BoxData[

RowBox[{"eqsSysTreatment", "//", "TableForm"}]], "Input",

CellChangeTimes->{{3.6029395746769576`*^9, 3.602939583390109*^9}}],

Cell[BoxData[

TagBox[

TagBox[GridBox[{

{

RowBox[{

RowBox[{"glucose", "[", "t", "]"}], "\[Equal]",

FractionBox["Tgl",

RowBox[{"Kxg", "+",

RowBox[{"Kxgi", " ",

RowBox[{"insulin", "[", "t", "]"}]}], "+",

RowBox[{"0.1`", " ", "Kxgi", " ",

RowBox[{"proinsulin", "[", "t", "]"}]}]}]]}]},

{

RowBox[{

RowBox[{"insulin", "[", "t", "]"}], "\[Equal]",

FractionBox[

RowBox[{

RowBox[{"b", "[", "t", "]"}], " ",

SuperscriptBox[

RowBox[{"(",

FractionBox[

RowBox[{"glucose", "[", "t", "]"}], "Gh"], ")"}], "vh"], " ",

RowBox[{"isc", "[", "t", "]"}]}],

RowBox[{"Kxi", " ",

RowBox[{"(",

RowBox[{"1", "+",

SuperscriptBox[

RowBox[{"(",

FractionBox[

RowBox[{"glucose", "[", "t", "]"}], "Gh"], ")"}], "vh"]}],

")"}]}]]}]},

{

RowBox[{

RowBox[{"proinsulin", "[", "t", "]"}], "\[Equal]",

FractionBox[

RowBox[{"10.`", " ",

RowBox[{"b", "[", "t", "]"}], " ",

RowBox[{"f", "[", "t", "]"}], " ",

SuperscriptBox[

RowBox[{"(",

FractionBox[

RowBox[{"glucose", "[", "t", "]"}], "Gh"], ")"}], "vh"], " ",

RowBox[{"isc", "[", "t", "]"}]}],

RowBox[{"Kxi", " ",

RowBox[{"(",

RowBox[{"1", "+",

SuperscriptBox[

RowBox[{"(",

FractionBox[

RowBox[{"glucose", "[", "t", "]"}], "Gh"], ")"}], "vh"]}],

")"}]}]]}]},

{

RowBox[{

RowBox[{"hba1c", "[", "t", "]"}], "\[Equal]",

FractionBox[

RowBox[{"100", " ",

RowBox[{"(",

RowBox[{

RowBox[{"a1c1", "[", "t", "]"}], "+",

RowBox[{"a1c10", "[", "t", "]"}], "+",

RowBox[{"a1c11", "[", "t", "]"}], "+",

RowBox[{"a1c12", "[", "t", "]"}], "+",

RowBox[{"a1c2", "[", "t", "]"}], "+",

RowBox[{"a1c3", "[", "t", "]"}], "+",

RowBox[{"a1c4", "[", "t", "]"}], "+",

RowBox[{"a1c5", "[", "t", "]"}], "+",

RowBox[{"a1c6", "[", "t", "]"}], "+",

RowBox[{"a1c7", "[", "t", "]"}], "+",

RowBox[{"a1c8", "[", "t", "]"}], "+",

RowBox[{"a1c9", "[", "t", "]"}]}], ")"}]}],

RowBox[{

RowBox[{"a1c1", "[", "t", "]"}], "+",

RowBox[{"a1c10", "[", "t", "]"}], "+",

RowBox[{"a1c11", "[", "t", "]"}], "+",

RowBox[{"a1c12", "[", "t", "]"}], "+",

RowBox[{"a1c2", "[", "t", "]"}], "+",

RowBox[{"a1c3", "[", "t", "]"}], "+",

RowBox[{"a1c4", "[", "t", "]"}], "+",

RowBox[{"a1c5", "[", "t", "]"}], "+",

RowBox[{"a1c6", "[", "t", "]"}], "+",

RowBox[{"a1c7", "[", "t", "]"}], "+",

RowBox[{"a1c8", "[", "t", "]"}], "+",

RowBox[{"a1c9", "[", "t", "]"}], "+",

RowBox[{"rbc1", "[", "t", "]"}], "+",

RowBox[{"rbc10", "[", "t", "]"}], "+",

RowBox[{"rbc11", "[", "t", "]"}], "+",

RowBox[{"rbc12", "[", "t", "]"}], "+",

RowBox[{"rbc2", "[", "t", "]"}], "+",

RowBox[{"rbc3", "[", "t", "]"}], "+",

RowBox[{"rbc4", "[", "t", "]"}], "+",

RowBox[{"rbc5", "[", "t", "]"}], "+",

RowBox[{"rbc6", "[", "t", "]"}], "+",

RowBox[{"rbc7", "[", "t", "]"}], "+",

RowBox[{"rbc8", "[", "t", "]"}], "+",

RowBox[{"rbc9", "[", "t", "]"}]}]]}]},

{

RowBox[{

RowBox[{

SuperscriptBox["rbc1", "\[Prime]",

MultilineFunction->None], "[", "t", "]"}], "\[Equal]",

RowBox[{"Kin", "-",

RowBox[{"Ktr", " ",

RowBox[{"rbc1", "[", "t", "]"}]}], "-",

RowBox[{"Kglucose", " ",

SuperscriptBox[

RowBox[{"glucose", "[", "t", "]"}], "lambda"], " ",

RowBox[{"rbc1", "[", "t", "]"}]}]}]}]},

{

RowBox[{

RowBox[{

SuperscriptBox["rbc2", "\[Prime]",

MultilineFunction->None], "[", "t", "]"}], "\[Equal]",

RowBox[{

RowBox[{"Ktr", " ",

RowBox[{"rbc1", "[", "t", "]"}]}], "-",

RowBox[{"Ktr", " ",

RowBox[{"rbc2", "[", "t", "]"}]}], "-",

RowBox[{"Kglucose", " ",

SuperscriptBox[

RowBox[{"glucose", "[", "t", "]"}], "lambda"], " ",

RowBox[{"rbc2", "[", "t", "]"}]}]}]}]},

{

RowBox[{

RowBox[{

SuperscriptBox["rbc3", "\[Prime]",

MultilineFunction->None], "[", "t", "]"}], "\[Equal]",

RowBox[{

RowBox[{"Ktr", " ",

RowBox[{"rbc2", "[", "t", "]"}]}], "-",

RowBox[{"Ktr", " ",

RowBox[{"rbc3", "[", "t", "]"}]}], "-",

RowBox[{"Kglucose", " ",

SuperscriptBox[

RowBox[{"glucose", "[", "t", "]"}], "lambda"], " ",

RowBox[{"rbc3", "[", "t", "]"}]}]}]}]},

{

RowBox[{

RowBox[{

SuperscriptBox["rbc4", "\[Prime]",

MultilineFunction->None], "[", "t", "]"}], "\[Equal]",

RowBox[{

RowBox[{"Ktr", " ",

RowBox[{"rbc3", "[", "t", "]"}]}], "-",

RowBox[{"Ktr", " ",

RowBox[{"rbc4", "[", "t", "]"}]}], "-",

RowBox[{"Kglucose", " ",

SuperscriptBox[

RowBox[{"glucose", "[", "t", "]"}], "lambda"], " ",

RowBox[{"rbc4", "[", "t", "]"}]}]}]}]},

{

RowBox[{

RowBox[{

SuperscriptBox["rbc5", "\[Prime]",

MultilineFunction->None], "[", "t", "]"}], "\[Equal]",

RowBox[{

RowBox[{"Ktr", " ",

RowBox[{"rbc4", "[", "t", "]"}]}], "-",

RowBox[{"Ktr", " ",

RowBox[{"rbc5", "[", "t", "]"}]}], "-",

RowBox[{"Kglucose", " ",

SuperscriptBox[

RowBox[{"glucose", "[", "t", "]"}], "lambda"], " ",

RowBox[{"rbc5", "[", "t", "]"}]}]}]}]},

{

RowBox[{

RowBox[{

SuperscriptBox["rbc6", "\[Prime]",

MultilineFunction->None], "[", "t", "]"}], "\[Equal]",

RowBox[{

RowBox[{"Ktr", " ",

RowBox[{"rbc5", "[", "t", "]"}]}], "-",

RowBox[{"Ktr", " ",

RowBox[{"rbc6", "[", "t", "]"}]}], "-",

RowBox[{"Kglucose", " ",

SuperscriptBox[

RowBox[{"glucose", "[", "t", "]"}], "lambda"], " ",

RowBox[{"rbc6", "[", "t", "]"}]}]}]}]},

{

RowBox[{

RowBox[{

SuperscriptBox["rbc7", "\[Prime]",

MultilineFunction->None], "[", "t", "]"}], "\[Equal]",

RowBox[{

RowBox[{"Ktr", " ",

RowBox[{"rbc6", "[", "t", "]"}]}], "-",

RowBox[{"Ktr", " ",

RowBox[{"rbc7", "[", "t", "]"}]}], "-",

RowBox[{"Kglucose", " ",

SuperscriptBox[

RowBox[{"glucose", "[", "t", "]"}], "lambda"], " ",

RowBox[{"rbc7", "[", "t", "]"}]}]}]}]},

{

RowBox[{

RowBox[{

SuperscriptBox["rbc8", "\[Prime]",

MultilineFunction->None], "[", "t", "]"}], "\[Equal]",

RowBox[{

RowBox[{"Ktr", " ",

RowBox[{"rbc7", "[", "t", "]"}]}], "-",

RowBox[{"Ktr", " ",

RowBox[{"rbc8", "[", "t", "]"}]}], "-",

RowBox[{"Kglucose", " ",

SuperscriptBox[

RowBox[{"glucose", "[", "t", "]"}], "lambda"], " ",

RowBox[{"rbc8", "[", "t", "]"}]}]}]}]},

{

RowBox[{

RowBox[{

SuperscriptBox["rbc9", "\[Prime]",

MultilineFunction->None], "[", "t", "]"}], "\[Equal]",

RowBox[{

RowBox[{"Ktr", " ",

RowBox[{"rbc8", "[", "t", "]"}]}], "-",

RowBox[{"Ktr", " ",

RowBox[{"rbc9", "[", "t", "]"}]}], "-",

RowBox[{"Kglucose", " ",

SuperscriptBox[

RowBox[{"glucose", "[", "t", "]"}], "lambda"], " ",

RowBox[{"rbc9", "[", "t", "]"}]}]}]}]},

{

RowBox[{

RowBox[{

SuperscriptBox["rbc10", "\[Prime]",

MultilineFunction->None], "[", "t", "]"}], "\[Equal]",

RowBox[{

RowBox[{

RowBox[{"-", "Ktr"}], " ",

RowBox[{"rbc10", "[", "t", "]"}]}], "-",

RowBox[{"Kglucose", " ",

SuperscriptBox[

RowBox[{"glucose", "[", "t", "]"}], "lambda"], " ",

RowBox[{"rbc10", "[", "t", "]"}]}], "+",

RowBox[{"Ktr", " ",

RowBox[{"rbc9", "[", "t", "]"}]}]}]}]},

{

RowBox[{

RowBox[{

SuperscriptBox["rbc11", "\[Prime]",

MultilineFunction->None], "[", "t", "]"}], "\[Equal]",

RowBox[{

RowBox[{"Ktr", " ",

RowBox[{"rbc10", "[", "t", "]"}]}], "-",

RowBox[{"Ktr", " ",

RowBox[{"rbc11", "[", "t", "]"}]}], "-",

RowBox[{"Kglucose", " ",

SuperscriptBox[

RowBox[{"glucose", "[", "t", "]"}], "lambda"], " ",

RowBox[{"rbc11", "[", "t", "]"}]}]}]}]},

{

RowBox[{

RowBox[{

SuperscriptBox["rbc12", "\[Prime]",

MultilineFunction->None], "[", "t", "]"}], "\[Equal]",

RowBox[{

RowBox[{"Ktr", " ",

RowBox[{"rbc11", "[", "t", "]"}]}], "-",

RowBox[{"Ktr", " ",

RowBox[{"rbc12", "[", "t", "]"}]}], "-",

RowBox[{"Kglucose", " ",

SuperscriptBox[

RowBox[{"glucose", "[", "t", "]"}], "lambda"], " ",

RowBox[{"rbc12", "[", "t", "]"}]}]}]}]},

{

RowBox[{

RowBox[{

SuperscriptBox["a1c1", "\[Prime]",

MultilineFunction->None], "[", "t", "]"}], "\[Equal]",

RowBox[{

RowBox[{

RowBox[{"-", "Ktr"}], " ",

RowBox[{"a1c1", "[", "t", "]"}]}], "+",

RowBox[{"Kglucose", " ",

SuperscriptBox[

RowBox[{"glucose", "[", "t", "]"}], "lambda"], " ",

RowBox[{"rbc1", "[", "t", "]"}]}]}]}]},

{

RowBox[{

RowBox[{

SuperscriptBox["a1c2", "\[Prime]",

MultilineFunction->None], "[", "t", "]"}], "\[Equal]",

RowBox[{

RowBox[{"Ktr", " ",

RowBox[{"a1c1", "[", "t", "]"}]}], "-",

RowBox[{"Ktr", " ",

RowBox[{"a1c2", "[", "t", "]"}]}], "+",

RowBox[{"Kglucose", " ",

SuperscriptBox[

RowBox[{"glucose", "[", "t", "]"}], "lambda"], " ",

RowBox[{"rbc2", "[", "t", "]"}]}]}]}]},

{

RowBox[{

RowBox[{

SuperscriptBox["a1c3", "\[Prime]",

MultilineFunction->None], "[", "t", "]"}], "\[Equal]",

RowBox[{

RowBox[{"Ktr", " ",

RowBox[{"a1c2", "[", "t", "]"}]}], "-",

RowBox[{"Ktr", " ",

RowBox[{"a1c3", "[", "t", "]"}]}], "+",

RowBox[{"Kglucose", " ",

SuperscriptBox[

RowBox[{"glucose", "[", "t", "]"}], "lambda"], " ",

RowBox[{"rbc3", "[", "t", "]"}]}]}]}]},

{

RowBox[{

RowBox[{

SuperscriptBox["a1c4", "\[Prime]",

MultilineFunction->None], "[", "t", "]"}], "\[Equal]",

RowBox[{

RowBox[{"Ktr", " ",

RowBox[{"a1c3", "[", "t", "]"}]}], "-",

RowBox[{"Ktr", " ",

RowBox[{"a1c4", "[", "t", "]"}]}], "+",

RowBox[{"Kglucose", " ",

SuperscriptBox[

RowBox[{"glucose", "[", "t", "]"}], "lambda"], " ",

RowBox[{"rbc4", "[", "t", "]"}]}]}]}]},

{

RowBox[{

RowBox[{

SuperscriptBox["a1c5", "\[Prime]",

MultilineFunction->None], "[", "t", "]"}], "\[Equal]",

RowBox[{

RowBox[{"Ktr", " ",

RowBox[{"a1c4", "[", "t", "]"}]}], "-",

RowBox[{"Ktr", " ",

RowBox[{"a1c5", "[", "t", "]"}]}], "+",

RowBox[{"Kglucose", " ",

SuperscriptBox[

RowBox[{"glucose", "[", "t", "]"}], "lambda"], " ",

RowBox[{"rbc5", "[", "t", "]"}]}]}]}]},

{

RowBox[{

RowBox[{

SuperscriptBox["a1c6", "\[Prime]",

MultilineFunction->None], "[", "t", "]"}], "\[Equal]",

RowBox[{

RowBox[{"Ktr", " ",

RowBox[{"a1c5", "[", "t", "]"}]}], "-",

RowBox[{"Ktr", " ",

RowBox[{"a1c6", "[", "t", "]"}]}], "+",

RowBox[{"Kglucose", " ",

SuperscriptBox[

RowBox[{"glucose", "[", "t", "]"}], "lambda"], " ",

RowBox[{"rbc6", "[", "t", "]"}]}]}]}]},

{

RowBox[{

RowBox[{

SuperscriptBox["a1c7", "\[Prime]",

MultilineFunction->None], "[", "t", "]"}], "\[Equal]",

RowBox[{

RowBox[{"Ktr", " ",

RowBox[{"a1c6", "[", "t", "]"}]}], "-",

RowBox[{"Ktr", " ",

RowBox[{"a1c7", "[", "t", "]"}]}], "+",

RowBox[{"Kglucose", " ",

SuperscriptBox[

RowBox[{"glucose", "[", "t", "]"}], "lambda"], " ",

RowBox[{"rbc7", "[", "t", "]"}]}]}]}]},

{

RowBox[{

RowBox[{

SuperscriptBox["a1c8", "\[Prime]",

MultilineFunction->None], "[", "t", "]"}], "\[Equal]",

RowBox[{

RowBox[{"Ktr", " ",

RowBox[{"a1c7", "[", "t", "]"}]}], "-",

RowBox[{"Ktr", " ",

RowBox[{"a1c8", "[", "t", "]"}]}], "+",

RowBox[{"Kglucose", " ",

SuperscriptBox[

RowBox[{"glucose", "[", "t", "]"}], "lambda"], " ",

RowBox[{"rbc8", "[", "t", "]"}]}]}]}]},

{

RowBox[{

RowBox[{

SuperscriptBox["a1c9", "\[Prime]",

MultilineFunction->None], "[", "t", "]"}], "\[Equal]",

RowBox[{

RowBox[{"Ktr", " ",

RowBox[{"a1c8", "[", "t", "]"}]}], "-",

RowBox[{"Ktr", " ",

RowBox[{"a1c9", "[", "t", "]"}]}], "+",

RowBox[{"Kglucose", " ",

SuperscriptBox[

RowBox[{"glucose", "[", "t", "]"}], "lambda"], " ",

RowBox[{"rbc9", "[", "t", "]"}]}]}]}]},

{

RowBox[{

RowBox[{

SuperscriptBox["a1c10", "\[Prime]",

MultilineFunction->None], "[", "t", "]"}], "\[Equal]",

RowBox[{

RowBox[{

RowBox[{"-", "Ktr"}], " ",

RowBox[{"a1c10", "[", "t", "]"}]}], "+",

RowBox[{"Ktr", " ",

RowBox[{"a1c9", "[", "t", "]"}]}], "+",

RowBox[{"Kglucose", " ",

SuperscriptBox[

RowBox[{"glucose", "[", "t", "]"}], "lambda"], " ",

RowBox[{"rbc10", "[", "t", "]"}]}]}]}]},

{

RowBox[{

RowBox[{

SuperscriptBox["a1c11", "\[Prime]",

MultilineFunction->None], "[", "t", "]"}], "\[Equal]",

RowBox[{

RowBox[{"Ktr", " ",

RowBox[{"a1c10", "[", "t", "]"}]}], "-",

RowBox[{"Ktr", " ",

RowBox[{"a1c11", "[", "t", "]"}]}], "+",

RowBox[{"Kglucose", " ",

SuperscriptBox[

RowBox[{"glucose", "[", "t", "]"}], "lambda"], " ",

RowBox[{"rbc11", "[", "t", "]"}]}]}]}]},

{

RowBox[{

RowBox[{

SuperscriptBox["a1c12", "\[Prime]",

MultilineFunction->None], "[", "t", "]"}], "\[Equal]",

RowBox[{

RowBox[{"Ktr", " ",

RowBox[{"a1c11", "[", "t", "]"}]}], "-",

RowBox[{"Ktr", " ",

RowBox[{"a1c12", "[", "t", "]"}]}], "+",

RowBox[{"Kglucose", " ",

SuperscriptBox[

RowBox[{"glucose", "[", "t", "]"}], "lambda"], " ",

RowBox[{"rbc12", "[", "t", "]"}]}]}]}]},

{

RowBox[{

RowBox[{

SuperscriptBox["isc", "\[Prime]",

MultilineFunction->None], "[", "t", "]"}], "\[Equal]",

RowBox[{"taus", " ",

RowBox[{"(",

RowBox[{

RowBox[{"ks", " ",

RowBox[{"(",

RowBox[{"1", "-",

FractionBox[

RowBox[{"vs", " ",

RowBox[{"il1r", "[", "t", "]"}]}],

RowBox[{"kms", "+",

RowBox[{"il1r", "[", "t", "]"}]}]]}], ")"}]}], "-",

RowBox[{"isc", "[", "t", "]"}]}], ")"}]}]}]},

{

RowBox[{

RowBox[{

SuperscriptBox["f", "\[Prime]",

MultilineFunction->None], "[", "t", "]"}], "\[Equal]",

RowBox[{"tauf", " ",

RowBox[{"(",

RowBox[{

RowBox[{"-",

RowBox[{"f", "[", "t", "]"}]}], "+",

RowBox[{"kf", " ",

RowBox[{"(",

RowBox[{"1", "+",

FractionBox[

RowBox[{"vfg", " ",

SuperscriptBox[

RowBox[{"glucose", "[", "t", "]"}], "xfg"]}],

RowBox[{

SuperscriptBox["kmfg", "xfg"], "+",

SuperscriptBox[

RowBox[{"glucose", "[", "t", "]"}], "xfg"]}]]}], ")"}], " ",

RowBox[{"(",

RowBox[{"1", "+",

FractionBox[

RowBox[{"vf", " ",

RowBox[{"il1r", "[", "t", "]"}]}],

RowBox[{"kmf", "+",

RowBox[{"il1r", "[", "t", "]"}]}]]}], ")"}]}]}], ")"}]}]}]},

{

RowBox[{

RowBox[{

SuperscriptBox["b", "\[Prime]",

MultilineFunction->None], "[", "t", "]"}], "\[Equal]",

RowBox[{

RowBox[{"b", "[", "t", "]"}], " ",

RowBox[{"(",

RowBox[{

RowBox[{"-",

RowBox[{"apoptosis", "[", "t", "]"}]}], "+",

RowBox[{"replication", "[", "t", "]"}]}], ")"}]}]}]},

{

RowBox[{

RowBox[{"apoptosis", "[", "t", "]"}], "\[Equal]",

RowBox[{"ka", " ",

RowBox[{"(",

RowBox[{"1", "+",

FractionBox[

RowBox[{"vha", " ",

SuperscriptBox[

RowBox[{"il1r", "[", "t", "]"}], "xha"]}],

RowBox[{

SuperscriptBox["kmha", "xha"], "+",

SuperscriptBox[

RowBox[{"il1r", "[", "t", "]"}], "xha"]}]], "-",

FractionBox[

RowBox[{"vla", " ",

SuperscriptBox[

RowBox[{"il1r", "[", "t", "]"}], "xla"]}],

RowBox[{

SuperscriptBox["kmla", "xla"], "+",

SuperscriptBox[

RowBox[{"il1r", "[", "t", "]"}], "xla"]}]]}], ")"}]}]}]},

{

RowBox[{

RowBox[{"replication", "[", "t", "]"}], "\[Equal]",

RowBox[{"kr", " ",

RowBox[{"(",

RowBox[{"1", "-",

FractionBox[

RowBox[{"vhr", " ",

SuperscriptBox[

RowBox[{"il1r", "[", "t", "]"}], "xhr"]}],

RowBox[{

SuperscriptBox["kmhr", "xhr"], "+",

SuperscriptBox[

RowBox[{"il1r", "[", "t", "]"}], "xhr"]}]], "+",

FractionBox[

RowBox[{"vlr", " ",

SuperscriptBox[

RowBox[{"il1r", "[", "t", "]"}], "xlr"]}],

RowBox[{

SuperscriptBox["kmlr", "xlr"], "+",

SuperscriptBox[

RowBox[{"il1r", "[", "t", "]"}], "xlr"]}]]}], ")"}]}]}]},

{

RowBox[{

RowBox[{"il1r", "[", "t", "]"}], "\[Equal]",

FractionBox[

RowBox[{"il1b", "[", "t", "]"}],

RowBox[{

RowBox[{"il1b", "[", "t", "]"}], "+",

RowBox[{"km", " ",

RowBox[{"(",

RowBox[{"1", "+",

FractionBox[

RowBox[{

RowBox[{"il1ra", "[", "t", "]"}], "+",

RowBox[{"1000", " ",

RowBox[{

TagBox[

RowBox[{"InterpolatingFunction", "[",

RowBox[{

RowBox[{"{",

RowBox[{"{",

RowBox[{"0.`", ",", "10000.`"}], "}"}], "}"}], ",",

StyleBox["\<\"<>\"\>",

ShowStringCharacters->False]}], "]"}],

False,

Editable->False,

SelectWithContents->True], "[", "t", "]"}]}]}], "ki"]}],

")"}]}]}]]}]},

{

RowBox[{

RowBox[{

SuperscriptBox["il1b", "\[Prime]",

MultilineFunction->None], "[", "t", "]"}], "\[Equal]",

RowBox[{"If", "[",

RowBox[{

RowBox[{"0", "<", "t", "\[LessEqual]", "91"}], ",",

RowBox[{

RowBox[{"(",

RowBox[{"il1bH", "-",

RowBox[{"il1b", "[", "t", "]"}]}], ")"}], " ", "k1"}], ",",

RowBox[{"k2", " ",

RowBox[{"(",

RowBox[{

RowBox[{"(",

RowBox[{"il1b0", "+",

RowBox[{"kplacebo", " ", "t"}]}], ")"}], "-",

RowBox[{"il1b", "[", "t", "]"}]}], ")"}]}]}], "]"}]}]},

{

RowBox[{

RowBox[{

SuperscriptBox["il1ra", "\[Prime]",

MultilineFunction->None], "[", "t", "]"}], "\[Equal]", "0"}]}

},

GridBoxAlignment->{

"Columns" -> {{Left}}, "ColumnsIndexed" -> {}, "Rows" -> {{Baseline}},

"RowsIndexed" -> {}},

GridBoxSpacings->{"Columns" -> {

Offset[0.27999999999999997`], {

Offset[0.5599999999999999]},

Offset[0.27999999999999997`]}, "ColumnsIndexed" -> {}, "Rows" -> {

Offset[0.2], {

Offset[0.4]},

Offset[0.2]}, "RowsIndexed" -> {}}],

Column],

Function[BoxForm`e$,

TableForm[BoxForm`e$]]]], "Output",

CellChangeTimes->{3.602939583768203*^9}]

}, Open ]]

}, Closed]],

Cell[CellGroupData[{

Cell["Placebo model", "Subsection",

CellChangeTimes->{{3.602939621882617*^9, 3.6029396257855816`*^9}}],

Cell[CellGroupData[{

Cell[BoxData[

RowBox[{"eqsSysPlacebo", "//", "TableForm"}]], "Input",

CellChangeTimes->{{3.6029396271709237`*^9, 3.6029396342846804`*^9}}],

Cell[BoxData[

TagBox[

TagBox[GridBox[{

{

RowBox[{

RowBox[{"glucose", "[", "t", "]"}], "\[Equal]",

FractionBox["Tgl",

RowBox[{"Kxg", "+",

RowBox[{"Kxgi", " ",

RowBox[{"insulin", "[", "t", "]"}]}], "+",

RowBox[{"0.1`", " ", "Kxgi", " ",

RowBox[{"proinsulin", "[", "t", "]"}]}]}]]}]},

{

RowBox[{

RowBox[{"insulin", "[", "t", "]"}], "\[Equal]",

FractionBox[

RowBox[{

RowBox[{"b", "[", "t", "]"}], " ",

SuperscriptBox[

RowBox[{"(",

FractionBox[

RowBox[{"glucose", "[", "t", "]"}], "Gh"], ")"}], "vh"], " ",

RowBox[{"isc", "[", "t", "]"}]}],

RowBox[{"Kxi", " ",

RowBox[{"(",

RowBox[{"1", "+",

SuperscriptBox[

RowBox[{"(",

FractionBox[

RowBox[{"glucose", "[", "t", "]"}], "Gh"], ")"}], "vh"]}],

")"}]}]]}]},

{

RowBox[{

RowBox[{"proinsulin", "[", "t", "]"}], "\[Equal]",

FractionBox[

RowBox[{"10.`", " ",

RowBox[{"b", "[", "t", "]"}], " ",

RowBox[{"f", "[", "t", "]"}], " ",

SuperscriptBox[

RowBox[{"(",

FractionBox[

RowBox[{"glucose", "[", "t", "]"}], "Gh"], ")"}], "vh"], " ",

RowBox[{"isc", "[", "t", "]"}]}],

RowBox[{"Kxi", " ",

RowBox[{"(",

RowBox[{"1", "+",

SuperscriptBox[

RowBox[{"(",

FractionBox[

RowBox[{"glucose", "[", "t", "]"}], "Gh"], ")"}], "vh"]}],

")"}]}]]}]},

{

RowBox[{

RowBox[{"hba1c", "[", "t", "]"}], "\[Equal]",

FractionBox[

RowBox[{"100", " ",

RowBox[{"(",

RowBox[{

RowBox[{"a1c1", "[", "t", "]"}], "+",

RowBox[{"a1c10", "[", "t", "]"}], "+",

RowBox[{"a1c11", "[", "t", "]"}], "+",

RowBox[{"a1c12", "[", "t", "]"}], "+",

RowBox[{"a1c2", "[", "t", "]"}], "+",

RowBox[{"a1c3", "[", "t", "]"}], "+",

RowBox[{"a1c4", "[", "t", "]"}], "+",

RowBox[{"a1c5", "[", "t", "]"}], "+",

RowBox[{"a1c6", "[", "t", "]"}], "+",

RowBox[{"a1c7", "[", "t", "]"}], "+",

RowBox[{"a1c8", "[", "t", "]"}], "+",

RowBox[{"a1c9", "[", "t", "]"}]}], ")"}]}],

RowBox[{

RowBox[{"a1c1", "[", "t", "]"}], "+",

RowBox[{"a1c10", "[", "t", "]"}], "+",

RowBox[{"a1c11", "[", "t", "]"}], "+",

RowBox[{"a1c12", "[", "t", "]"}], "+",

RowBox[{"a1c2", "[", "t", "]"}], "+",

RowBox[{"a1c3", "[", "t", "]"}], "+",

RowBox[{"a1c4", "[", "t", "]"}], "+",

RowBox[{"a1c5", "[", "t", "]"}], "+",

RowBox[{"a1c6", "[", "t", "]"}], "+",

RowBox[{"a1c7", "[", "t", "]"}], "+",

RowBox[{"a1c8", "[", "t", "]"}], "+",

RowBox[{"a1c9", "[", "t", "]"}], "+",

RowBox[{"rbc1", "[", "t", "]"}], "+",

RowBox[{"rbc10", "[", "t", "]"}], "+",

RowBox[{"rbc11", "[", "t", "]"}], "+",

RowBox[{"rbc12", "[", "t", "]"}], "+",

RowBox[{"rbc2", "[", "t", "]"}], "+",

RowBox[{"rbc3", "[", "t", "]"}], "+",

RowBox[{"rbc4", "[", "t", "]"}], "+",

RowBox[{"rbc5", "[", "t", "]"}], "+",

RowBox[{"rbc6", "[", "t", "]"}], "+",

RowBox[{"rbc7", "[", "t", "]"}], "+",

RowBox[{"rbc8", "[", "t", "]"}], "+",

RowBox[{"rbc9", "[", "t", "]"}]}]]}]},

{

RowBox[{

RowBox[{

SuperscriptBox["rbc1", "\[Prime]",

MultilineFunction->None], "[", "t", "]"}], "\[Equal]",

RowBox[{"Kin", "-",

RowBox[{"Ktr", " ",

RowBox[{"rbc1", "[", "t", "]"}]}], "-",

RowBox[{"Kglucose", " ",

SuperscriptBox[

RowBox[{"glucose", "[", "t", "]"}], "lambda"], " ",

RowBox[{"rbc1", "[", "t", "]"}]}]}]}]},

{

RowBox[{

RowBox[{

SuperscriptBox["rbc2", "\[Prime]",

MultilineFunction->None], "[", "t", "]"}], "\[Equal]",

RowBox[{

RowBox[{"Ktr", " ",

RowBox[{"rbc1", "[", "t", "]"}]}], "-",

RowBox[{"Ktr", " ",

RowBox[{"rbc2", "[", "t", "]"}]}], "-",

RowBox[{"Kglucose", " ",

SuperscriptBox[

RowBox[{"glucose", "[", "t", "]"}], "lambda"], " ",

RowBox[{"rbc2", "[", "t", "]"}]}]}]}]},

{

RowBox[{

RowBox[{

SuperscriptBox["rbc3", "\[Prime]",

MultilineFunction->None], "[", "t", "]"}], "\[Equal]",

RowBox[{

RowBox[{"Ktr", " ",

RowBox[{"rbc2", "[", "t", "]"}]}], "-",

RowBox[{"Ktr", " ",

RowBox[{"rbc3", "[", "t", "]"}]}], "-",

RowBox[{"Kglucose", " ",

SuperscriptBox[

RowBox[{"glucose", "[", "t", "]"}], "lambda"], " ",

RowBox[{"rbc3", "[", "t", "]"}]}]}]}]},

{

RowBox[{

RowBox[{

SuperscriptBox["rbc4", "\[Prime]",

MultilineFunction->None], "[", "t", "]"}], "\[Equal]",

RowBox[{

RowBox[{"Ktr", " ",

RowBox[{"rbc3", "[", "t", "]"}]}], "-",

RowBox[{"Ktr", " ",

RowBox[{"rbc4", "[", "t", "]"}]}], "-",

RowBox[{"Kglucose", " ",

SuperscriptBox[

RowBox[{"glucose", "[", "t", "]"}], "lambda"], " ",

RowBox[{"rbc4", "[", "t", "]"}]}]}]}]},

{

RowBox[{

RowBox[{

SuperscriptBox["rbc5", "\[Prime]",

MultilineFunction->None], "[", "t", "]"}], "\[Equal]",

RowBox[{

RowBox[{"Ktr", " ",

RowBox[{"rbc4", "[", "t", "]"}]}], "-",

RowBox[{"Ktr", " ",

RowBox[{"rbc5", "[", "t", "]"}]}], "-",

RowBox[{"Kglucose", " ",

SuperscriptBox[

RowBox[{"glucose", "[", "t", "]"}], "lambda"], " ",

RowBox[{"rbc5", "[", "t", "]"}]}]}]}]},

{

RowBox[{

RowBox[{

SuperscriptBox["rbc6", "\[Prime]",

MultilineFunction->None], "[", "t", "]"}], "\[Equal]",

RowBox[{

RowBox[{"Ktr", " ",

RowBox[{"rbc5", "[", "t", "]"}]}], "-",

RowBox[{"Ktr", " ",

RowBox[{"rbc6", "[", "t", "]"}]}], "-",

RowBox[{"Kglucose", " ",

SuperscriptBox[

RowBox[{"glucose", "[", "t", "]"}], "lambda"], " ",

RowBox[{"rbc6", "[", "t", "]"}]}]}]}]},

{

RowBox[{

RowBox[{

SuperscriptBox["rbc7", "\[Prime]",

MultilineFunction->None], "[", "t", "]"}], "\[Equal]",

RowBox[{

RowBox[{"Ktr", " ",

RowBox[{"rbc6", "[", "t", "]"}]}], "-",

RowBox[{"Ktr", " ",

RowBox[{"rbc7", "[", "t", "]"}]}], "-",

RowBox[{"Kglucose", " ",

SuperscriptBox[

RowBox[{"glucose", "[", "t", "]"}], "lambda"], " ",

RowBox[{"rbc7", "[", "t", "]"}]}]}]}]},

{

RowBox[{

RowBox[{

SuperscriptBox["rbc8", "\[Prime]",

MultilineFunction->None], "[", "t", "]"}], "\[Equal]",

RowBox[{

RowBox[{"Ktr", " ",

RowBox[{"rbc7", "[", "t", "]"}]}], "-",

RowBox[{"Ktr", " ",

RowBox[{"rbc8", "[", "t", "]"}]}], "-",

RowBox[{"Kglucose", " ",

SuperscriptBox[

RowBox[{"glucose", "[", "t", "]"}], "lambda"], " ",

RowBox[{"rbc8", "[", "t", "]"}]}]}]}]},

{

RowBox[{

RowBox[{

SuperscriptBox["rbc9", "\[Prime]",

MultilineFunction->None], "[", "t", "]"}], "\[Equal]",

RowBox[{

RowBox[{"Ktr", " ",

RowBox[{"rbc8", "[", "t", "]"}]}], "-",

RowBox[{"Ktr", " ",

RowBox[{"rbc9", "[", "t", "]"}]}], "-",

RowBox[{"Kglucose", " ",

SuperscriptBox[

RowBox[{"glucose", "[", "t", "]"}], "lambda"], " ",

RowBox[{"rbc9", "[", "t", "]"}]}]}]}]},

{

RowBox[{

RowBox[{

SuperscriptBox["rbc10", "\[Prime]",

MultilineFunction->None], "[", "t", "]"}], "\[Equal]",

RowBox[{

RowBox[{

RowBox[{"-", "Ktr"}], " ",

RowBox[{"rbc10", "[", "t", "]"}]}], "-",

RowBox[{"Kglucose", " ",

SuperscriptBox[

RowBox[{"glucose", "[", "t", "]"}], "lambda"], " ",

RowBox[{"rbc10", "[", "t", "]"}]}], "+",

RowBox[{"Ktr", " ",

RowBox[{"rbc9", "[", "t", "]"}]}]}]}]},

{

RowBox[{

RowBox[{

SuperscriptBox["rbc11", "\[Prime]",

MultilineFunction->None], "[", "t", "]"}], "\[Equal]",

RowBox[{

RowBox[{"Ktr", " ",

RowBox[{"rbc10", "[", "t", "]"}]}], "-",

RowBox[{"Ktr", " ",

RowBox[{"rbc11", "[", "t", "]"}]}], "-",

RowBox[{"Kglucose", " ",

SuperscriptBox[

RowBox[{"glucose", "[", "t", "]"}], "lambda"], " ",

RowBox[{"rbc11", "[", "t", "]"}]}]}]}]},

{

RowBox[{

RowBox[{

SuperscriptBox["rbc12", "\[Prime]",

MultilineFunction->None], "[", "t", "]"}], "\[Equal]",

RowBox[{

RowBox[{"Ktr", " ",

RowBox[{"rbc11", "[", "t", "]"}]}], "-",

RowBox[{"Ktr", " ",

RowBox[{"rbc12", "[", "t", "]"}]}], "-",

RowBox[{"Kglucose", " ",

SuperscriptBox[

RowBox[{"glucose", "[", "t", "]"}], "lambda"], " ",

RowBox[{"rbc12", "[", "t", "]"}]}]}]}]},

{

RowBox[{

RowBox[{

SuperscriptBox["a1c1", "\[Prime]",

MultilineFunction->None], "[", "t", "]"}], "\[Equal]",

RowBox[{

RowBox[{

RowBox[{"-", "Ktr"}], " ",

RowBox[{"a1c1", "[", "t", "]"}]}], "+",

RowBox[{"Kglucose", " ",

SuperscriptBox[

RowBox[{"glucose", "[", "t", "]"}], "lambda"], " ",

RowBox[{"rbc1", "[", "t", "]"}]}]}]}]},

{

RowBox[{

RowBox[{

SuperscriptBox["a1c2", "\[Prime]",

MultilineFunction->None], "[", "t", "]"}], "\[Equal]",

RowBox[{

RowBox[{"Ktr", " ",

RowBox[{"a1c1", "[", "t", "]"}]}], "-",

RowBox[{"Ktr", " ",

RowBox[{"a1c2", "[", "t", "]"}]}], "+",

RowBox[{"Kglucose", " ",

SuperscriptBox[

RowBox[{"glucose", "[", "t", "]"}], "lambda"], " ",

RowBox[{"rbc2", "[", "t", "]"}]}]}]}]},

{

RowBox[{

RowBox[{

SuperscriptBox["a1c3", "\[Prime]",

MultilineFunction->None], "[", "t", "]"}], "\[Equal]",

RowBox[{

RowBox[{"Ktr", " ",

RowBox[{"a1c2", "[", "t", "]"}]}], "-",

RowBox[{"Ktr", " ",

RowBox[{"a1c3", "[", "t", "]"}]}], "+",

RowBox[{"Kglucose", " ",

SuperscriptBox[

RowBox[{"glucose", "[", "t", "]"}], "lambda"], " ",

RowBox[{"rbc3", "[", "t", "]"}]}]}]}]},

{

RowBox[{

RowBox[{

SuperscriptBox["a1c4", "\[Prime]",

MultilineFunction->None], "[", "t", "]"}], "\[Equal]",

RowBox[{

RowBox[{"Ktr", " ",

RowBox[{"a1c3", "[", "t", "]"}]}], "-",

RowBox[{"Ktr", " ",

RowBox[{"a1c4", "[", "t", "]"}]}], "+",

RowBox[{"Kglucose", " ",

SuperscriptBox[

RowBox[{"glucose", "[", "t", "]"}], "lambda"], " ",

RowBox[{"rbc4", "[", "t", "]"}]}]}]}]},

{

RowBox[{

RowBox[{

SuperscriptBox["a1c5", "\[Prime]",

MultilineFunction->None], "[", "t", "]"}], "\[Equal]",

RowBox[{

RowBox[{"Ktr", " ",

RowBox[{"a1c4", "[", "t", "]"}]}], "-",

RowBox[{"Ktr", " ",

RowBox[{"a1c5", "[", "t", "]"}]}], "+",

RowBox[{"Kglucose", " ",

SuperscriptBox[

RowBox[{"glucose", "[", "t", "]"}], "lambda"], " ",

RowBox[{"rbc5", "[", "t", "]"}]}]}]}]},

{

RowBox[{

RowBox[{

SuperscriptBox["a1c6", "\[Prime]",

MultilineFunction->None], "[", "t", "]"}], "\[Equal]",

RowBox[{

RowBox[{"Ktr", " ",

RowBox[{"a1c5", "[", "t", "]"}]}], "-",

RowBox[{"Ktr", " ",

RowBox[{"a1c6", "[", "t", "]"}]}], "+",

RowBox[{"Kglucose", " ",

SuperscriptBox[

RowBox[{"glucose", "[", "t", "]"}], "lambda"], " ",

RowBox[{"rbc6", "[", "t", "]"}]}]}]}]},

{

RowBox[{

RowBox[{

SuperscriptBox["a1c7", "\[Prime]",

MultilineFunction->None], "[", "t", "]"}], "\[Equal]",

RowBox[{

RowBox[{"Ktr", " ",

RowBox[{"a1c6", "[", "t", "]"}]}], "-",

RowBox[{"Ktr", " ",

RowBox[{"a1c7", "[", "t", "]"}]}], "+",

RowBox[{"Kglucose", " ",

SuperscriptBox[

RowBox[{"glucose", "[", "t", "]"}], "lambda"], " ",

RowBox[{"rbc7", "[", "t", "]"}]}]}]}]},

{

RowBox[{

RowBox[{

SuperscriptBox["a1c8", "\[Prime]",

MultilineFunction->None], "[", "t", "]"}], "\[Equal]",

RowBox[{

RowBox[{"Ktr", " ",

RowBox[{"a1c7", "[", "t", "]"}]}], "-",

RowBox[{"Ktr", " ",

RowBox[{"a1c8", "[", "t", "]"}]}], "+",

RowBox[{"Kglucose", " ",

SuperscriptBox[

RowBox[{"glucose", "[", "t", "]"}], "lambda"], " ",

RowBox[{"rbc8", "[", "t", "]"}]}]}]}]},

{

RowBox[{

RowBox[{

SuperscriptBox["a1c9", "\[Prime]",

MultilineFunction->None], "[", "t", "]"}], "\[Equal]",

RowBox[{

RowBox[{"Ktr", " ",

RowBox[{"a1c8", "[", "t", "]"}]}], "-",

RowBox[{"Ktr", " ",

RowBox[{"a1c9", "[", "t", "]"}]}], "+",

RowBox[{"Kglucose", " ",

SuperscriptBox[

RowBox[{"glucose", "[", "t", "]"}], "lambda"], " ",

RowBox[{"rbc9", "[", "t", "]"}]}]}]}]},

{

RowBox[{

RowBox[{

SuperscriptBox["a1c10", "\[Prime]",

MultilineFunction->None], "[", "t", "]"}], "\[Equal]",

RowBox[{

RowBox[{

RowBox[{"-", "Ktr"}], " ",

RowBox[{"a1c10", "[", "t", "]"}]}], "+",

RowBox[{"Ktr", " ",

RowBox[{"a1c9", "[", "t", "]"}]}], "+",

RowBox[{"Kglucose", " ",

SuperscriptBox[

RowBox[{"glucose", "[", "t", "]"}], "lambda"], " ",

RowBox[{"rbc10", "[", "t", "]"}]}]}]}]},

{

RowBox[{

RowBox[{

SuperscriptBox["a1c11", "\[Prime]",

MultilineFunction->None], "[", "t", "]"}], "\[Equal]",

RowBox[{

RowBox[{"Ktr", " ",

RowBox[{"a1c10", "[", "t", "]"}]}], "-",

RowBox[{"Ktr", " ",

RowBox[{"a1c11", "[", "t", "]"}]}], "+",

RowBox[{"Kglucose", " ",

SuperscriptBox[

RowBox[{"glucose", "[", "t", "]"}], "lambda"], " ",

RowBox[{"rbc11", "[", "t", "]"}]}]}]}]},

{

RowBox[{

RowBox[{

SuperscriptBox["a1c12", "\[Prime]",

MultilineFunction->None], "[", "t", "]"}], "\[Equal]",

RowBox[{

RowBox[{"Ktr", " ",

RowBox[{"a1c11", "[", "t", "]"}]}], "-",

RowBox[{"Ktr", " ",

RowBox[{"a1c12", "[", "t", "]"}]}], "+",

RowBox[{"Kglucose", " ",

SuperscriptBox[

RowBox[{"glucose", "[", "t", "]"}], "lambda"], " ",

RowBox[{"rbc12", "[", "t", "]"}]}]}]}]},

{

RowBox[{

RowBox[{

SuperscriptBox["isc", "\[Prime]",

MultilineFunction->None], "[", "t", "]"}], "\[Equal]",

RowBox[{"taus", " ",

RowBox[{"(",

RowBox[{

RowBox[{"ks", " ",

RowBox[{"(",

RowBox[{"1", "-",

FractionBox[

RowBox[{"vs", " ",

RowBox[{"il1r", "[", "t", "]"}]}],

RowBox[{"kms", "+",

RowBox[{"il1r", "[", "t", "]"}]}]]}], ")"}]}], "-",

RowBox[{"isc", "[", "t", "]"}]}], ")"}]}]}]},

{

RowBox[{

RowBox[{

SuperscriptBox["f", "\[Prime]",

MultilineFunction->None], "[", "t", "]"}], "\[Equal]",

RowBox[{"tauf", " ",

RowBox[{"(",

RowBox[{

RowBox[{"-",

RowBox[{"f", "[", "t", "]"}]}], "+",

RowBox[{"kf", " ",

RowBox[{"(",

RowBox[{"1", "+",

FractionBox[

RowBox[{"vfg", " ",

SuperscriptBox[

RowBox[{"glucose", "[", "t", "]"}], "xfg"]}],

RowBox[{

SuperscriptBox["kmfg", "xfg"], "+",

SuperscriptBox[

RowBox[{"glucose", "[", "t", "]"}], "xfg"]}]]}], ")"}], " ",

RowBox[{"(",

RowBox[{"1", "+",

FractionBox[

RowBox[{"vf", " ",

RowBox[{"il1r", "[", "t", "]"}]}],

RowBox[{"kmf", "+",

RowBox[{"il1r", "[", "t", "]"}]}]]}], ")"}]}]}], ")"}]}]}]},

{

RowBox[{

RowBox[{

SuperscriptBox["b", "\[Prime]",

MultilineFunction->None], "[", "t", "]"}], "\[Equal]",

RowBox[{

RowBox[{"b", "[", "t", "]"}], " ",

RowBox[{"(",

RowBox[{

RowBox[{"-",

RowBox[{"apoptosis", "[", "t", "]"}]}], "+",

RowBox[{"replication", "[", "t", "]"}]}], ")"}]}]}]},

{

RowBox[{

RowBox[{"apoptosis", "[", "t", "]"}], "\[Equal]",

RowBox[{"ka", " ",

RowBox[{"(",

RowBox[{"1", "+",

FractionBox[

RowBox[{"vha", " ",

SuperscriptBox[

RowBox[{"il1r", "[", "t", "]"}], "xha"]}],

RowBox[{

SuperscriptBox["kmha", "xha"], "+",

SuperscriptBox[

RowBox[{"il1r", "[", "t", "]"}], "xha"]}]], "-",

FractionBox[

RowBox[{"vla", " ",

SuperscriptBox[

RowBox[{"il1r", "[", "t", "]"}], "xla"]}],

RowBox[{

SuperscriptBox["kmla", "xla"], "+",

SuperscriptBox[

RowBox[{"il1r", "[", "t", "]"}], "xla"]}]]}], ")"}]}]}]},

{

RowBox[{

RowBox[{"replication", "[", "t", "]"}], "\[Equal]",

RowBox[{"kr", " ",

RowBox[{"(",

RowBox[{"1", "-",

FractionBox[

RowBox[{"vhr", " ",

SuperscriptBox[

RowBox[{"il1r", "[", "t", "]"}], "xhr"]}],

RowBox[{

SuperscriptBox["kmhr", "xhr"], "+",

SuperscriptBox[

RowBox[{"il1r", "[", "t", "]"}], "xhr"]}]], "+",

FractionBox[

RowBox[{"vlr", " ",

SuperscriptBox[

RowBox[{"il1r", "[", "t", "]"}], "xlr"]}],

RowBox[{

SuperscriptBox["kmlr", "xlr"], "+",

SuperscriptBox[

RowBox[{"il1r", "[", "t", "]"}], "xlr"]}]]}], ")"}]}]}]},

{

RowBox[{

RowBox[{"il1r", "[", "t", "]"}], "\[Equal]",

FractionBox[

RowBox[{"il1b", "[", "t", "]"}],

RowBox[{

RowBox[{"il1b", "[", "t", "]"}], "+",

RowBox[{"km", " ",

RowBox[{"(",

RowBox[{"1", "+",

FractionBox[

RowBox[{"il1ra", "[", "t", "]"}], "ki"]}], ")"}]}]}]]}]},

{

RowBox[{

RowBox[{

SuperscriptBox["il1b", "\[Prime]",

MultilineFunction->None], "[", "t", "]"}], "\[Equal]", "kplacebo"}]},

{

RowBox[{

RowBox[{

SuperscriptBox["il1ra", "\[Prime]",

MultilineFunction->None], "[", "t", "]"}], "\[Equal]", "0"}]}

},

GridBoxAlignment->{

"Columns" -> {{Left}}, "ColumnsIndexed" -> {}, "Rows" -> {{Baseline}},

"RowsIndexed" -> {}},

GridBoxSpacings->{"Columns" -> {

Offset[0.27999999999999997`], {

Offset[0.5599999999999999]},

Offset[0.27999999999999997`]}, "ColumnsIndexed" -> {}, "Rows" -> {

Offset[0.2], {

Offset[0.4]},

Offset[0.2]}, "RowsIndexed" -> {}}],

Column],

Function[BoxForm`e$,

TableForm[BoxForm`e$]]]], "Output",

CellChangeTimes->{3.6029396345957565`*^9}]

}, Open ]]

}, Closed]],

Cell[CellGroupData[{

Cell["Parameter values", "Subsection",

CellChangeTimes->{{3.602939696314001*^9, 3.602939698681587*^9}}],

Cell[CellGroupData[{

Cell[BoxData[

RowBox[{"params", "//", "TableForm"}]], "Input",

CellChangeTimes->{{3.6029397157938128`*^9, 3.602939720156891*^9}}],

Cell[BoxData[

TagBox[

TagBox[GridBox[{

{

RowBox[{"Kxg", "\[Rule]", "0.000016`"}]},

{

RowBox[{"Kxi", "\[Rule]", "0.05`"}]},

{

RowBox[{"Gh", "\[Rule]", "9"}]},

{

RowBox[{"vh", "\[Rule]", "4"}]},

{

RowBox[{"Ktr", "\[Rule]",

FractionBox["3", "25"]}]},

{

RowBox[{"Kin", "\[Rule]", "1.05`"}]},

{

RowBox[{"lambda", "\[Rule]", "0.743`"}]},

{

RowBox[{"Kglucose", "\[Rule]", "0.000292`"}]},

{

RowBox[{"vs", "\[Rule]", "0.7`"}]},

{

RowBox[{"kms", "\[Rule]", "0.021`"}]},

{

RowBox[{"taus", "\[Rule]", "0.5`"}]},

{

RowBox[{"kmf", "\[Rule]", "0.021`"}]},

{

RowBox[{"tauf", "\[Rule]", "0.5`"}]},

{

RowBox[{"vfg", "\[Rule]", "4"}]},

{

RowBox[{"xfg", "\[Rule]", "4"}]},

{

RowBox[{"kmfg", "\[Rule]", "9"}]},

{

RowBox[{"vf", "\[Rule]", "0.4`"}]},

{

RowBox[{"vlr", "\[Rule]", "1.8`"}]},

{

RowBox[{"kmlr", "\[Rule]", "0.0011`"}]},

{

RowBox[{"xlr", "\[Rule]", "3"}]},

{

RowBox[{"vhr", "\[Rule]", "2.7`"}]},

{

RowBox[{"kmhr", "\[Rule]", "0.018`"}]},

{

RowBox[{"xhr", "\[Rule]", "0.5`"}]},

{

RowBox[{"vla", "\[Rule]", "0.65`"}]},

{

RowBox[{"kmla", "\[Rule]", "0.00018`"}]},

{

RowBox[{"xla", "\[Rule]", "3"}]},

{

RowBox[{"vha", "\[Rule]", "4.6`"}]},

{

RowBox[{"kmha", "\[Rule]", "0.155`"}]},

{

RowBox[{"xha", "\[Rule]",

FractionBox["2", "3"]}]},

{

RowBox[{"km", "\[Rule]", "8.5`"}]},

{

RowBox[{"ki", "\[Rule]", "1.7`"}]},

{

RowBox[{"ka", "\[Rule]", "0.000552021517102881`"}]},

{

RowBox[{"kr", "\[Rule]", "0.0003763928481015982`"}]},

{

RowBox[{"kf", "\[Rule]", "0.009577538087791911`"}]},

{

RowBox[{"ks", "\[Rule]", "0.2910080871060985`"}]},

{

RowBox[{"Tgl", "\[Rule]", "0.025405`"}]},

{

RowBox[{"Kxgi", "\[Rule]", "0.000022404728593662437`"}]},

{

RowBox[{"il1bH", "\[Rule]", "0.05`"}]},

{

RowBox[{"il1b0", "\[Rule]", "4.834255667607401`"}]},

{

RowBox[{"kplacebo", "\[Rule]", "0.00137`"}]},

{

RowBox[{"k1", "\[Rule]", "0.2`"}]},

{

RowBox[{"k2", "\[Rule]", "0.0025`"}]}

},

GridBoxAlignment->{

"Columns" -> {{Left}}, "ColumnsIndexed" -> {}, "Rows" -> {{Baseline}},

"RowsIndexed" -> {}},

GridBoxSpacings->{"Columns" -> {

Offset[0.27999999999999997`], {

Offset[0.5599999999999999]},

Offset[0.27999999999999997`]}, "ColumnsIndexed" -> {}, "Rows" -> {

Offset[0.2], {

Offset[0.4]},

Offset[0.2]}, "RowsIndexed" -> {}}],

Column],

Function[BoxForm`e$,

TableForm[BoxForm`e$]]]], "Output",

CellChangeTimes->{3.602939720450964*^9}]

}, Open ]]

}, Closed]],

Cell[CellGroupData[{

Cell["Initial conditions", "Subsection",

CellChangeTimes->{{3.602939708073906*^9, 3.6029397116897993`*^9}}],

Cell[CellGroupData[{

Cell[BoxData[

RowBox[{"initConds", "//", "TableForm"}]], "Input",

CellChangeTimes->{{3.602940172672662*^9, 3.602940177177774*^9}}],

Cell[BoxData[

TagBox[

TagBox[GridBox[{

{

RowBox[{

RowBox[{"glucose", "[", "0", "]"}], "\[Equal]", "10.8`"}]},

{

RowBox[{

RowBox[{"insulin", "[", "0", "]"}], "\[Equal]", "100"}]},

{

RowBox[{

RowBox[{"b", "[", "0", "]"}], "\[Equal]", "40"}]},

{

RowBox[{

RowBox[{"il1ra", "[", "0", "]"}], "\[Equal]", "40.`"}]},

{

RowBox[{

RowBox[{"il1b", "[", "0", "]"}], "\[Equal]", "4.834255667607401`"}]},

{

RowBox[{

RowBox[{"proinsulin", "[", "0", "]"}], "\[Equal]",

"42.77755700003505`"}]},

{

RowBox[{

RowBox[{"f", "[", "0", "]"}], "\[Equal]", "0.042777557000035056`"}]},

{

RowBox[{

RowBox[{"isc", "[", "0", "]"}], "\[Equal]", "0.18528163580246915`"}]},

{

RowBox[{

RowBox[{"il1r", "[", "0", "]"}], "\[Equal]", "0.02266047547066035`"}]},

{

RowBox[{

RowBox[{"a1c1", "[", "0", "]"}], "\[Equal]", "0.12299724740572017`"}]},

{

RowBox[{

RowBox[{"a1c2", "[", "0", "]"}], "\[Equal]", "0.2442655436263679`"}]},

{

RowBox[{

RowBox[{"a1c3", "[", "0", "]"}], "\[Equal]", "0.36382919223184745`"}]},

{

RowBox[{

RowBox[{"a1c4", "[", "0", "]"}], "\[Equal]", "0.4817121551609546`"}]},

{

RowBox[{

RowBox[{"a1c5", "[", "0", "]"}], "\[Equal]", "0.5979380575236263`"}]},

{

RowBox[{

RowBox[{"a1c6", "[", "0", "]"}], "\[Equal]", "0.7125301923356859`"}]},

{

RowBox[{

RowBox[{"a1c7", "[", "0", "]"}], "\[Equal]", "0.8255115251870339`"}]},

{

RowBox[{

RowBox[{"a1c8", "[", "0", "]"}], "\[Equal]", "0.9369046988442165`"}]},

{

RowBox[{

RowBox[{"a1c9", "[", "0", "]"}], "\[Equal]", "1.0467320377882996`"}]},

{

RowBox[{

RowBox[{"a1c10", "[", "0", "]"}], "\[Equal]", "1.1550155526889523`"}]},

{

RowBox[{

RowBox[{"a1c11", "[", "0", "]"}], "\[Equal]", "1.2617769448156395`"}]},

{

RowBox[{

RowBox[{"a1c12", "[", "0", "]"}], "\[Equal]", "1.3670376103868085`"}]},

{

RowBox[{

RowBox[{"rbc1", "[", "0", "]"}], "\[Equal]", "8.62700275259428`"}]},

{

RowBox[{

RowBox[{"rbc2", "[", "0", "]"}], "\[Equal]", "8.505734456373633`"}]},

{

RowBox[{

RowBox[{"rbc3", "[", "0", "]"}], "\[Equal]", "8.386170807768153`"}]},

{

RowBox[{

RowBox[{"rbc4", "[", "0", "]"}], "\[Equal]", "8.268287844839046`"}]},

{

RowBox[{

RowBox[{"rbc5", "[", "0", "]"}], "\[Equal]", "8.152061942476374`"}]},

{

RowBox[{

RowBox[{"rbc6", "[", "0", "]"}], "\[Equal]", "8.037469807664314`"}]},

{

RowBox[{

RowBox[{"rbc7", "[", "0", "]"}], "\[Equal]", "7.924488474812966`"}]},

{

RowBox[{

RowBox[{"rbc8", "[", "0", "]"}], "\[Equal]", "7.8130953011557835`"}]},

{

RowBox[{

RowBox[{"rbc9", "[", "0", "]"}], "\[Equal]", "7.7032679622117`"}]},

{

RowBox[{

RowBox[{"rbc10", "[", "0", "]"}], "\[Equal]", "7.594984447311047`"}]},

{

RowBox[{

RowBox[{"rbc11", "[", "0", "]"}], "\[Equal]", "7.48822305518436`"}]},

{

RowBox[{

RowBox[{"rbc12", "[", "0", "]"}], "\[Equal]", "7.382962389613191`"}]},

{

RowBox[{

RowBox[{"hba1c", "[", "0", "]"}], "\[Equal]", "8.682143579043004`"}]}

},

GridBoxAlignment->{

"Columns" -> {{Left}}, "ColumnsIndexed" -> {}, "Rows" -> {{Baseline}},

"RowsIndexed" -> {}},

GridBoxSpacings->{"Columns" -> {

Offset[0.27999999999999997`], {

Offset[0.5599999999999999]},

Offset[0.27999999999999997`]}, "ColumnsIndexed" -> {}, "Rows" -> {

Offset[0.2], {

Offset[0.4]},

Offset[0.2]}, "RowsIndexed" -> {}}],

Column],

Function[BoxForm`e$,

TableForm[BoxForm`e$]]]], "Output",

CellChangeTimes->{3.602940177568871*^9}]

}, Open ]]

}, Closed]]

}, Closed]]

}, Open ]]

},

WindowSize->{1920, 1117},

WindowMargins->{{-8, Automatic}, {Automatic, -8}},

FrontEndVersion->"10.0 for Microsoft Windows (64-bit) (January 21, 2014)",

StyleDefinitions->"Default.nb"

]

(* End of Notebook Content *)

(* Internal cache information *)

(*CellTagsOutline

CellTagsIndex->{}

*)

(*CellTagsIndex

CellTagsIndex->{}

*)

(*NotebookFileOutline

Notebook[{

Cell[CellGroupData[{

Cell[580, 22, 165, 3, 101, "Title"],

Cell[748, 27, 184, 4, 56, "Subtitle"],

Cell[935, 33, 246, 5, 33, "Subsubtitle"],

Cell[CellGroupData[{

Cell[1206, 42, 153, 2, 86, "Section"],

Cell[CellGroupData[{

Cell[1384, 48, 114, 1, 49, "Subsection"],

Cell[1501, 51, 2114, 57, 228, "Input"],

Cell[3618, 110, 998, 17, 152, "Input"]

}, Closed]],

Cell[CellGroupData[{

Cell[4653, 132, 201, 3, 41, "Subsection"],

Cell[4857, 137, 13674, 346, 605, "Input"],

Cell[18534, 485, 1807, 29, 132, "Input"]

}, Closed]],

Cell[CellGroupData[{

Cell[20378, 519, 163, 2, 41, "Subsection"],

Cell[CellGroupData[{

Cell[20566, 525, 171, 2, 39, "Subsubsection"],

Cell[20740, 529, 743, 19, 115, "Input"],

Cell[21486, 550, 1055, 18, 92, "Input"]

}, Closed]],

Cell[CellGroupData[{

Cell[22578, 573, 266, 3, 33, "Subsubsection"],

Cell[22847, 578, 2591, 68, 126, "Input"],

Cell[25441, 648, 5824, 86, 292, "Input"]

}, Closed]],

Cell[CellGroupData[{

Cell[31302, 739, 211, 3, 33, "Subsubsection"],

Cell[31516, 744, 2387, 60, 144, "Input"],

Cell[33906, 806, 3134, 46, 232, "Input"]

}, Closed]]

}, Closed]]

}, Closed]],

Cell[CellGroupData[{

Cell[37101, 859, 212, 3, 56, "Section"],

Cell[37316, 864, 3012, 55, 192, "Input"],

Cell[40331, 921, 375, 10, 31, "Input"],

Cell[40709, 933, 2472, 55, 72, "Input"],

Cell[43184, 990, 238, 6, 31, "Input"]

}, Closed]],

Cell[CellGroupData[{

Cell[43459, 1001, 249, 3, 56, "Section"],

Cell[43711, 1006, 933, 22, 132, "Input"],

Cell[44647, 1030, 3511, 73, 72, "Input",

InitializationCell->True],

Cell[48161, 1105, 240, 6, 31, "Input"],

Cell[48404, 1113, 235, 6, 31, "Input"]

}, Closed]],

Cell[CellGroupData[{

Cell[48676, 1124, 128, 1, 56, "Section"],

Cell[48807, 1127, 1654, 44, 165, "Input"],

Cell[50464, 1173, 486, 10, 112, "Input"],

Cell[50953, 1185, 1552, 28, 31, "Input",

InitializationCell->True],

Cell[52508, 1215, 762, 22, 77, "Input"],

Cell[53273, 1239, 970, 25, 72, "Input"],

Cell[54246, 1266, 345, 9, 72, "Input"],

Cell[54594, 1277, 694, 14, 152, "Input"],

Cell[55291, 1293, 354, 9, 72, "Input"]

}, Closed]],

Cell[CellGroupData[{

Cell[55682, 1307, 94, 1, 56, "Section"],

Cell[55779, 1310, 355, 8, 31, "Input"],

Cell[56137, 1320, 343, 8, 31, "Input"],

Cell[56483, 1330, 579, 13, 31, "Input"],

Cell[57065, 1345, 382, 10, 31, "Input"],

Cell[CellGroupData[{

Cell[57472, 1359, 107, 1, 49, "Subsection"],

Cell[CellGroupData[{

Cell[57604, 1364, 140, 2, 31, "Input"],

Cell[57747, 1368, 21111, 596, 824, "Output"]

}, Open ]]

}, Closed]],

Cell[CellGroupData[{

Cell[78907, 1970, 103, 1, 41, "Subsection"],

Cell[CellGroupData[{

Cell[79035, 1975, 140, 2, 31, "Input"],

Cell[79178, 1979, 20036, 567, 824, "Output"]

}, Open ]]

}, Closed]],

Cell[CellGroupData[{

Cell[99263, 2552, 104, 1, 41, "Subsection"],

Cell[CellGroupData[{

Cell[99392, 2557, 131, 2, 31, "Input"],

Cell[99526, 2561, 2859, 103, 662, "Output"]

}, Open ]]

}, Closed]],

Cell[CellGroupData[{

Cell[102434, 2670, 108, 1, 41, "Subsection"],

Cell[CellGroupData[{

Cell[102567, 2675, 132, 2, 31, "Input"],

Cell[102702, 2679, 3923, 120, 556, "Output"]

}, Open ]]

}, Closed]]

}, Closed]]

}, Open ]]

}

]

*)

(* End of internal cache information *)
